# Hyperglycemia Affects miRNAs Expression Pattern during Adipogenesis of Human Visceral Adipocytes—Is Memorization Involved?

**DOI:** 10.3390/nu10111774

**Published:** 2018-11-15

**Authors:** Justyna Strycharz, Ewa Świderska, Adam Wróblewski, Marta Podolska, Piotr Czarny, Janusz Szemraj, Aneta Balcerczyk, Józef Drzewoski, Jacek Kasznicki, Agnieszka Śliwińska

**Affiliations:** 1 Department of Medical Biochemistry, Medical University of Lodz, 92-215 Lodz, Poland; justyna.strycharz@stud.umed.lodz.pl (J.S.); ewa.swiderska@stud.umed.lodz.pl (E.Ś.); adam.wroblewski@stud.umed.lodz.pl (A.W.); piotr.czarny@umed.lodz.pl (P.C.); janusz.szemraj@umed.lodz.pl (J.S.); 2 Department of Internal Diseases, Diabetology and Clinical Pharmacology, Medical University of Lodz, 92-213 Lodz, Poland; marta.kasinska@stud.umed.lodz.pl (M.P.); jacek.kasznicki@umed.lodz.pl (J.K.); 3 Department of Molecular Biophysics, University of Lodz, 90-236 Lodz, Poland; aneta.balcerczyk@biol.uni.lodz.pl; 4 Central Teaching Hospital of the Medical University of Lodz, 92-213 Lodz, Poland; jozef.drzewoski@umed.lodz.pl; 5 Department of Nucleic Acid Biochemistry, Medical University of Lodz, 251 Pomorska Str., 92-213 Lodz, Poland

**Keywords:** microRNA, expression profiling, visceral pre-adipocytes, adipocytes, adipogenesis, hyperglycemia, diabetes, hierarchical clustering, bioinformatics, “epi”-memory

## Abstract

microRNAs are increasingly analyzed in adipogenesis, whose deregulation, especially visceral, contributes to the development of diabetes. Hyperglycemia is known to affect cells while occurring acutely and chronically. Therefore, we aimed to evaluate the effect of hyperglycemia on human visceral pre/adipocytes from the perspective of microRNAs. The relative expression of 78 microRNAs was determined by TaqMan Low Density Arrays at three stages of HPA-v adipogenesis conducted under normoglycemia, chronic, and intermittent hyperglycemia (30 mM). Hierarchical clustering/Pearson correlation revealed the relationship between various microRNAs’ expression profiles, while functional analysis identified the genes and signaling pathways regulated by differentially expressed microRNAs. Hyperglycemia affected microRNAs’ expression patterns during adipogenesis, and at the stage of pre-adipocytes, differentiated and mature adipocytes compared to normoglycemia. Interestingly, the changes that were evoked upon hyperglycemic exposure during one adipogenesis stage resembled those observed upon chronic hyperglycemia. At least 15 microRNAs were modulated during normoglycemic and/or hyperglycemic adipogenesis and/or upon intermittent/chronic hyperglycemia. Bioinformatics analysis revealed the involvement of these microRNAs in cell cycle, lipid metabolism, ECM–receptor interaction, oxidative stress, signaling of insulin, MAPK, TGF-β, p53, and more. The obtained data suggests that visceral pre/adipocytes exposed to chronic/intermittent hyperglycemia develop a microRNAs’ expression pattern, which may contribute to further visceral dysfunction, the progression of diabetic phenotype, and diabetic complications possibly involving “epi”-memory.

## 1. Introduction

Adipogenesis (ADG) is a process composed of two major phases, involving mesenchymal stem cells’ (MSC) differentiation into fat cells [1]. Firstly, pluripotent mesenchymal stem cells (MSCs) from the vascular stroma of adipose tissue (AT) respond to the external signals and develop into pre-adipocytes (pAds). In the differentiation phase, the exposure of pAds to hormones (insulin, glucocorticoids) and growth factors (insulin-like growth factor 1—IGF1) promotes the activation of numerous transcription factors, including CCAAT/enhancer binding proteins (C/EBPs) and peroxisome proliferator activated receptor-γ (PPARγ), which activates the expression of the genes that are typical of mature adipocytes (Ads) such as leptin, adiponectin etc. The deregulation of ADG involves adipocyte hypertrophy and hyperplasia, indicating their enlargement and de novo differentiation, respectively [2,3]. Expanded AT is dysfunctional and predisposes to inflammation, insulin resistance (IR), and type 2 diabetes mellitus (T2DM). Considering the differences between ATs that are located subcutaneously (SAT) and viscerally (VAT), VAT mass expansion is particularly associated with metabolic disorders [4]. It is noteworthy that visceral Ads show increased lipolysis, sensitivity to catecholamines, and resistance to insulin. The expansion of visceral fat (measured as the waist-to-hip ratio, WHR) is a strong predictor for T2DM development, which is inseparably connected with raised blood glucose levels [4]. Hyperglycemia (HG) exerts an impact on each body cell while occurring chronically and acutely, including mature adipocytes treated with high and normal glucose interchangeably for 24 h [5,6]. HG is infamous for being especially detrimental to endothelial cells, where it promotes metabolic memory via epigenetic mechanisms that are mediated, among others, by microRNAs (miRNAs) [7,8,9]. These short non-coding RNAs predominantly serve as negative regulators of gene expression at the mRNA (decay) and protein level (translation repression), simultaneously regulating numerous targets [9]. Current data indicates changes in levels of miRNAs during ADG and upon diet, obesity, HG, and diabetes [10,11]. Moreover, high glucose has been recognized as a factor epigenetically priming genes associated with inflammation in adipose progenitor cells, with the potentiation of this effect upon the accomplishment of adipocyte differentiation [12]. From our point of view, this study provided a strong implication of the particular sensitivity of premature forms of adipocytes to environmental stimuli. As adipogenesis occurs throughout the life, adipocytes can be exposed to high glucose levels at each stage of their development, not only in fetuses carried in wombs of diabetic mothers, but also in metabolically deregulated adults. This also made us speculate as to whether there is a stage of adipogenesis that could be of the most relevance for the introduction of changes that are typical of the diabetic phenotype. We assumed that the pattern of miRNAs expression could be changed upon HG-affected ADG, and asked whether these molecules could be involved in the memorization of the effect of HG in p/Ads. Therefore, we aimed to determine the miRNAs expression profiles during the proliferation, differentiation, and maturation of human visceral p/Ads occurring physiologically and upon chronic and intermittent HG.

## 2. Materials and Methods 

### 2.1. Cell Culture and Treatment 

**Figure 1:**
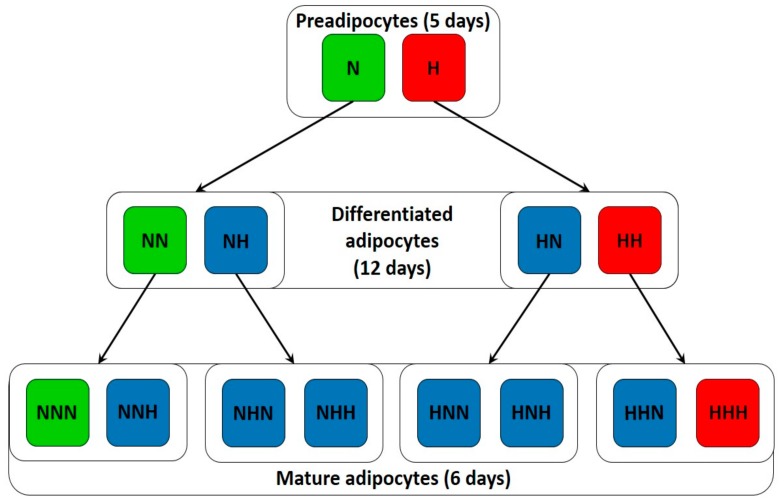
The stages of adipogenesis of HPA-v cells carried out under normoglycemia (NG, green), chronic hyperglycemia (HG, red) and intermittent HG (blue). While “H” denotes hyperglycemic treatment, “N” indicates normoglycemic culture conditions.

Human visceral pAds (HPA-v, ScienCell Research Laboratories, Carlsbad, CA, USA) were cultured in monolayer (95% humidity, 5% CO_2_, 37 °C) in poly-l-lysine-coated flasks (10 mg/mL) using reagents from ScienCell Research Laboratories, and in accordance with the attached protocol. The experiment involved three stages—pAds culture for five days in PAM (preadipocyte medium), pAds differentiation in PADM (preadipocyte differentiation medium) for 12 days, and the maintenance of mature Ads for six days in AdM (adipocyte medium). Three independent experiments were conducted. The accomplishment of differentiation was confirmed by the presence of lipid droplets under microscope. Cell culture was performed under normoglycemia (NG), as well as chronic and intermittent HG (30 mM), which generated 14 culture variants. The flow chart visualizing the steps of cell culture, as well as the number and order of hyperglycemic exposure, is presented in Figure 1. We suggest “H” and “N” to indicate the hyperglycemic and normoglycemic conditions at the particular culture stage, respectively. For instance, “NNN” cells were cultured solely in full media for 23 days, while “HNN” cells were subjected to hyperglycemic treatment only during first five days of culture (during the proliferation of pAds). For the interpretation of results, all three variants that were solely treated with HG (H, HH, and HHH) were planned to reflect adipogenesis in a diabetic condition, while all three variants overly untreated with HG (N, NN, NNN) were to reflect a physiological one. Other variants were designed to examine (i) the relevance of hyperglycemic exposure on each stage of adipogenesis, (ii) the selection of the step that is critical for introduction of changes that are typical of the diabetic phenotype, and (iii) the effect of the normalization of glycemia. Hyperglycemic conditions were obtained via the supplementation of full media with glucose (d-(+)-Glucose, Sigma-Aldrich (St Louis, MO, USA)) in an amount allowing for the accomplishment of 30-mM concentration. To provide relatively stable glucose concentration, the medium (PAM) was changed every two days during the proliferation of pAds and every three days for the other culture stages (PADM, ADM). 

### 2.2. miRNA Expression Profiling

miRNA was isolated from 5 × 10^6^ cells using an miRVANA Isolation Kit according to manufacturer’s instructions and supplied by Life Technologies (Vilnius, Lithuania). All other reagents needed for expression profiling were supplied by Applied Biosystems (Foster City, CA, USA). The selection of miRNAs for further analysis was performed via the expression profiling of 754 miRNAs in pAds (N, H) and mature Ads (NNN, HHH) using commercially available TaqMan^®^ low-density arrays (TLDA) (TaqMan^®^ Array Human MicroRNA A+B Cards Set v3.0). Firstly, we performed a reverse transcription (RT) reaction in Tpersonal Thermocycler (Biometra, *Göttingen,* Germany) in the following conditions: step I—40 cycles including two min at 16 °C, 1 min at 42 °C, and one s at 50 °C, step II—RT inactivation—two min at 85 °C, and step III—cooling at 4 °C, using a TaqMan MicroRNA Reverse Transcription Kit and Megaplex™ Primer Pools, Human Pools Set v3. in accordance with the attached protocols. Next, we used TaqMan^®^ Array Human MicroRNA A+B Cards Set v3.0 and TaqMan^®^ Universal PCR Master Mix II, no UNG to conduct real-time PCR in 7900HT Fast Real-Time PCR System (Applied Biosystems; Thermo Fisher Scientific, Inc., Waltham, MA, USA)) in the conditions specified for TLDA cards. Based on the results of the screening analysis obtained due to normalization to a U6 snRNA level, we designed custom TLDA cards with molecules presented in Material S1 (Table S1). Following the cell culture of 14 variants and miRNA isolation, we conducted reverse transcription reaction, whose components were the same as those used for screening analysis, except for primers. Namely, Primers Pool adapted to customized TLDA cards were employed. Some samples of the same biological replicate were pooled. The following RT reaction conditions were applied: preheating for 30 min at 16 °C, cDNA synthesis for 30 min at 42 °C, RT inactivation for five min at 85 °C, and cooling at 4 °C. Subsequently, the RT reaction product was mixed with TaqMan^®^ Universal PCR Master Mix II, no UNG and RNAse-free water. Then, 100 µL of real-time PCR mixture was loaded into each port of a customized MicroRNA TLDA Card, which was then centrifuged and sealed to carry out quantitative real-time PCR of each assay in duplicate in conditions indicated by the manufacturer. The relative expression of 78 miRNAs was determined using the ΔCt method [13]. For internal control, we used an arithmetic average of U6snRNA and let-7b-5p, which was supported by RefFinder [14]. Expression profiles and statistics were provided to examine the impact of only one variable, either ADG, chronic HG, or a single, stage-specific HG hit. 

### 2.3. Functional Analysis 

We performed miRNA enrichment analysis on differentially expressed miRNAs with DIANA-miRpath v3.0 [15], miRSystem [16], and miEAA [17]. Using known or predicted interactions between miRNAs and target genes, each software allowed for the elucidation of molecular pathways controlled by miRNAs. Considering DIANA-miRPath, we performed KEGG (Kyoto Encyclopedia of Genes and Genomes) analysis, and merged results by genes and pathways with default settings. Our analysis was based on experimentally validated interactions (Tarbase) or the microT-CDS algorithm, provided that the particular miRNA was not found in Tarbase. In miEAA, we conducted an overrepresentation analysis without a reference set using “pathways (miRWalk)”, FDR (false discovery rate) adjustment, an 0.05 significance level, and a “1” threshold level. In miRSystem, functional annotation was conducted with default settings. Using miRTargetLink Human, we retrieved strongly experimentally supported and shared target genes for selected miRNAs, and provided the interaction network [18]. 

### 2.4. Statistical Analysis 

We clustered and correlated data using hierarchical clustering and Pearson’s correlation with Gitools2.3.1 [19], respectively. A two-tailed student t-test was used for the comparison of two mean 2^-ΔCt^ values and one-way ANOVA with a post hoc Tukey test for multiple pairwise comparisons (GraphPad Prism 6.0 (La Jolla, CA, USA)). *p* ≤ 0.05 was considered as significant. Data was presented as mean ± SD. 

## 3. Results

### 3.1. Chronic HG Modified miRNAs Expression Pattern in HPA-v ADG 

**Figure 2:**
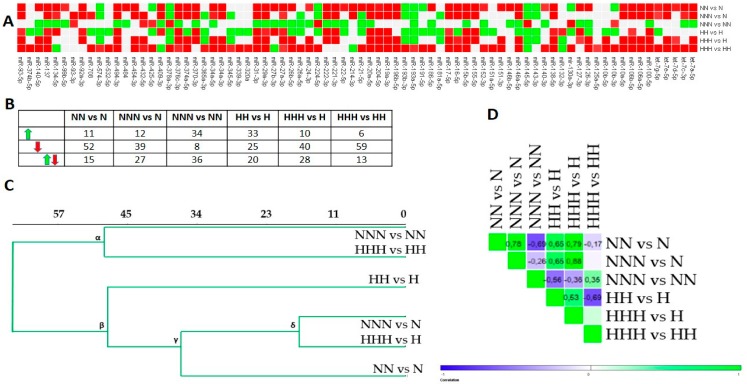
The changes of microRNAs (miRNAs)’ expression in differentiated Ads and mature Ads cultured in NG and HG during adipogenesis (ADG), based on FC (fold change) values. (**A**) The heat map diagram expression changes of examined miRNAs in differentiated adipocytes (Ads) (NN vs. N, HH vs. H), mature Ads in relation to pre-adipocytes (pAds) (NNN vs. N, HHH vs. H) and differentiated Ads (NNN vs. NN, HHH vs. HH). Red denotes expression decline (FC < −1.5), green indicates expression increase (FC >1.5) and grey shows no changes. (**B**) Table depicts the numerical summary of miRNAs changes from the heat map in (**A**). (**C**) Euclidean hierarchical clustering of miRNAs expression profiles. (**D**) Heat map shows positive (green) and negative (blue) Pearson’s correlation coefficients for comparisons of variants differentiated in NG and HG.

The miRNAs expression profiles that were detected during the differentiation and maturation of HPA-v pAds in NG and HG are presented in Figure 2A,B. The second stage of completion in NG (NN vs. N) revealed the downregulation of the majority of the miRNAs. While we observed that nearly a half of the molecules upregulated after transition from NN to NNN, the reverse was shown for exactly 50% of the studied miRNAs in NNN vs. N. Considering ADG in HG, the completion of the second stage (HH vs. H) showed no dominant direction of miRNAs expression changes, yet a small majority of miRNAs was decreased in HHH vs. H. Oppositely, a large majority of miRNAs was declined in HHH vs. HH. 

Next, we performed Euclidean hierarchical clustering (Figure 2C), and correlated expression profiles via Pearson correlation (Figure 2D). Our first analysis revealed two major clusters with an identical intragroup similarity (distance). The most separate cluster (α) was formed with NNN vs. NN and HHH vs. HH, which were weakly correlated (*r* = 0.35) and showed disparate numbers of changed miRNAs (Figure 2B–D). β indicated similarity among the miRNAs expression patterns of NG and HG cultured differentiated and mature Ads in relation to pAds, which was further confirmed by several moderate to high positive correlation coefficients (Figure 2C,D). However, HH vs. H presented an expression profile that was the least compatible with the rest of β. Interestingly, the δ cluster’s components (NNN vs. N and HHH vs. H) were the most positively correlated ones in general (*r* = 0.88), suggesting that HG is unlikely to alter the general direction of the miRNAs expression changes in mature Ads during ADG (Figure 2B,D). We also observed a substantial negative correlation (*r* = −0.69) for NNN vs. NN/NN vs. N and HHH vs. HH/HH vs. H, implying that the changes evoked by HG occurred in differentiated and mature Ads (Figure 2D). A lower positive correlation was found for NN vs. N/HH vs. H, showing a substantial magnitude of similarity for the changes detected in differentiated Ads (Figure 2D).

Moreover, to find the miRNAs that are shared between different stages of ADG, either in NG or HG, significantly expressed miRNAs of three sets were combined and presented in Material S1 (Figure S1). Interestingly, only miR-140a-5p, miR-193a-5p, and miR-29a-3p were changed during normoglycemic and HG-affected ADG. 

### 3.2. Intermittent and Chronic HG Deregulated the miRNAs Expression Pattern in pAds, Differentiated Ads, and Mature Ads

**Figure 3:**
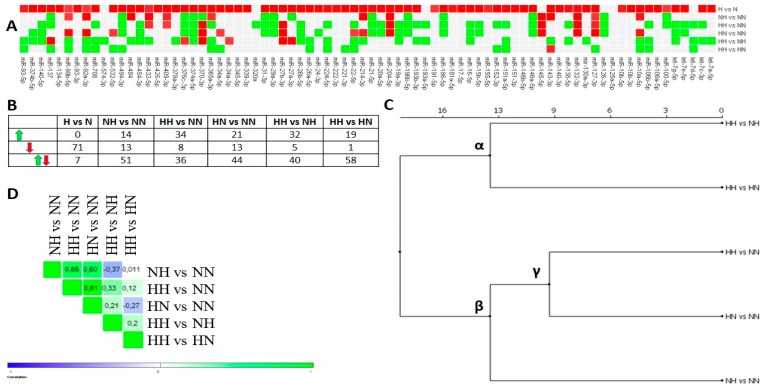
miRNAs expression changes evoked by chronic and intermittent HG in pAds and differentiated Ads, based on FC values. (**A**) The heat map diagram shows the expression changes of examined miRNAs in pAds and differentiated Ads exposed to HG. Red denotes an expression decline (FC < −1.5), green indicates an expression increase (FC > 1.5) and grey shows no changes. (**B**) Table depicts the numerical summary of miRNAs changes from the heat map in (**A**). (**C**) Euclidean hierarchical clustering of studied comparisons. (**D**) Heat map shows positive (green) and negative (blue) Pearson’s correlation values for pairwise comparisons of variants of differentiated Ads exposed to a single or double stimulus of HG.

HG-treated pAds exhibited the decline of nearly all of the miRNAs (H vs. N), while the HH Ads expressed nearly a half of them at the increased level (HH vs. NN) (Figure 3A,B). Numerous miRNAs were unaffected in differently HG-treated and differentiated Ads (NH, HN, HH), when considering all of the presented comparisons. Their hierarchical clustering showed two major clusters (α, β), where the β cluster’s components indicated positively and strongly correlated sets of miRNAs expression profiles (*r* >0.6). The β-derived cluster, γ, was made up of highly correlated HH vs. NN and HN vs. NN (*r* = 0.81), suggesting that differentiated Ads could possibly “remember” the effect of HG exposure during the proliferation of pAds, being reflected in highly similar expression profiles (Figure 3B–D).

**Figure 4:**
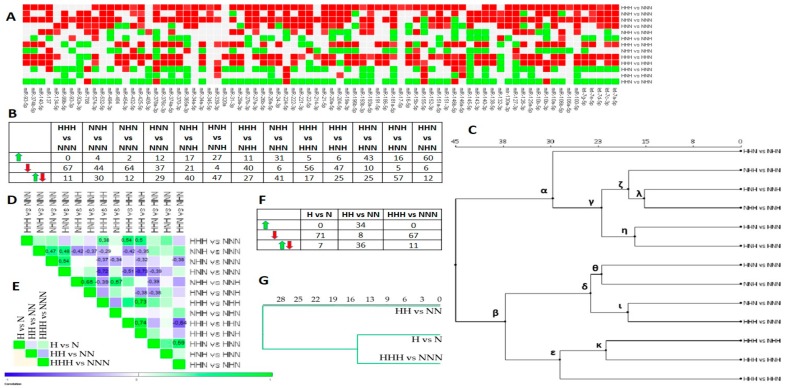
miRNAs expression changes triggered by chronic and intermittent HG in mature Ads, based on FC values. (**A**) The heat map diagram shows the expression changes of the examined miRNAs in mature Ads exposed to chronic and intermittent HG. Red denotes an expression decline (FC < −1.5), green indicates an expression increase (FC >1.5), and grey shows no changes. (**B**) Table depicts a numerical summary of the miRNAs changes from the heat map in (**A**). (**C**) Euclidean hierarchical clustering of studied comparisons. (**D**). Heat map shows positive (green) and negative (blue) Pearson’s correlation coefficients for pairwise comparisons of variants of mature Ads exposed to chronic or intermittent HG stimulation. (**E**) Heat map presents positive (green) and negative (blue) Pearson’s correlation coefficients for pairwise comparisons of variants of pAds, as well as differentiated and mature Ads exposed to chronic HG. (**F**) Table displays a numerical summary of miRNAs changes from the heat map in (**E**). (**G**) Euclidean hierarchical clustering of comparisons from (**E**).

The impact of HG on the miRNA expression pattern in mature Ads is depicted in Figure 4A,B. The analysis presented in Figure 4C showed two major clusters (α,β). α grouped solely comparisons, visualizing the impact of double versus single HG stimulus. First, correlation-based analysis confirmed only ζ cluster’s similarity (*r* ≈ 0.61). Second, several miRNAs profiles from α were positively correlated when related to the same variant, as seen for HHN vs. NHN/HHN vs. HNN (*r* = 0.59). This may imply that independently of ADG stages, the exposure to a double HG stimulus may evoke similar miRNAs expression changes in comparison to a single stimulus.

**Figure 5:**
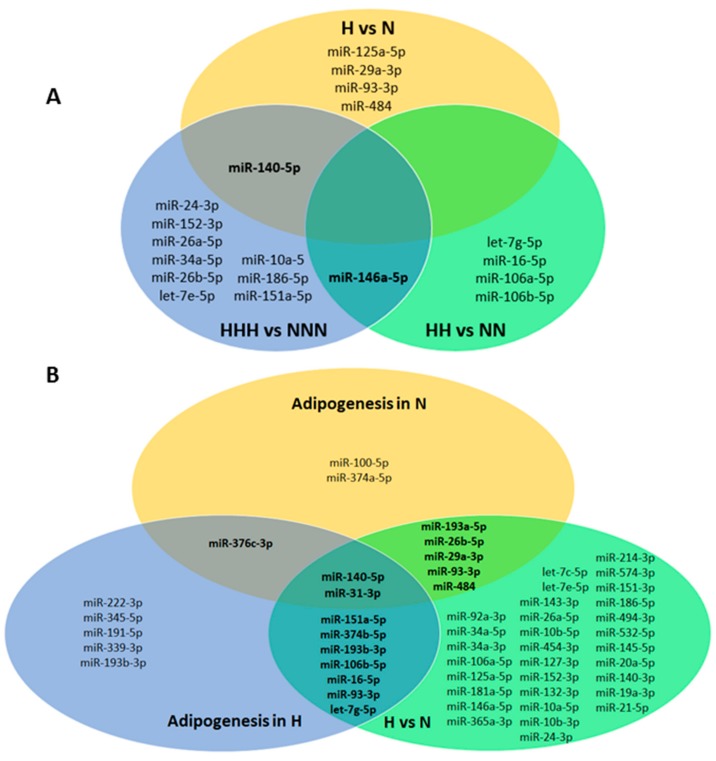
miRNAs significantly changed upon HG and ADG. (**A**) Venn diagram shows significantly changed miRNAs upon chronic HG. (**B**) Venn diagram presents significantly changed miRNAs during ADG in NG and HG as well as upon chronic or intermittent HG. Shared miRNAs are bolded. Statistical significance was evaluated using two-tailed t-test with *p* ≤ 0.05 considered as significant.

The second major cluster, β, was subdivided into δ and ε (Figure 4C). While δ showed sets of comparisons uncovering the impact of single and chronic HG stimulus versus NG, ε grouped these showing the influence of a chronic versus a double HG hit. Regarding δ, we observed the predominance of downregulated miRNAs, which was the most pronounced for HHH vs. NNN (Figure 5A,B). Interestingly, the sub cluster of δ, ι, revealed a similarity between the miRNAs profiles of HHH vs. NNN and NHN vs. NNN, which was not reflected in correlation analysis, yet could be easily inferred from Figure 5B. Moreover, we observed several moderate correlation coefficients confirming the similarity among all of the comparisons involving only a single stimulus of HG versus chronic NG (Figure 4D). These results suggest that even a single exposure to HG during ADG might exert miRNAs expression changes that are similar to those found in Ads exposed chronically to HG.

Considering ε, it was composed of expression patterns enriched with numerous declined miRNAs, whose comparisons obtained the highest positive correlation coefficients (*r* ≈ 0.74) in mature Ads. Components of the ε cluster were moderately and positively correlated with HHH vs. NNN (*r* ≈ 0.48), yet highly and negatively correlated with HNN vs. NNN (*r* ≈ 0.65). This may further explain the structure of the β cluster. We regard the β cluster to be informative enough to imply that even a single hit of HG may trigger the decline of numerous miRNAs, and that the restoration of NG is not sufficient to entirely reverse the impact of HG. 

Next, we asked whether chronically HG-exposed pAds as well as differentiated and mature Ads presented any kind of similarity regarding miRNAs expression patterns. First, correlation analysis (Figure 4E) indicated entirely different miRNAs expression profiles. Second, we could observe a consistent direction of miRNAs expression changes between HG-treated pAds and mature Ads (Figure 4F), which was also suggested by clustering (Figure 4G). 

Next, we used a Venn diagram to show the miRNAs that were significantly modulated by even one HG stimulus at the particular ADG stage (Material S1, Figure S2). Moreover, in Figure 5, we exclusively presented the miRNAs that were significantly modulated by chronic HG (Figure 5A), as well as a compilation of all of the significantly regulated miRNAs upon HG and during ADG (Figure 5B). The latter approach allowed for the selection of 15 core miRNAs with miR-140-5p and miR-31-3p being shared by all three sets. In Material S1 (Figures S3–S5), we also showed an in-depth analysis of the expression changes of 9 out of 15 core miRNAs, as these exhibited either novelty in the research field, a high level of differential expression, or showed signs of the memory of HG (i.e., miR-151a-5p). Additionally, we chose to present miRNAs with a strong implication of memorization of the HG-triggered effect (miR-10a-5p).

### 3.3. Enrichment Analysis 

**Figure 6:**
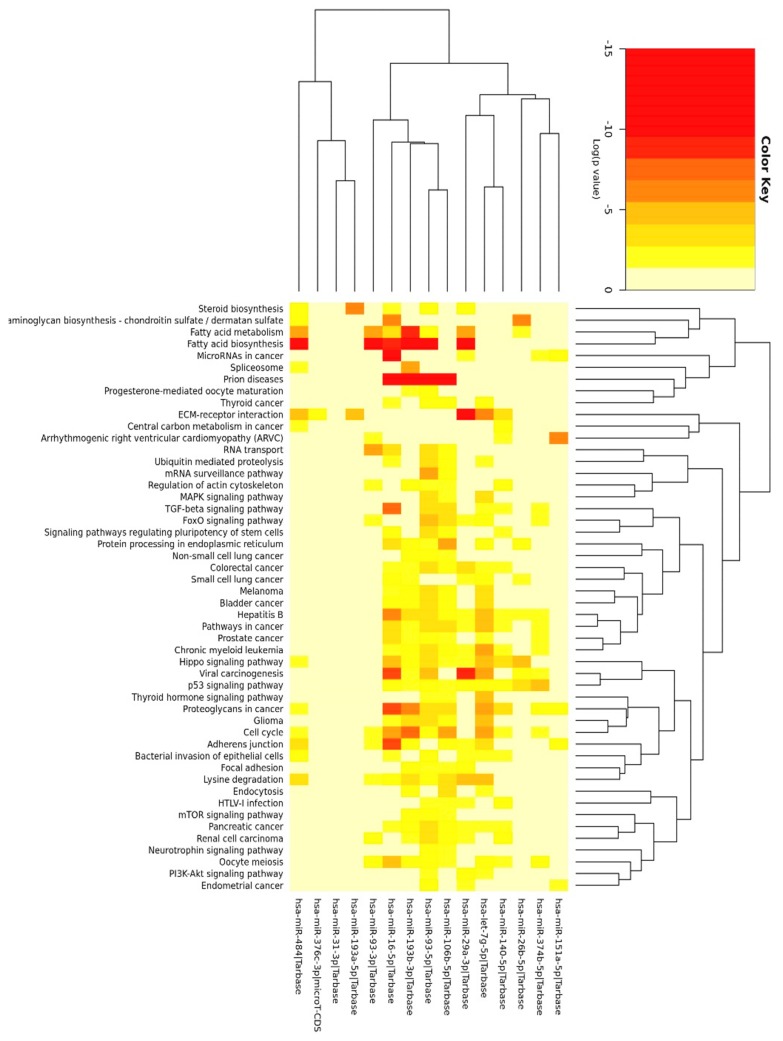
The heat map of results generated by DIANA-miRPath v3.0 and merged by pathways’ union for 15 core miRNAs (significance clusters). Full name of the second record is “Glycosaminoglycan biosynthesis: chondroitin sulfate / dermatan sulfate”.

We performed miRNA enrichment analysis on 15 shared miRNAs depicted in Figure 6C in mid-October 2018. Genes union analysis (DIANA-miRPath) (Material S1, Table S2) implied that the selected miRNAs could be involved in the regulation of lipid metabolism (metabolism, elongation and biosynthesis of fatty acids, steroid biosynthesis) and insulin signaling (MAPK (mitogen activated kinase) pathway, inositol phosphate metabolism, phosphatidylinositol signaling system, mTOR (mammalian target of rapamycin) and FOXO (forkhead box protein O) signaling pathways). Bioinformatics analysis also indicated pathways modulated by hormones, growth factors, cytokines (estrogen, TGF-β (tumor growth factor), TNF-α (tumor necrosis factor)), and associated with the transcription factors that are crucial for T2DM and/or ADG (HIF-1α (hypoxia-inducible factor), p53). The top records visualized the importance of the cell cycle, extracellular matrix-receptor interactions, adherence junction and Hippo signaling, which are all critical for ADG. The results that were generated due to genes and pathways’ union (Material S1, Table S3) were similar, yet the latter more profoundly suggested the involvement of lipid metabolism and p53 signaling (Figure 6). The results provided by DIANA-miRPath and miEAA (Material S1, Table S4) were consistent, particularly implying the role of selected miRNAs in the regulation of TGF-β signaling, lipid metabolism, and insulin signaling. Interestingly, the top records by miEAA showed MAPK signaling and DNA replication. We observed numerous terms connected with oxidative stress, DNA repair, cell cycle checkpoints, and ATM (Ataxia Telangiectasia Mutated)-dependent response to DNA damage along with the signaling of cytokines, adipocytokines (8/15 miRNAs), leptin (9/16 miRNAs), interleukin (IL)-1/4/6/7, as well as ADG (10/15 miRNAs) and T2DM (8/16 miRNAs). miRSystem provided results underscoring vesicle and membrane-associated events along with focal adhesion (Material S1, Table S5). 

**Figure 7:**
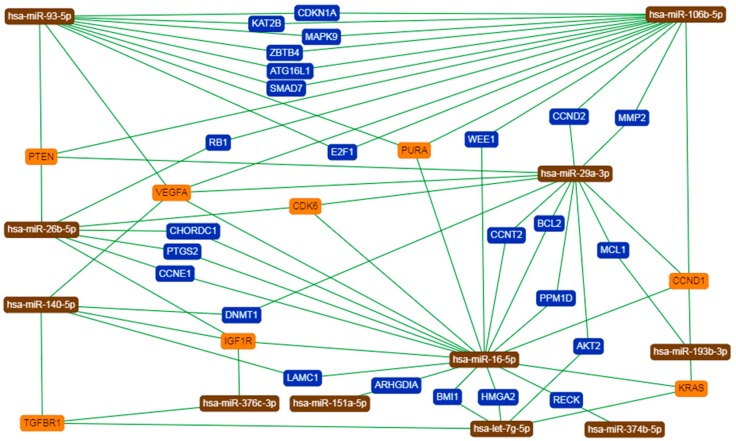
Network of interactions between several core miRNAs and their validated target genes provided by miRTargetLink Human. Orange describes genes interacting with at least three miRNAs, while blue shows genes targeted by two miRNAs.

For better understanding of the function of selected miRNAs, we presented the top three records generated by DIANA-miRPath for each of 15 core miRNAs (Material S1 (Table S6)). We next asked whether there are some genes that could be regulated by more than one selected miRNA. Thus, we applied miRTargetLink Human to present an integrated network for miRNAs and their shared target genes, being supported exclusively by strong experimental evidence (Figure 7). As presented in Material S2, among all of the indicated targets, there were genes that were strongly involved in the regulation of cell cycle and apoptosis and ECM remodeling along with insulin, MAPK, TGF-β, and p53 signaling. 

### 3.4. Functional Analysis of 11 miRNAs Changed upon Chronic HG in Mature Adipocytes

We also performed bioinformatics analysis on 11 miRNAs, which were significantly changed in mature adipocytes upon chronic HG in comparison to NG (HHH vs. NNN) (Figure 5A). Both examined sets shared only miR-140-5p, miR-151a-5p, and miR-26b-5p (Figure 5). While DIANA-miRPath showed similar results between the two analyses, vast differences were recorded using miEAA and miRSystem (Material S1, Table S7–10). For instance, among several top records by miEAA, we could notice “fatty acid biosynthesis”, “steroid biosynthesis”, “miRNAs in cardiomyocytes hypertrophy”, “TFs (transcription factors) regulate miRNAs related to cardiac hypertrophy” and “endocytosis”. In contrast to previous analysis, the records associated with TGF-β, MAPK, and insulin signaling appeared to have lower relevance. The results generated by the miRSystem were, in general, in line with miEAA and DIANA-miRPath. However, the number of statistically significant records was increased in comparison to the respective analysis of 15 core miRNAs. Importantly, among the top records by miRSystem, we could observe those associated with mitosis, focal adhesion, the signaling of Wnt (Wingless-Type), LKB1 (Liver kinase B1), and interleukins. Interestingly, we also observed the record including seven union miRNAs called “transcriptional regulation of white adipocyte differentiation”, in which the targeting of KLF4 (Kruppel Like Factor 4), WNT1, and CDK8(cyclin dependent kinase 8) by five, four, and three miRNAs was indicated, respectively.

## 4. Discussion

We determined the expression level of 78 miRNAs in human visceral pAds, differentiated and mature Ads exposed to NG, and chronic and intermittent HG. Considering physiological ADG, we observed the decline of the majority of miRNAs in mature and differentiated Ads in comparison to pAds, which stayed in line with the data provided for subcutaneous Ads differentiated from human stromal cells [20]. Regarding the effect of HG, the most marked difference was shown for mature Ads in relation to differentiated Ads; however, the results as a whole indicated that HG evoked either a disparate direction or a magnitude of miRNAs expression changes in comparison to NG. These observations are in agreement with existing data suggesting that miRNAs in AT are strongly deregulated in response to HG [21,22]. HPA-v Ads were sensitive to both intermittent and chronic HG at all three stages of development. We detected the strong overrepresentation of declined miRNAs in pAds and mature Ads treated entirely with HG, while the most diverse miRNAs changes were observed for the differentiated Ads. Concordantly, miRNAs modulated during ADG of 3T3-L–1 cells were declined in mature Ads from obese mice [23]. Ortega et al. demonstrated that the miRNAs profile established for human pAds and mature Ads showed a close crosstalk between miRNAs and ADG, while selected miRNAs were indicated as candidates for biomarkers and therapeutic targets for obesity and obesity-related complications such as diabetes [11]. Our data revealed that 15 miRNAs i.e., miR-193b-3p, miR-193a-5p, miR-374b-5p, miR-16-5p, let-7g-5p, miR-376c-3p, miR-26b-5p, miR-29a-3p, miR-93-3p, miR-484, miR-140-5p, miR-31-3p, miR-93-5p, miR-151a-5p, and miR-106b-5p were significantly changed both during ADG and upon HG. Another high throughput study revealed that 376c-3p, miR-16-5p, miR-374b-5p, miR-31-3p, miR-140-5p, and let-7g-5p to be significantly regulated during human subcutaneous ADG [11]. Moreover, at least a threefold reduction of miR-151a-5p, miR-26b-5p, miR-106b-5p, miR-376c-3p, miR-193a-5p, miR-374b-5p, and miR-93-5p was detected for differentiated subcutaneous Ads in relation to pre-adipocytes [20]. The ADG of mouse embryonic SCs (CGR8) was accompanied by differentially expressed miR-16-5p, miR-140-5p, miR-193b-3p, let-7g-5p, miR-26b-5p, and miR-29a-3p at the stage of either mesodermal progenitor cells, pAds, or Ads [24]. Considering further mice cells, changes of let-7g-5p, miR-16-5p, miR-29a-3p, and miR-26b-5p were demonstrated during 3T3-L1 differentiation [25,26]. Moreover, 3T3-L1 cells overexpressing C10orf116, which is the gene that is critical for ADG, exhibited the differential expression of let-7g-5p, miR-26b-5p, miR-31-3p, miR-106b-5p, miR-29a-3p, miR-151a-5p, and miR-374b-5p [27]. Consistent with our data, mice with a knockout of miR-93-5p and miR-106b-5p displayed the promotion of ADG, increased visceral adiposity, and IR [28]. Possibly, we are the first to identify the expression changes of miR-484, mir-370-3p, miR-339-3p, and miR-93-3p in white ADG, and many more specifically in the visceral one. Intriguingly, nearly all of the 15 selected miRNAs were previously indicated as responsive toward HG, diabetes, or obesity in white AT or Ads, except for miR-374b-5p, miR-151a-5p, and miR-193a-5p [11,21,29,30,31,32,33,34]. However, our results are not entirely consistent with previous findings, which may arise from the following differences: those between in vivo and in vitro studies (complete AT with inflammatory cells versus mere Ads), interspecies ones (studies on 3T3-L1 mice cells) and finally, those existing between subcutaneous and visceral Ads. We also provided further discussion concerning 15 core miRNAs and miR-10a-5p, which is the obesity-responsive miRNA in subcutaneous pAds and Ads, whose expression changes may reflect an involvement in the memorization of the HG-mediated effect (Material S3) [11,25,35,36]. It is noteworthy that a similar expression profile in mature Ads was detected also, for instance, for miR-34a-5p. It constitutes the most prominent miRNA in an impressive network of a potent IR inducer, p53 (see Figure 4) [37,38]. We possibly revealed the responsiveness of several miRNAs toward HG, including miR-193a-5p, mir-374b-5p, miR-34a-3p, let-7c-5p, miR-454-3p, miR-127-3p, miR-132-3p, miR-10b-3p, miR-574-3p, miR-151-3p, miR-19a-3p, miR-93-3p, miR-106a-5p, miR-152-3p, and miR-186-5p, which were not indicated earlier as changed upon HG, diabetes, and obesity. As visceral Ads constitute a less examined type of white fat cells, we have plausibly reported numerous miRNAs that are responsive to intermittent or chronic HG in visceral Ads for the first time. 

Our functional analysis indicated the involvement of 15 selected miRNAs in the regulation of lipid metabolism, insulin signaling, cell cycle, ECM–receptor interaction, oxidative stress, and DNA repair (Figure 6, Tables S2–S5), which is in agreement with the current knowledge regarding HG-mediated cellular effects and the phenomena that are typical of ADG [37,39,40]. Considering signaling pathways, we observed those associated with TGF-β, p53, and MAPK to be the most highly enriched ones. Indeed, these are widely known to participate in the physiology and pathophysiology of Ads [37,40,41,42]. Bioinformatics analysis also underscored the well-known endocrine function of Ads, showing records with IL-6, leptin, adiponectin, etc. [5]. To better understand the role of each of the 15 selected miRNAs, we presented the top three records for each molecule (DIANA-miRPath) (Material S1, (Table S7)). This further suggested their prominent role in the regulation of the cell cycle, p53 signaling, and the biosynthesis of lipids and steroids [43]. Although records such as “viral carcinogenesis” or “prion diseases” might be regarded as counterintuitive ones, they possess genes associated with MAPK signaling, cell cycle, stress response, p53 signaling, and more. Our next analysis was performed with MirTargetLink Human. The obtained results further confirmed our observations; however, we noticed its two limitations. Firstly, due to a lack of shared targets, not all of the miRNAs that were initially included in the analysis were indicated in the final network. Secondly, not all of the experimentally validated target genes were shown. For instance, GLUT-4 was earlier proved to be regulated by miR-93-5p and miR-106b-5p, while miR-374b-5p was shown to target PTEN [44,45]. Nevertheless, we found VEGFA, IGF1R, PTEN, CCND1, which are relevant for ADG and/or T2DM, to be regulated by at least four out of 15 miRNAs [29,46,47,48]. As presented in Material S3, among all of the indicated targets, there were genes that were strongly involved in the regulation of cell cycle and apoptosis, and ECM remodeling, along with the signaling of insulin, MAPK, and TGF-β. We also noticed many genes directly associated with p53 signaling (CDKN1A, PPM1D, PTEN, DNMT1, KAT2B, BCL-2) [37,49]. The majority of the shared target genes were reported to be changed in AT upon diabetic milieu or during ADG, suggesting the role of selected miRNAs in the possibly simultaneous regulation of genes that are critical for Ads’ physiology and pathophysiology.

We also aimed to examine the potential biological relevance of miRNAs, which were significantly regulated by chronic HG in mature adipocytes (Material S1, Tables S7–10). Chronic treatment with HG is the most commonly used approach for studies on adipocytes in the context of T2DM. Moreover, mature adipocytes constitute the predominant type of cells in each adipose depot; thus, their response to HG might be considered an especially significant one. The obtained data suggested the involvement of these 11 miRNAs in the regulation of fatty acid and steroid biosynthesis, and molecular phenomena associated with cell cycle, hypertrophy, focal adhesion, and the signaling of cytokines, but appeared to diminish the relevance of signaling via TGF-β, MAPK, and more in comparison to previous analyses. Altogether, these results may further suggest that the impact of HG is relevant during the stages preceding the development of mature adipocytes, and thus is associated with ADG.

HG has been known to be especially detrimental for endothelial cells via inducing diabetic complications. These cells preserve “metabolic memory”—the normalization of glucose level does not remove their dysfunction—due to epigenetic modifications [7,8]. However, recent data suggests that the memorization of the effect of metabolic stressors is not only typical of endothelial cells. For instance, global miRNAs changes were also demonstrated as indicators of hyperglycemic memory in diabetic myocardium [50]. Muscle cells appear to memorize the effect of TNF-α exposure, physical activity, and more via using epigenetic mechanisms, which was called “epi-memory” [51]. Considering adipocytes, those treated with saturated fatty acids were suggested to express some kind of metabolic memory [52]. In the recent paper, Andersen et al. showed that visceral pAds from obese and diabetic patients are the subject of reprogramming, as they retain the “epi”-memory of donors in culture [53]. Consistently, our analysis revealed that a single HG stimulus applied at any culture stage triggered global miRNAs expression changes similar to those obtained upon chronic HG. Therefore, we speculate that visceral p/Ads may memorize the effect of HG via changes in miRNAs expression profile. We suggest that differentiated and mature Ads may particularly “remember” the effect of HG during the stage directly preceding their development. Despite obvious differences in study design, it is worth mentioning that Sun et al. also showed that mature 3T3L-1 adipocytes are responsive toward interchangeable treatment with normoglycemia and hyperglycemia [6]. Namely, the expression changes of inflammatory genes were time-dependent, and after three glycemic shifts, they showed an even greater magnitude than those obtained upon mere hyperglycemia. Moreover, although not presented herein, our team also performed BODIPY staining using time points and 14 culture variants (paper by Podolska et al. [54]). The obtained data suggested that while the morphology of the HHH cells was the most changed one in comparison to NNN, morphological changes concerning variants treated with intermittent HG were only to some extent reversible upon the normalization of glycemia. Herein, we suggest that our cell model, including groups with intermittent hyperglycemia, may, but only to some limited extent, reflect adipogenesis in conditions similar to those present in poorly controlled T2DM patients (blood glucose level fluctuations).

From another point of view, the phenomenon of memorization may also result in the introduction of detrimental epigenetic marks in pre-adipocytes or even cells at an earlier stage of differentiation (i.e., mesenchymal stem cells) of fetuses carried by mothers suffering from gestational diabetes. A recent study demonstrated AT-specific miR-483-3p programming in offspring in response to maternal diet along with having a devastating impact on ADG, leading to a decreased capacity of lipid accumulation and an increased risk of metabolic diseases [55]. The latter was also observed for adult offspring exposed to maternal diabetes, who showed miR-15a/b expression changes in skeletal muscles [56]. miR-101 was upregulated in endothelial fetal cells from diabetic mothers, implicating that numerous types of fetal cells respond to stimuli via miRNAs [57]. Undoubtedly, further studies are needed to elucidate whether Ads constitute the novel source of hyperglycemic memory and if so, which target genes regulated by the miRNAs are involved in this phenomenon as well.

On the contrary to our hypothesis, the surgical treatment of obesity or weight loss obtained due to a change of lifestyle are commonly known to ameliorate the symptoms of T2DM or even contribute to ending pharmacological treatment. Nevertheless, our study, as well as the above-mentioned studies, are suggesting that adipocytes may possess the memory of exposure to environmental factors. Therefore, we speculate that adipocytes may preserve the memory of exposure to hyperglycemia, but weight loss and the consequent normalization of glycemia may only contribute to slowing down the progression of metabolic and molecular imbalance in the course of diabetes and its complications. Indeed, this type of mechanism was clearly presented for endothelial cells [58]. Furthermore, it is worth considering the results of studies suggesting either the endothelial origin of white adipocytes or the existence of common progenitors for adipocytes and endothelial cells [59,60]. Regarding our results further, one should also take into account that we performed studies on a commercially available cell line (HPA-v) from only one donor (a 45-year-old Caucasian woman) with an unknown postnatal and prenatal history of exposure to metabolic stressors. Moreover, the differentiation protocol did not start from mesenchymal stem cells, whose pool is preserved much longer than that of committed pAds, which makes them particularly sensitive to various stimuli throughout life. Therefore, we believe that in order to obtain further support for the existence of metabolic memory in adipocytes, our study design should be implemented on adipose-derived mesenchymal stem cells from many donors, such as normoglycemic, insulin-resistant, prediabetic, and diabetic donors. It is also because this approach would need to take into account the variation of the level of insulin, as during the development of T2DM, compensatory hyperinsulinemia takes place, followed by hypoinsulinemia. We can only speculate that hyperglycemia together with hyperinsulinemia would elicit more pronounced morphological changes than those that were observed for our model by Podolska et al. [54], as insulin is a growth factor that is critical for the initiation and progression of adipogenesis. Interestingly, we found a reduction of miR-26b-5p in mature adipocytes treated chronically with HG (Figure 4 and Figure 5), while Xu et al. detected miR-26b-5p as downregulated in mature HPA-v adipocytes treated with high glucose and insulin [29].

Considering the weak and strong points of our experiment, it is to be highlighted that our study involved a three-stage differentiation of human visceral Ads, as the majority of current data originates from studies on two-stage ADG of 3T3-L1 mice cells. The 14 experimental groups also allowed for an in-depth analysis of the effect of chronic and intermittent HG on miRNAs, including an examination of the effect of memorization, which constitutes the major novelty of this study. Our experiment was not deprived of flaws, as we did not: (i) examine the biological relevance of significantly changed miRNAs using loss or gain-of-function techniques, nor (ii) validate any findings from bioinformatics analysis experimentally. 

## 5. Conclusions

miRNAs expression pattern changes upon physiological visceral ADG and HG modifies miRNAs expression at each stage of ADG. miRNAs appear to be molecules participating in the memorization of HG-dependent effect in pAds. At least 15 miRNAs that were sensitive to intermittent/chronic HG and changed upon ADG were identified. These molecules may be especially connected with the dysfunction of visceral Ads. To conclude, our findings suggest that miRNAs may be utilized by hyperglycemia to promote a diabetic phenotype, including its complications.

## Supplementary Materials

The following are available online at http://www.mdpi.com/2072-6643/10/11/1774/s1, Supplementary file contains: Materials S1 (Tables S1–S10, Figures S1–S5), Material S2 and Material S3.
