# Supplementary material for: Hyperglycemia Affects miRNAs Expression Pattern during Adipogenesis of Human Visceral Adipocytes—Is Memorization Involved?
Source: Nutrients. 2018 Nov 15;10(11):1774. doi: 10.3390/nu10111774 (PMC6266776; doi:10.3390/nu10111774)
Supplement: Supplementary file 1 [file nutrients-10-01774-s001.zip › nutrients-381697-SI/1-Material S2.docx]

**Material S2 : Discussion regarding 15 core miRNAs and miR-10a-5p**

miR-374b-5p was implied to directly regulate C/EBP-β expression during porcine ADG and in the context of lipid metabolism regulation [1, 2]. Moreover, DIANA miRpath v3 indicated miR-374b-5p to target genes of p53 signaling, including PTEN (Table S6). Physiologically, PI3K activation precedes Akt activation, which stimulates glucose uptake, cell growth, proliferation, and inhibits apoptosis. p53 is a great IR inducer, and suppresses PI3K/Akt signaling mainly due to the transcriptional activation of PTEN, which is the major repressor of PI3K/Akt [3]. It is tempting to speculate that p53 may modulate miR-374b-5p, which in turn may regulate Akt1 during response to DNA damage [4]. Although miR-374b-5p was earlier indicated as diabetes-related miRNA, we failed to find any data regarding its role in Ads/AT upon HG/diabetes, etc. [5].

Considering miR-26b-5p, it was revealed as a positive regulator of mice and human ADG during pre-adipocyte proliferation and differentiation due to cyclin D2 and PTEN targeting [6, 7]. In agreement with our data, miR-26b-5p decline was demonstrated in HPA-v Ads upon stimulation with HG, free fatty acids, growth hormone, and glucocorticoids, and simultaneously associated with PTEN upregulation [8, 9]. The same was observed for the VAT of insulin-resistant and obese subjects [9, 10].

By contrast, Yan et al. determined let-7g-5p as a repressor of intramuscular ADG along with being reduced in skeletal muscles of fetuses from obese pregnant ewes, which promoted fat deposition [11]. While our data indicated let-7g to be upregulated in chronically HG-treated differentiated Ads, let-7g was earlier reported to be downregulated in insulin-resistant 3T3-L1 Ads [12].

# The expression level of the next miRNA, miR-16-5p, was recently shown to raise during the ADG of hAD-MSC [13]. While we identified miR-16-5p to be upregulated in chronically HG-affected differentiated Ads, another research team identified its increase in the VAT of *ob/ob* and HFD-treated mice [14]. This raises doubts about the rightness of choice of miR-16-5p as an internal control gene for studies on visceral Ads and AT, as seen in many papers. Taking into account its involvement into fatty acid biosynthesis (Tab.1) and participation in the p53 genes network, further research regarding miR-16-5p is strongly warranted [15].

Another miRNA abundantly described in the context of ADG, miR-29a-3p, was demonstrated as an epigenetic regulator of lipoprotein lipase during mouse ADG [16]. Moreover, Zhu Y et al. showed miR-29a/b/c to affect 3T3-L1 ADG via DNMT3A targeting [17]. They found that miR-29a/b/c were downregulated during the contact inhibition (CI) stage, and the overexpression of miR-29a/b/c, especially during the CI stage, inhibited 3T3-L1 differentiation [17]. miR-29a-3p via CTRP6 targeting is an adipokine that is crucial for the differentiation of 3T3-L1 cells and inhibited intramuscular and subcutaneous adipocyte proliferation, but promoted differentiation by the mitogen-activated protein kinase (MAPK) signaling pathway [18]. miR-29a-3p is a well-known T2DM-related miRNA, which increases in AT and Ads upon diabetes and HG, being a regulator of insulin signaling [19, 20]. However, we found that it declined upon HG at the stage of pAds. Recently, some inconclusive results were brought to light, as miR-29a-3p was upregulated in AT after the weight loss of obese patients and in comparison to lean subjects [21, 22].

We detected miR-140-5p decline in differentiated Ads (NN versus N) along with its up and downregulation in mature Ads upon normoglycemia and HG, respectively (NNN versus NN/HHH versus HH). The first result was consistent with data provided by Qui et al., who observed an miR-140-5p decrease in 3T3-L1 Ads after seven days of differentiation [23]. miR-140-5p drop was also reported during human subcutaneous ADG at the seventh and 14^th^ differentiation day [24]. Oppositely, Xin et al. determined its gradual increase during 72 h after ADG induction [25]. Furthermore, they revealed a feedback loop where C/EBPs transactivates miR-140-5p, which in turn represses TGFBR1, so as to maintain the expression of C/EBPs and promote adipocyte differentiation [25]. DIANA showed miR-140-5p to target genes of the Hippo pathway, including TGFBR1 and TGFB1, which is crucial for ADG and responsive to liraglutide, an anti-diabetic drug (Tab.1) [26]. The miRNA also contributed to the commitment of pluripotent stem cells to the adipogenic lineage along with being indispensable for adipogenic capabilities of adipose-derived stem cells[27, 28]. In the current study, miR-140-5p was significantly decreased in chronically HG-treated pAds and mature Ads as well. Noteworthy, we also detected the trend toward its increase in differentiated Ads, suggesting its great responsiveness toward chronic HG. Inconclusively, miR-140-5 p was decreased in the SAT of diabetic patients and increased in the epididymal and inguinal AT of db/db mice [25, 29]. Moreover, Delic et al*.* revealed the magnitude of miR-140-5p downregulation to be a serum indicator of changes occurring during progression from pre-diabetes to late-stage diabetes in Zucker rats [30]. Contradictory results were obtained for diabetic subjects, where the reduction of serum miR-140-5p was shown after metformin treatment [31]. Taken together, the miRNA appears to be tightly connected with T2DM, AT, and ADG, making it a great candidate for metabolic diseases therapy.

# miR-31-3p was pronouncedly decreased in differentiated Ads and exhibited a tendency for the downregulation in mature ones. Interestingly, it was significantly reduced in mature Ads during ADG in HG. These results are in line with other reports, showing a substantial decline of miR-31-3p during ADG [24, 32, 33]. We also revealed miR-31-3p to be markedly decreased in mature Ads treated with HG during the differentiation process, and observed a weak trend for its downregulation upon chronic HG. This is not in agreement with recently published data, suggesting a miR-31-3p increase in both types of fat from T2DM patients [32].

Next, we observed a trend for a decline of miR-10a-5p during ADG in NG, yet HG appeared to further promote this effect. Consistently, Kang *et al.* demonstrated an miR-10a-5p decrease during the differentiation of 3T3-L1 [34, 35]. In the current study, miR-10a-5p showed a reduction in pAds treated with HG, although it did not reach statistical significance. This is in agreement with recent findings, suggesting a miR-10a-5p drop in pAds from chickens with different abdominal fatness [36]. Moreover, this miRNA showed a trend toward downregulation in differentiated Ads (HN versus NN), and it was markedly reduced in mature Ads treated once or chronically with HG. This may suggest the great impact of only one stimulus of HG and further imply Ads to ‘remember’ the effect of HG during the preceding stages of ADG. Nevertheless, miR-10a-5p was correlated with BMI and slightly upregulated in subcutaneous pAds and mature Ads isolated from obese versus lean subjects [24]. Interestingly, top records from DIANA depicted its association with fatty acids metabolism and biosynthesis (data not shown).

miR-151a-5 displayed very moderate changes during normoglycemic ADG, yet it exhibited a gradual downregulation during ADG in HG. Oppositely, Ortega et al*.* reported a slight miR-151a-5p increase in differentiating subcutaneous Ads [24], which may indicate differences between subcutaneous and visceral Ads. In our work, we also found miR-151a-5p to be differentially regulated by chronic and intermittent HG in differentiated and mature Ads. While AT-specific miR-151a-5p was earlier demonstrated to respond to weight loss and be reduced in high-thickened bovine backfat, there seems to be no other studies concerning its role upon diabetic milieu in AT [22, 37]. Noteworthy, its serum level was negatively correlated with body mass index (BMI), waist-to-hip ratio, insulin concentration, fasting glucose, HOMA-IR, and triglycerides [38]. DIANA associated its function with proteoglycans, making it even more interesting to become thoroughly examined in white Ads (Tab.1).

Our data suggested miR-106b-5p and miR-93-5p to be gradually increased during ADG in NG (ns), yet we found their significant and gradual downregulation only during ADG in HG. These miRNAs form an miR-17-92 cluster, whose several other components are accelerators of white ADG [39]. Wu et al. demonstrated both of these miRNAs to increase during brown ADG along with playing a role of adipogenic repressors [40]. In agreement with our data, miR-25-93-106b^–/–^ mice displayed IR along with the increased visceral adiposity due to the ADG promotion [41]. Authors have also highlighted the relevance of miR-93-5p in the context of negative regulation of Sirt-7 and Tbx3.

Additionally, miR-106b-5p was upregulated in differentiated Ads chronically treated with HG, yet we did not find any significant changes in mature Ads. Inconsistently, miR-106b-5p was raised in the high glucose-treated 3T3-L1 cells and AT of insulin-resistant patients, along with being positively correlated with IR [42]. According to Cioffi et al., the miRNA exhibited a trend toward downregulation in the VAT of *ob/ob* mice [41]. This, together with miR-106b-5p involvement in lipid metabolism via targeting ABCA-1, an AT-expressed gene critical for insulin sensitivity as well, makes additional research warranted [43, 44].

Considering miR-93-5p, we found it to be slightly upregulated in some variants of mature Ads. However, the most pronounced expression change was detected for NHN versus NNN, whose magnitude of reduction was similar to that observed upon chronic HG. This stays in agreement with previous findings showing miR-93-5p decline in visceral fat of ob/ob mice and high-thickened bovine backfat [37, 41]. In contrast, Wu et al*.* implied an miR-93-5p increase in the SAT of women with IR, which contributed to GLUT-4 downregulation, and its unchanged level upon either HG or insulin in 3T3-L1 Ads [42]. Moreover, weight loss evoked an miR-93-5p AT-specific reduction in relation to both obese and normal-weight patients [22]. Possibly, we suggest for the first time the downregulation of miR-93-3p in pAds treated with HG and during physiological visceral ADG. It should be also emphasized that miR-93-3p was indicated by DIANA to regulate genes participating in lipid metabolism (Tab.1).

We reported that miR-484 was physiologically declined in differentiated HPA-v Ads, yet we did not find any other data regarding miR-484 in the context of ADG. Considering the effect of HG, we only detected a reduction of miR-484 in pAds. Other studies showed a miR-484 decline in the SAT of obese without T2DM when compared to non-obese subjects, and in obese subjects without T2DM when compared with non-obese subjects [24]. DIANA mirpathv3 indicated miR-484 and miR-376c-3p to play a role in phenomena widely associated with lipid metabolism (Tab. 1). Noteworthy, the latter was predicted to target PTPLB, which is the differentially expressed gene in VAT from obese and lean mice and negatively correlated with BMI, total fat, and femoral fat pad mass [45]. In the current study, miR-376c-3p was decreased in differentiated and mature Ads in both glycemic conditions when comparing to pre-adipocytes, which is consistent with data obtained by Ortega et al. [24]. However, we did not detect significant changes of miR-376c-3p upon hyperglycemic stimulation at the particular culture stages. Earlier data suggested miR-376c-3p to be upregulated in mature subcutaneous Ads from obese individuals and in the VAT from obese subjects in comparison to SAT [24, 46].

Our data demonstrated the increase of miR-193b-3p in differentiated Ads (HH versus H) and its decrease in mature Ads (HHH versus HH) during HG-affected ADG. However, miR-193b-3p appeared to be gradually increased during physiological visceral ADG. This observation stays in agreement with the upregulation of miR-193b-3p reported by Knelengan et al. and Zaragosi et al. [13, 47]. Recently, miR-193b-3p was indicated as a promoter of ADG by targeting CRK-like proto-oncogene (CRKL) and focal adhesion kinase (FAK) along with being a stimulator of ROS signaling by targeting the antioxidant methionine sulfoxide reductase A [48, 49]. In the current study, miR-193b-3p exhibited the trend or expression change at all three culture stages, where its decline was suggested for H versus N, NHN versus NNN, and NHH versus NNH. Consistently, previous data implied an miR-193b-3p reduction in the SAT of obese patients along with promoting lipolysis and AT inflammation [50-52]. While miR-193b-3p overexpression reduced TNF-α and CCL2 production, it stimulated adiponectin expression due to indirect mechanisms, thus highlighting its association with IR [53]. White Ads’ TNF-α production was associated with expression changes of miR-193a-5p as well [50]. Circulating miR-193a-5p was reported to correlate with indexes of metabolic dysfunction along with abdominal and general obesity [54]. In our study, miR-193a-5p was gradually increased during physiological ADG, and exhibited either the tendency for or a significant decrease upon a particular single hit of HG in mature Ads (HNN versus NNN and NHN versus NNN). We also determined its upregulation in mature Ads while comparing HHN versus HNN, suggesting it to be sensitive to HG stimulation during a mere differentiation process as well. miR-193a-5p was earlier detected in VAT, yet, to our best knowledge, we are the first to show it differentially expressed in ADG and responsive toward HG [55, 56]. Interestingly, there is plenty of data showing changes of miR-193a-3p, but not -5p, upon diabetic or obesogenic milieu in AT [56].

References

1. Pan, S.; Zheng, Y.; Zhao, R.; Yang, X., miRNA-374 regulates dexamethasone-induced differentiation of primary cultures of porcine adipocytes. *Hormone and Metabolic Research* **2013,** *45* (07), 518-525.

2. Pan, S.; Zheng, Y.; Zhao, R.; Yang, X., MicroRNA-130b and microRNA-374b mediate the effect of maternal dietary protein on offspring lipid metabolism in Meishan pigs. *British Journal of Nutrition* **2013,** *109* (10), 1731-1738.

3. Strycharz, J.; Drzewoski, J.; Szemraj, J.; Sliwinska, A., Is p53 Involved in Tissue-Specific Insulin Resistance Formation? **2017,** *2017*, 9270549.

4. Gong, H.; Cao, Y.; Han, G.; Zhang, Y.; You, Q.; Wang, Y.; Pan, Y., p53/microRNA-374b/AKT1 regulates colorectal cancer cell apoptosis in response to DNA damage. *International journal of oncology* **2017,** *50* (5), 1785-1791.

5. Boles, A.; Kandimalla, R.; Reddy, P. H., Dynamics of Diabetes and Obesity: Epidemiological Perspective. *Biochimica et Biophysica Acta (BBA)-Molecular Basis of Disease* **2017**.

6. Xu, G.; Ji, C.; Song, G.; Shi, C.; Shen, Y.; Chen, L.; Yang, L.; Zhao, Y.; Guo, X., Obesity‑associated microRNA‑26b regulates the proliferation of human preadipocytes via arrest of the G1/S transition. *Molecular medicine reports* **2015,** *12* (3), 3648-3654.

7. Li, G.; Ning, C.; Ma, Y.; Jin, L.; Tang, Q.; Li, X.; Li, M.; Liu, H., miR-26b promotes 3T3-L1 adipocyte differentiation through targeting PTEN. *DNA and cell biology* **2017,** *36* (8), 672-681.

8. Xu, G.; Shi, C.; Ji, C.; Song, G.; Chen, L.; Yang, L.; Zhao, Y.; Guo, X., Expression of microRNA-26b, an obesity-related microRNA, is regulated by free fatty acids, glucose, dexamethasone and growth hormone in human adipocytes. *Molecular medicine reports* **2014,** *10* (1), 223-228.

9. Xu, G.; Ji, C.; Song, G.; Zhao, C.; Shi, C.; Song, L.; Chen, L.; Yang, L.; Huang, F.; Pang, L., MiR-26b modulates insulin sensitivity in adipocytes by interrupting the PTEN/PI3K/AKT pathway. *Int J Obes* **2015,** *39* (10), 1523.

10. Kirby, T. J.; Walton, R. G.; Finlin, B.; Zhu, B.; Unal, R.; Rasouli, N.; Peterson, C. A.; Kern, P. A., Integrative mRNA-microRNA analyses reveal novel interactions related to insulin sensitivity in human adipose tissue. *Physiological genomics* **2015,** *48* (2), 145-153.

11. Yan, X.; Huang, Y.; Zhao, J.-X.; Rogers, C. J.; Zhu, M.-J.; Ford, S. P.; Nathanielsz, P. W.; Du, M., Maternal obesity down-regulates microRNA (miRNA) let-7g expression, a possible mechanism for enhanced adipogenesis during ovine fetal skeletal muscle development. *International journal of obesity (2005)* **2013,** *37* (4), 568-575.

12. Ling, H. Y.; Ou, H. S.; Feng, S. D.; Zhang, X. Y.; Tuo, Q. H.; Chen, L. X.; Zhu, B. Y.; Gao, Z. P.; Tang, C. K.; Yin, W. D.; Zhang, L.; Liao, D. F., CHANGES IN microRNA (miR) profile and effects of miR-320 in insulin-resistant 3T3-L1 adipocytes. *Clinical and experimental pharmacology & physiology* **2009,** *36* (9), e32-9.

13. Knelangen, J. M.; van der Hoek, M. B.; Kong, W.-C.; Owens, J. A.; Fischer, B.; Santos, A. N., MicroRNA expression profile during adipogenic differentiation in mouse embryonic stem cells. *Physiological genomics* **2011,** *43* (10), 611-620.

14. Xie, H.; Sun, L.; Lodish, H. F., Targeting microRNAs in obesity. *Expert opinion on therapeutic targets* **2009,** *13* (10), 1227-1238.

15. Lezina, L.; Purmessur, N.; Antonov, A.; Ivanova, T.; Karpova, E.; Krishan, K.; Ivan, M.; Aksenova, V.; Tentler, D.; Garabadgiu, A., miR-16 and miR-26a target checkpoint kinases Wee1 and Chk1 in response to p53 activation by genotoxic stress. *Cell death & disease* **2013,** *4* (12), e953.

16. Bouvy-Liivrand, M.; Heinaniemi, M.; John, E.; Schneider, J. G.; Sauter, T.; Sinkkonen, L., Combinatorial regulation of lipoprotein lipase by microRNAs during mouse adipogenesis. *RNA biology* **2014,** *11* (1), 76-91.

17. Zhu, Y.; Zheng, G.; Wang, H.; Jia, Y.; Zhang, Y.; Tang, Y.; Li, W.; Fan, Y.; Zhang, X.; Liu, Y.; Liu, S., Downregulated miR-29a/b/c during Contact Inhibition Stage Promote 3T3-L1 Adipogenesis by Targeting DNMT3A. **2017,** *12* (1), e0170636.

18. Wu, W.; Zhang, J.; Zhao, C.; Sun, Y.; Pang, W.; Yang, G., CTRP6 Regulates Porcine Adipocyte Proliferation and Differentiation by the AdipoR1/MAPK Signaling Pathway. **2017,** *65* (27), 5512-5522.

19. He, A.; Zhu, L.; Gupta, N.; Chang, Y.; Fang, F., Overexpression of micro ribonucleic acid 29, highly up-regulated in diabetic rats, leads to insulin resistance in 3T3-L1 adipocytes. *Molecular endocrinology (Baltimore, Md.)* **2007,** *21* (11), 2785-94.

20. Herrera, B. M.; Lockstone, H. E.; Taylor, J. M.; Ria, M.; Barrett, A.; Collins, S.; Kaisaki, P.; Argoud, K.; Fernandez, C.; Travers, M. E.; Grew, J. P.; Randall, J. C.; Gloyn, A. L.; Gauguier, D.; McCarthy, M. I.; Lindgren, C. M., Global microRNA expression profiles in insulin target tissues in a spontaneous rat model of type 2 diabetes. *Diabetologia* **2010,** *53* (6), 1099-109.

21. Kristensen, M. M.; Davidsen, P. K.; Vigelso, A.; Hansen, C. N.; Jensen, L. J.; Jessen, N.; Bruun, J. M.; Dela, F.; Helge, J. W., miRNAs in human subcutaneous adipose tissue: Effects of weight loss induced by hypocaloric diet and exercise. *Obesity (Silver Spring, Md.)* **2017,** *25* (3), 572-580.

22. Kurylowicz, A.; Wicik, Z.; Owczarz, M.; Jonas, M. I.; Kotlarek, M.; Swierniak, M.; Lisik, W.; Jonas, M.; Noszczyk, B.; Puzianowska-Kuznicka, M., NGS Reveals Molecular Pathways Affected by Obesity and Weight Loss-Related Changes in miRNA Levels in Adipose Tissue. *International journal of molecular sciences* **2017,** *19* (1).

23. Qin, L.; Chen, Y.; Niu, Y.; Chen, W.; Wang, Q.; Xiao, S.; Li, A.; Xie, Y.; Li, J.; Zhao, X.; He, Z.; Mo, D., A deep investigation into the adipogenesis mechanism: profile of microRNAs regulating adipogenesis by modulating the canonical Wnt/beta-catenin signaling pathway. *BMC genomics* **2010,** *11*, 320.

24. Ortega, F. J.; Moreno-Navarrete, J. M.; Pardo, G.; Sabater, M.; Hummel, M.; Ferrer, A.; Rodriguez-Hermosa, J. I.; Ruiz, B.; Ricart, W.; Peral, B.; Fernandez-Real, J. M., MiRNA expression profile of human subcutaneous adipose and during adipocyte differentiation. *PLoS One* **2010,** *5* (2), e9022.

25. Zhang, X.; Chang, A.; Li, Y.; Gao, Y.; Wang, H.; Ma, Z.; Li, X.; Wang, B., miR-140-5p regulates adipocyte differentiation by targeting transforming growth factor-beta signaling. *Scientific reports* **2015,** *5*, 18118.

26. Li, Y.; Du, J.; Zhu, E.; Zhang, J.; Han, J.; Zhao, W.; Sun, B.; Tian, D., Liraglutide suppresses proliferation and induces adipogenic differentiation of 3T3-L1 cells via the Hippo-YAP signaling pathway. *Molecular medicine reports* **2018,** *17* (3), 4499-4507.

27. Liu, Y.; Zhang, Z. C.; Qian, S. W.; Zhang, Y. Y.; Huang, H. Y.; Tang, Y.; Guo, L.; Li, X.; Tang, Q. Q., MicroRNA-140 promotes adipocyte lineage commitment of C3H10T1/2 pluripotent stem cells via targeting osteopetrosis-associated transmembrane protein 1. *The Journal of biological chemistry* **2013,** *288* (12), 8222-30.

28. Gernapudi, R.; Wolfson, B.; Zhang, Y.; Yao, Y.; Yang, P.; Asahara, H.; Zhou, Q., MicroRNA 140 Promotes Expression of Long Noncoding RNA NEAT1 in Adipogenesis. *Molecular and cellular biology* **2016,** *36* (1), 30-8.

29. Kloting, N.; Berthold, S.; Kovacs, P.; Schon, M. R.; Fasshauer, M.; Ruschke, K.; Stumvoll, M.; Bluher, M., MicroRNA expression in human omental and subcutaneous adipose tissue. *PLoS One* **2009,** *4* (3), e4699.

30. Delic, D.; Eisele, C.; Schmid, R.; Luippold, G.; Mayoux, E.; Grempler, R., Characterization of Micro-RNA Changes during the Progression of Type 2 Diabetes in Zucker Diabetic Fatty Rats. *International journal of molecular sciences* **2016,** *17* (5).

31. Ortega, F. J.; Mercader, J. M.; Moreno-Navarrete, J. M.; Rovira, O.; Guerra, E.; Esteve, E.; Xifra, G.; Martinez, C.; Ricart, W.; Rieusset, J.; Rome, S.; Karczewska-Kupczewska, M.; Straczkowski, M.; Fernandez-Real, J. M., Profiling of circulating microRNAs reveals common microRNAs linked to type 2 diabetes that change with insulin sensitization. *Diabetes care* **2014,** *37* (5), 1375-83.

32. Gottmann, P.; Ouni, M.; Sausenthaler, S.; Roos, J.; Stirm, L.; Jähnert, M.; Kamitz, A.; Hallahan, N.; Jonas, W.; Fritsche, A., A computational biology approach of a genome-wide screen connected miRNAs to obesity and type 2 diabetes. *Molecular metabolism* **2018**.

33. Romao, J. M.; Guan, L. L., Chapter 21 - Adipogenesis and Obesity A2 - Sen, Chandan K. In *MicroRNA in Regenerative Medicine*, Academic Press: Oxford, 2015; pp 539-565.

34. Kang, M.; Yan, L. M.; Zhang, W. Y.; Li, Y. M.; Tang, A. Z.; Ou, H. S., Role of microRNA-21 in regulating 3T3-L1 adipocyte differentiation and adiponectin expression. *Molecular biology reports* **2013,** *40* (8), 5027-34.

35. Kang, M.; Yan, L. M.; Li, Y. M.; Zhang, W. Y.; Wang, H.; Tang, A. Z.; Ou, H. S., Inhibitory effect of microRNA-24 on fatty acid-binding protein expression on 3T3-L1 adipocyte differentiation. *Genetics and molecular research : GMR* **2013,** *12* (4), 5267-77.

36. Wang, W.; Du, Z. Q.; Cheng, B.; Wang, Y.; Yao, J.; Li, Y.; Cao, Z.; Luan, P.; Wang, N.; Li, H., Expression profiling of preadipocyte microRNAs by deep sequencing on chicken lines divergently selected for abdominal fatness. *PLoS One* **2015,** *10* (2), e0117843.

37. Jin, W.; Dodson, M. V.; Moore, S. S.; Basarab, J. A.; Guan, L. L., Characterization of microRNA expression in bovine adipose tissues: a potential regulatory mechanism of subcutaneous adipose tissue development. *BMC molecular biology* **2010,** *11*, 29.

38. Murri, M.; Insenser, M.; Fernandez-Duran, E.; San-Millan, J. L.; Luque-Ramirez, M.; Escobar-Morreale, H. F., Non-targeted profiling of circulating microRNAs in women with polycystic ovary syndrome (PCOS): effects of obesity and sex hormones. *Metabolism: clinical and experimental* **2018**.

39. Wang, Q.; Li, Y. C.; Wang, J.; Kong, J.; Qi, Y.; Quigg, R. J.; Li, X., miR-17-92 cluster accelerates adipocyte differentiation by negatively regulating tumor-suppressor Rb2/p130. *Proceedings of the National Academy of Sciences of the United States of America* **2008,** *105* (8), 2889-94.

40. Wu, Y.; Zuo, J.; Zhang, Y.; Xie, Y.; Hu, F.; Chen, L.; Liu, B.; Liu, F., Identification of miR-106b-93 as a negative regulator of brown adipocyte differentiation. *Biochemical and biophysical research communications* **2013,** *438* (4), 575-80.

41. Cioffi, M.; Vallespinos-Serrano, M.; Trabulo, S. M.; Fernandez-Marcos, P. J.; Firment, A. N.; Vazquez, B. N.; Vieira, C. R.; Mulero, F.; Camara, J. A.; Cronin, U. P.; Perez, M.; Soriano, J.; B, G. G.; Castells-Garcia, A.; Haage, V.; Raj, D.; Megias, D.; Hahn, S.; Serrano, L.; Moon, A.; Aicher, A.; Heeschen, C., MiR-93 Controls Adiposity via Inhibition of Sirt7 and Tbx3. *Cell reports* **2015,** *12* (10), 1594-605.

42. Wu, H. L.; Heneidi, S.; Chuang, T. Y.; Diamond, M. P.; Layman, L. C.; Azziz, R.; Chen, Y. H., The expression of the miR-25/93/106b family of micro-RNAs in the adipose tissue of women with polycystic ovary syndrome. *The Journal of clinical endocrinology and metabolism* **2014,** *99* (12), E2754-61.

43. Kim, J.; Yoon, H.; Ramirez, C. M.; Lee, S. M.; Hoe, H. S.; Fernandez-Hernando, C.; Kim, J., MiR-106b impairs cholesterol efflux and increases Abeta levels by repressing ABCA1 expression. *Experimental neurology* **2012,** *235* (2), 476-83.

44. de Haan, W.; Bhattacharjee, A.; Ruddle, P.; Kang, M. H.; Hayden, M. R., ABCA1 in adipocytes regulates adipose tissue lipid content, glucose tolerance, and insulin sensitivity. *Journal of lipid research* **2014,** *55* (3), 516-23.

45. Farahani, P.; Chiu, S.; Bowlus, C. L.; Boffelli, D.; Lee, E.; Fisler, J. S.; Krauss, R. M.; Warden, C. H., Obesity in BSB mice is correlated with expression of genes for iron homeostasis and leptin. *Obesity* **2004,** *12* (2), 191-204.

46. Kuryłowicz, A.; Wicik, Z.; Owczarz, M.; Jonas, M. I.; Kotlarek, M.; Świerniak, M.; Lisik, W.; Jonas, M.; Noszczyk, B.; Puzianowska-Kuźnicka, M., NGS Reveals Molecular Pathways Affected by Obesity and Weight Loss-Related Changes in miRNA Levels in Adipose Tissue. *International journal of molecular sciences* **2017,** *19* (1), 66.

47. Zaragosi, L.-E.; Wdziekonski, B.; Le Brigand, K.; Villageois, P.; Mari, B.; Waldmann, R.; Dani, C.; Barbry, P., Small RNA sequencing reveals miR-642a-3p as a novel adipocyte-specific microRNA and miR-30 as a key regulator of human adipogenesis. *Genome biology* **2011,** *12* (7), R64.

48. Mazzu, Y. Z.; Hu, Y.; Soni, R. K.; Mojica, K. M.; Qin, L.-X.; Agius, P.; Waxman, Z. M.; Mihailovic, A.; Socci, N. D.; Hendrickson, R. C., miR-193b–Regulated Signaling Networks Serve as Tumor Suppressors in Liposarcoma and Promote Adipogenesis in Adipose-Derived Stem Cells. *Cancer research* **2017,** *77* (21), 5728-5740.

49. Zaragosi, L. E.; Wdziekonski, B.; Brigand, K. L.; Villageois, P.; Mari, B.; Waldmann, R.; Dani, C.; Barbry, P., Small RNA sequencing reveals miR-642a-3p as a novel adipocyte-specific microRNA and miR-30 as a key regulator of human adipogenesis. *Genome biology* **2011,** *12* (7), R64.

50. Lorente-Cebrián, S.; Mejhert, N.; Kulyté, A.; Laurencikiene, J.; Åström, G.; Hedén, P.; Rydén, M.; Arner, P., MicroRNAs regulate human adipocyte lipolysis: effects of miR-145 are linked to TNF-α. *PloS one* **2014,** *9* (1), e86800.

51. Ortega, F. J.; Moreno, M.; Mercader, J. M.; Moreno-Navarrete, J. M.; Fuentes-Batllevell, N.; Sabater, M.; Ricart, W.; Fernández-Real, J. M., Inflammation triggers specific microRNA profiles in human adipocytes and macrophages and in their supernatants. *Clinical epigenetics* **2015,** *7* (1), 49.

52. Arner, E.; Mejhert, N.; Kulyté, A.; Balwierz, P. J.; Pachkov, M.; Cormont, M.; Lorente-Cebrián, S.; Ehrlund, A.; Laurencikiene, J.; Hedén, P., Adipose tissue microRNAs as regulators of CCL2 production in human obesity. *Diabetes* **2012,** *61* (8), 1986-1993.

53. Belarbi, Y.; Mejhert, N.; Lorente-Cebrián, S.; Dahlman, I.; Arner, P.; Rydén, M.; Kulyté, A., MicroRNA-193b controls adiponectin production in human white adipose tissue. *The Journal of Clinical Endocrinology & Metabolism* **2015,** *100* (8), E1084-E1088.

54. Murri, M.; Insenser, M.; Fernández-Durán, E.; San-Millán, J. L.; Luque-Ramírez, M.; Escobar-Morreale, H. F., Non-targeted profiling of circulating microRNAs in women with polycystic ovary syndrome (PCOS): effects of obesity and sex hormones. *Metabolism: clinical and experimental* **2018**.

55. Estep, M.; Armistead, D.; Hossain, N.; Elarainy, H.; Goodman, Z.; Baranova, A.; Chandhoke, V.; Younossi, Z., Differential expression of miRNAs in the visceral adipose tissue of patients with non‐alcoholic fatty liver disease. *Alimentary pharmacology & therapeutics* **2010,** *32* (3), 487-497.

56. Ocłoń, E.; Latacz, A.; Zubel–Łojek, J.; Pierzchała–Koziec, K., Hyperglycemia-induced changes in miRNA expression patterns in epicardial adipose tissue of piglets. *Journal of Endocrinology* **2016,** *229* (3), 259-266.
