# Supplementary material for: Hyperglycemia Affects miRNAs Expression Pattern during Adipogenesis of Human Visceral Adipocytes—Is Memorization Involved?
Source: Nutrients. 2018 Nov 15;10(11):1774. doi: 10.3390/nu10111774 (PMC6266776; doi:10.3390/nu10111774)
Supplement: Supplementary file 1 [file nutrients-10-01774-s001.zip › nutrients-381697-SI/1-Material S3.docx]

| Major function | GENE | Participation in adipogenesis (ADG) | | Effect in AT in response to HG/T2DM/obesity/HFD/anti-diabetic drugs | Ref. |
| --- | --- | --- | --- | --- | --- |
| Cell cycle progression and  checkpoints control | CCND1 | - participate in MCE and cell cycle arrest  -let-7-regulated | -suppresses PPARγ, C/EBPα | - | [1, 2] |
|  | CCND2 |  | -regulated by mir-26b | - | [1-3] |
|  | CCNE1 |  | -regulated by miR-26a during ADG of AF-MSC | - | [2, 4] |
|  | CCNT2 | -differently localized during stages of ADG | | - | [5] |
|  | CDK6 | -phosphorylates PPARγ with CCND3  -could be modulated by miR-33b  -regulated by miR-26a during ADG of AF-MSC | | - | [4, 6, 7] |
|  | E2F1 | -induces PPARγ during MCE  -enhances C/EBPα expression | | -raised in VAT of HFD-treated and db/db mice and obese subjects -defines a dysmetabolic subtype of AT  -sensitizes autophagy of VAT to inflammatory stimuli | [8-10] |
|  | RB1 | -increases during ADG, critical for keeping adipogenic characteristics in mature adipocytes  -proadipogenic, modulated by PI3K and mTOR  -interacts with PPARγ, C/EBPs, regulates PGC-1α | | -mice RB1 haploinsufficiency implied to protect against obesity and IR  -declined in AT of obese patients and animals  -negatively correlated with BMI and IR | [11-14] |
|  | WEE1 | - | | - |  |
|  | CDKN1A | -takes part in MCE and cell-cycle arrest of ADG  -regulated by PPARγ | | -increased with p53 in AT upon diabetic milieu, mediates hyperplasia and hypertrophy of adipocytes, induces senescence and inflammation | [15, 16] |
|  | PPM1D | Proadipogenic via dephosphorylating PPARγ | | -its deficiency in mice causes inflammation and IR with or without HFD | [17, 18] |
| DNA replication | PURA | - | | -differentially expressed in AT from patients with and without IR | [19, 20] |
| Autophagy | ATG16L1 | - | | - |  |
| ECM remodeling | MMP-2 | -increased during ADG | | -increased in serum, SAT, and epicardial AT of obese patients and mice | [21, 22] |
|  | RECK | -determines adipogenic or osteogenic hMSC differentiation | | - | [23] |
|  | LAMC1 | -increases during ADG | | - | [24] |
| Angiogenesis and ECM remodeling | VEGFA | -increases during ADG  -promotes adipogenesis of hMSC while being regulated by miR-128 | | -its upregulation and downregulation implied to ameliorate obesity, IR, and inflammation  -key inducer of angiogenesis in WAT, stimulates beiging of WAT | [25-30] |
| TGF-β signaling | TGFBR1 | -regulated by miR-140-5p, represses ADG  -indirectly changed by miR-181a-5p during ADG | | -decreased after amelioration of visceral adiposity and IR in mice  -increased by treatment with rosiglitazone in SAT of diabetic rats | [31-34] |
|  | SMAD7 | -directly regulated by miR-181a-5p during ADG | | -increased by treatment with rosiglitazone in SAT of diabetic rats | [31, 34] |
| Suppression of apoptosis | MCL1 | -participates in brown ADG | | -detected in omental AT of massively obese men | [35, 36] |
|  | BCL2 | -increased in response to IL-6-stimulated pre-adipocytes proliferation | | -its overexpression blocks troglitazone‐stimulated adipocyte apoptosis | [37, 38] |
| Histone and non-histone acetylation | KAT2B | -controls 3T3-L1 pre-adipocytes proliferation as well as differentiation (acetylation of C/EBPβ) | | - | [39, 40] |
| Regulation of methylation | ZBTB4 | - | | - |  |
|  | DNMT1 | -regulates the timing of ADG along with methylation pattern of DNA and histones  -negatively regulated by ATP-citrate lyase, miR-148a-5p and collagen XV  -controls a level of genes that is critical for ADG and AT | | -hypermethylates promoter of adiponectin, thus acting as an inducer of IR in adipocytes of obese mice  -positively correlated with BMI | [41-43] |
| Chromatin remodeling | BMI1 | -its inactivation in mice MSC slows down ADG | | - | [44] |
|  | HMGA2 | -changed during ADG, promotes it by stimulating the C/EBPβ-mediated regulation of PPARγ  -could be modulated by miR-33b  -regulated by let-7 during ADG | | -elevated in WAT of obese and diabetic patients  -HMGA2-deficient mice exhibit resistance to HFD-induced obesity and markedly reduced fat mass | [2, 7, 45, 46] |
| Insulin signaling | AKT2 | -positive and key regulator of ADG, including visceral adipose precursor proliferation  -directly interacts with Sirt1 and C/EBPα | | -reduced in AT and inversely correlated with BMI of obese patients  -critical for insulin signaling/glucose uptake/IR in adipocytes  -via interacting with Sirt1 inhibits inflammation in AT upon HFD in mice | [30, 47-49] |
|  | IGFR1 | -mice with fat-specific KO of IGF1R and IR a have decreased rate of white and brown ADG | | -mice with fat-specific KO of IGF1R and IR are cold-sensitive, exhibit low fat mass and resistance to age and diet-induced obesity | [50] |
|  | PTEN | -reduced by miR-148a-5p, thus inhibiting rabbit pre-adipocyte differentiation  -regulated during ADG induction in MSC  -regulated by miR-26a during ADG of AF-MSC | | -upregulated upon diabetic milieu  -one of the most well-known inducers of IR not only in adipocytes  -controlled by miR-26b and p53 to suppress PI3K/AKT signaling | [4, 51-53] |
| MAPK signaling | MAPK9 | -constitutively activated p46JNK2 is necessary for the induction C/EBPδ during the initial stage of adipogenic differentiation | | -fat-specific JNK2 activation’s suppression decreases inflammation and ameliorates HFD-induced obesity  -JNK signaling is crucial IR due to participation in insulin signaling axis | [54, 55] |
| Signaling via small G-proteins | KRAS | - | | - |  |
|  | ARHGDIA | -changed during ADG | | - | [56] |
| Inflammation | PTGS2 | -inconclusively, suggested to exert a suppressive and stimulatory effect on adipocyte differentiation | | -participates in hypoxia and hypertrophy of adipocytes *in vitro*  -stimulates inflammation/IR in obese animals and human subjects  -positively correlated with human BMI | [57, 58] |
| Stress response | CHORDC1 | - | | - |  |

**Material S3**. Summary of findings regarding contribution to ADG and AT-related changes evoked/associated with HG/obesity/diabetes etc., which were revealed for target genes indicated by miRTargetLink Human for 15 core miRNAs. All of the target genes were assigned to groups reflecting their major biological functions. HFD- high fat diet, AF-MSC – amniotic fluid mesenchymal stem cells, MCE – mitotic clonal expansion, hMSC – human mesenchymal stem cells, KO – knockout.

**References**

1. Hishida, T.; Naito, K.; Osada, S.; Nishizuka, M.; Imagawa, M., Crucial roles of D-type cyclins in the early stage of adipocyte differentiation. *Biochemical and biophysical research communications* **2008,** *370* (2), 289-94.

2. Sun, T.; Fu, M.; Bookout, A. L.; Kliewer, S. A.; Mangelsdorf, D. J., MicroRNA let-7 regulates 3T3-L1 adipogenesis. *Molecular endocrinology (Baltimore, Md.)* **2009,** *23* (6), 925-31.

3. Xu, G.; Ji, C.; Song, G.; Shi, C.; Shen, Y.; Chen, L.; Yang, L.; Zhao, Y.; Guo, X., Obesity‑associated microRNA‑26b regulates the proliferation of human preadipocytes via arrest of the G1/S transition. *Molecular medicine reports* **2015,** *12* (3), 3648-3654.

4. Trohatou, O.; Zagoura, D.; Orfanos, N. K.; Pappa, K. I.; Marinos, E.; Anagnou, N. P.; Roubelakis, M. G., miR-26a Mediates Adipogenesis of Amniotic Fluid Mesenchymal Stem/Stromal Cells via PTEN, Cyclin E1, and CDK6. *Stem cells and development* **2017,** *26* (7), 482-494.

5. Iankova, I.; Petersen, R. K.; Annicotte, J.-S.; Chavey, C.; Hansen, J. B.; Kratchmarova, I.; Sarruf, D.; Benkirane, M.; Kristiansen, K.; Fajas, L., Peroxisome proliferator-activated receptor γ recruits the positive transcription elongation factor b complex to activate transcription and promote adipogenesis. *Molecular endocrinology* **2006,** *20* (7), 1494-1505.

6. Sarruf, D. A.; Iankova, I.; Abella, A.; Assou, S.; Miard, S.; Fajas, L., Cyclin D3 promotes adipogenesis through activation of peroxisome proliferator-activated receptor gamma. *Molecular and cellular biology* **2005,** *25* (22), 9985-95.

7. Price, N. L.; Holtrup, B.; Kwei, S. L.; Wabitsch, M.; Rodeheffer, M.; Bianchini, L.; Suarez, Y.; Fernandez-Hernando, C., SREBP-1c/MicroRNA 33b Genomic Loci Control Adipocyte Differentiation. *Molecular and cellular biology* **2016,** *36* (7), 1180-93.

8. Haim, Y.; Bluher, M.; Konrad, D.; Goldstein, N.; Kloting, N.; Harman-Boehm, I.; Kirshtein, B.; Ginsberg, D.; Tarnovscki, T.; Gepner, Y.; Shai, I.; Rudich, A., ASK1 (MAP3K5) is transcriptionally upregulated by E2F1 in adipose tissue in obesity, molecularly defining a human dys-metabolic obese phenotype. *Molecular metabolism* **2017,** *6* (7), 725-736.

9. Choi, Y.; Jang, S.; Choi, M. S.; Ryoo, Z. Y.; Park, T., Increased expression of FGF1-mediated signaling molecules in adipose tissue of obese mice. *Journal of physiology and biochemistry* **2016,** *72* (2), 157-67.

10. Fajas, L.; Landsberg, R. L.; Huss-Garcia, Y.; Sardet, C.; Lees, J. A.; Auwerx, J., E2Fs regulate adipocyte differentiation. *Developmental cell* **2002,** *3* (1), 39-49.

11. Fajas, L.; Egler, V.; Reiter, R.; Hansen, J.; Kristiansen, K.; Debril, M. B.; Miard, S.; Auwerx, J., The retinoblastoma-histone deacetylase 3 complex inhibits PPARgamma and adipocyte differentiation. *Developmental cell* **2002,** *3* (6), 903-10.

12. Usui, I.; Haruta, T.; Iwata, M.; Takano, A.; Uno, T.; Kawahara, J.; Ueno, E.; Sasaoka, T.; Kobayashi, M., Retinoblastoma protein phosphorylation via PI 3-kinase and mTOR pathway regulates adipocyte differentiation. *Biochemical and biophysical research communications* **2000,** *275* (1), 115-20.

13. Ribot, J.; Oliver, P.; Serra, F.; Palou, A., Retinoic acid modulates the retinoblastoma protein during adipocyte terminal differentiation. *Biochimica et biophysica acta* **2005,** *1740* (2), 249-57.

14. Chu, D. T.; Tao, Y., Molecular connections of obesity and aging: a focus on adipose protein 53 and retinoblastoma protein. *Biogerontology* **2017,** *18* (3), 321-332.

15. Morrison, R. F.; Farmer, S. R., Role of PPARgamma in regulating a cascade expression of cyclin-dependent kinase inhibitors, p18(INK4c) and p21(Waf1/Cip1), during adipogenesis. *The Journal of biological chemistry* **1999,** *274* (24), 17088-97.

16. Strycharz, J.; Drzewoski, J.; Szemraj, J.; Sliwinska, A., Is p53 Involved in Tissue-Specific Insulin Resistance Formation? **2017,** *2017*, 9270549.

17. Li, D.; Zhang, L.; Xu, L.; Liu, L.; He, Y.; Zhang, Y.; Huang, X.; Zhao, T.; Wu, L.; Zhao, Y.; Wu, K.; Li, H.; Yu, X.; Zhao, T.; Gong, S.; Fan, M.; Zhu, L., WIP1 phosphatase is a critical regulator of adipogenesis through dephosphorylating PPARgamma serine 112. *Cellular and molecular life sciences : CMLS* **2017,** *74* (11), 2067-2079.

18. Armata, H. L.; Chamberland, S.; Watts, L.; Ko, H. J.; Lee, Y.; Jung, D. Y.; Kim, J. K.; Sluss, H. K., Deficiency of the tumor promoter gene wip1 induces insulin resistance. *Molecular endocrinology (Baltimore, Md.)* **2015,** *29* (1), 28-39.

19. Zhang, L.; Cui, Y.; Fu, F.; Li, Z.; Pan, X.; Li, H.; Li, L., An insight into the key genes and biological functions associated with insulin resistance in adipose tissue with microarray technology. *Molecular medicine reports* **2015,** *11* (3), 1963-1967.

20. Dreja, T.; Jovanovic, Z.; Rasche, A.; Kluge, R.; Herwig, R.; Tung, Y.; Joost, H.; Yeo, G.; Al-Hasani, H., Diet-induced gene expression of isolated pancreatic islets from a polygenic mouse model of the metabolic syndrome. *Diabetologia* **2010,** *53* (2), 309.

21. Kim, W. K.; Kang, N. E.; Kim, M. H.; Ha, A. W., Peanut sprout ethanol extract inhibits the adipocyte proliferation, differentiation, and matrix metalloproteinases activities in mouse fibroblast 3T3-L1 preadipocytes. *Nutrition research and practice* **2013,** *7* (3), 160-5.

22. Caria, C.; Gotardo, E. M. F.; Santos, P. S.; Acedo, S. C.; de Morais, T. R.; Ribeiro, M. L.; Gambero, A., Extracellular matrix remodeling and matrix metalloproteinase inhibition in visceral adipose during weight cycling in mice. *Experimental cell research* **2017,** *359* (2), 431-440.

23. Mahl, C.; Egea, V.; Megens, R. T.; Pitsch, T.; Santovito, D.; Weber, C.; Ries, C., RECK (reversion-inducing cysteine-rich protein with Kazal motifs) regulates migration, differentiation and Wnt/beta-catenin signaling in human mesenchymal stem cells. *Cellular and molecular life sciences : CMLS* **2016,** *73* (7), 1489-501.

24. Niimi, T.; Kumagai, C.; Okano, M.; Kitagawa, Y., Differentiation-dependent expression of laminin-8 (alpha 4 beta 1 gamma 1) mRNAs in mouse 3T3-L1 adipocytes. *Matrix biology : journal of the International Society for Matrix Biology* **1997,** *16* (4), 223-30.

25. Fukumura, D.; Ushiyama, A.; Duda, D. G.; Xu, L.; Tam, J.; Krishna, V.; Chatterjee, K.; Garkavtsev, I.; Jain, R. K., Paracrine regulation of angiogenesis and adipocyte differentiation during in vivo adipogenesis. *Circulation research* **2003,** *93* (9), e88-97.

26. Zhang, W.; Yao, C.; Wei, Z.; Dong, Q., miR-128 promoted adipogenic differentiation and inhibited osteogenic differentiation of human mesenchymal stem cells by suppression of VEGF pathway. *Journal of receptor and signal transduction research* **2017,** *37* (3), 217-223.

27. Park, J.; Kim, M.; Sun, K.; An, Y. A.; Gu, X.; Scherer, P. E., VEGF-A-Expressing Adipose Tissue Shows Rapid Beiging and Enhanced Survival After Transplantation and Confers IL-4-Independent Metabolic Improvements. *Diabetes* **2017,** *66* (6), 1479-1490.

28. Lu, X.; Ji, Y.; Zhang, L.; Zhang, Y.; Zhang, S.; An, Y.; Liu, P.; Zheng, Y., Resistance to obesity by repression of VEGF gene expression through induction of brown-like adipocyte differentiation. *Endocrinology* **2012,** *153* (7), 3123-32.

29. Sung, H. K.; Doh, K. O.; Son, J. E.; Park, J. G.; Bae, Y.; Choi, S.; Nelson, S. M.; Cowling, R.; Nagy, K.; Michael, I. P.; Koh, G. Y.; Adamson, S. L.; Pawson, T.; Nagy, A., Adipose vascular endothelial growth factor regulates metabolic homeostasis through angiogenesis. *Cell metabolism* **2013,** *17* (1), 61-72.

30. Matulewicz, N.; Stefanowicz, M.; Nikołajuk, A.; Karczewska-Kupczewska, M., Markers of Adipogenesis, but Not Inflammation, in Adipose Tissue Are Independently Related to Insulin Sensitivity. *The Journal of Clinical Endocrinology & Metabolism* **2017,** *102* (8), 3040-3049.

31. Beaudoin, M. S.; Snook, L. A.; Arkell, A. M.; Stefanson, A.; Wan, Z.; Simpson, J. A.; Holloway, G. P.; Wright, D. C., Novel effects of rosiglitazone on SMAD2 and SMAD3 signaling in white adipose tissue of diabetic rats. *Obesity (Silver Spring, Md.)* **2014,** *22* (7), 1632-42.

32. Zhang, X.; Chang, A.; Li, Y.; Gao, Y.; Wang, H.; Ma, Z.; Li, X.; Wang, B., miR-140-5p regulates adipocyte differentiation by targeting transforming growth factor-beta signaling. *Scientific reports* **2015,** *5*, 18118.

33. Seo, K.-H.; Kim, H.; Chon, J.-W.; Kim, D.-H.; Nah, S.-Y.; Arvik, T.; Yokoyama, W., Flavonoid-rich Chardonnay grape seed flour supplementation ameliorates diet-induced visceral adiposity, insulin resistance, and glucose intolerance via altered adipose tissue gene expression. *Journal of Functional Foods* **2015,** *17*, 881-891.

34. Ouyang, D.; Xu, L.; Zhang, L.; Guo, D.; Tan, X.; Yu, X.; Qi, J.; Ye, Y.; Liu, Q.; Ma, Y.; Li, Y., MiR-181a-5p regulates 3T3-L1 cell adipogenesis by targeting Smad7 and Tcf7l2. *Acta biochimica et biophysica Sinica* **2016,** *48* (11), 1034-1041.

35. Miranda, S.; Gonzalez-Rodriguez, A.; Revuelta-Cervantes, J.; Rondinone, C. M.; Valverde, A. M., Beneficial effects of PTP1B deficiency on brown adipocyte differentiation and protection against apoptosis induced by pro- and anti-inflammatory stimuli. *Cellular signalling* **2010,** *22* (4), 645-59.

36. Zhang, Y.; Bosse, Y.; Marceau, P.; Biron, S.; Lebel, S.; Richard, D.; Vohl, M. C.; Tchernof, A., Gene expression variability in subcutaneous and omental adipose tissue of obese men. *Gene expression* **2007,** *14* (1), 35-46.

37. Gustafson, B.; Smith, U., Cytokines promote Wnt signaling and inflammation and impair the normal differentiation and lipid accumulation in 3T3-L1 preadipocytes. *The Journal of biological chemistry* **2006,** *281* (14), 9507-16.

38. Xiao, Y.; Yuan, T.; Yao, W.; Liao, K., 3T3-L1 adipocyte apoptosis induced by thiazolidinediones is peroxisome proliferator-activated receptor-gamma-dependent and mediated by the caspase-3-dependent apoptotic pathway. *The FEBS journal* **2010,** *277* (3), 687-96.

39. Wiper-Bergeron, N.; Salem, H. A.; Tomlinson, J. J.; Wu, D.; Hache, R. J., Glucocorticoid-stimulated preadipocyte differentiation is mediated through acetylation of C/EBPbeta by GCN5. *Proceedings of the National Academy of Sciences of the United States of America* **2007,** *104* (8), 2703-8.

40. Gupta, P.; Park, S. W.; Farooqui, M.; Wei, L. N., Orphan nuclear receptor TR2, a mediator of preadipocyte proliferation, is differentially regulated by RA through exchange of coactivator PCAF with corepressor RIP140 on a platform molecule GRIP1. *Nucleic acids research* **2007,** *35* (7), 2269-82.

41. Liu, G.; Li, M.; Xu, Y.; Wu, S.; Saeed, M.; Sun, C., ColXV promotes adipocyte differentiation via inhibiting DNA methylation and cAMP/PKA pathway in mice. *Oncotarget* **2017,** *8* (36), 60135-60148.

42. Kim, A. Y.; Park, Y. J.; Pan, X.; Shin, K. C.; Kwak, S. H.; Bassas, A. F.; Sallam, R. M., Obesity-induced DNA hypermethylation of the adiponectin gene mediates insulin resistance. **2015,** *6*, 7585.

43. Yuan, Y.; Liu, C.; Wan, D.; Huang, K.; Zheng, L., DNA/Histone Methylation and Adipocyte Differentiation: Applications to Obesity. *Handbook of Nutrition, Diet, and Epigenetics* **2017**, 1-18.

44. Petrov, N.; Vereschagina, N.; Sushilova, E.; Kropotov, A.; Miheeva, N.; Popov, B., A product of BMI1 gene, a key component of the Polycomb family, positively regulates adipocyte differentiation of mouse mesenchymal stem cells. *Cell and Tissue Biology* **2016,** *10* (3), 171-177.

45. Xi, Y.; Shen, W.; Ma, L.; Zhao, M.; Zheng, J.; Bu, S.; Hino, S.; Nakao, M., HMGA2 promotes adipogenesis by activating C/EBPbeta-mediated expression of PPARgamma. *Biochemical and biophysical research communications* **2016,** *472* (4), 617-23.

46. Markowski, D. N.; Thies, H. W.; Gottlieb, A.; Wenk, H.; Wischnewsky, M.; Bullerdiek, J., HMGA2 expression in white adipose tissue linking cellular senescence with diabetes. *Genes & nutrition* **2013,** *8* (5), 449-56.

47. Wagner, G.; Lindroos-Christensen, J.; Einwallner, E.; Husa, J.; Zapf, T. C.; Lipp, K.; Rauscher, S.; Groger, M.; Spittler, A.; Loewe, R.; Gruber, F.; Duvigneau, J. C.; Mohr, T.; Sutterluty-Fall, H.; Klinglmuller, F.; Prager, G.; Huppertz, B.; Yun, J.; Wagner, O.; Esterbauer, H.; Bilban, M., HO-1 inhibits preadipocyte proliferation and differentiation at the onset of obesity via ROS dependent activation of Akt2. *Scientific reports* **2017,** *7*, 40881.

48. Zhang, Y.; Xie, L.; Gunasekar, S. K.; Tong, D.; Mishra, A.; Gibson, W. J.; Wang, C.; Fidler, T.; Marthaler, B.; Klingelhutz, A.; Abel, E. D.; Samuel, I.; Smith, J. K.; Cao, L.; Sah, R., Erratum: SWELL1 is a regulator of adipocyte size, insulin signalling and glucose homeostasis. *Nature cell biology* **2017,** *19* (7), 873.

49. Liu, Z.; Gan, L.; Liu, G.; Chen, Y.; Wu, T.; Feng, F.; Sun, C., Sirt1 decreased adipose inflammation by interacting with Akt2 and inhibiting mTOR/S6K1 pathway in mice. *Journal of lipid research* **2016,** *57* (8), 1373-1381.

50. Boucher, J.; Mori, M. A.; Lee, K. Y.; Smyth, G.; Liew, C. W.; Macotela, Y.; Rourk, M.; Bluher, M.; Russell, S. J.; Kahn, C. R., Impaired thermogenesis and adipose tissue development in mice with fat-specific disruption of insulin and IGF-1 signalling. *Nature communications* **2012,** *3*, 902.

51. He, H.; Cai, M.; Zhu, J.; Xiao, W.; Liu, B.; Shi, Y.; Yang, X.; Liang, X.; Zheng, T.; Hu, S.; Jia, X.; Chen, S.; Wang, J.; Qin, Y.; Lai, S., miR-148a-3p promotes rabbit preadipocyte differentiation by targeting PTEN. *In vitro cellular & developmental biology. Animal* **2018,** *54* (3), 241-249.

52. Xu, G.; Ji, C.; Song, G.; Zhao, C.; Shi, C.; Song, L.; Chen, L.; Yang, L.; Huang, F.; Pang, L.; Zhang, N.; Zhao, Y.; Guo, X., MiR-26b modulates insulin sensitivity in adipocytes by interrupting the PTEN/PI3K/AKT pathway. *International journal of obesity (2005)* **2015,** *39* (10), 1523-30.

53. Song, B. Q.; Chi, Y.; Li, X.; Du, W. J.; Han, Z. B.; Tian, J. J.; Li, J. J.; Chen, F.; Wu, H. H.; Han, L. X.; Lu, S. H.; Zheng, Y. Z.; Han, Z. C., Inhibition of Notch Signaling Promotes the Adipogenic Differentiation of Mesenchymal Stem Cells Through Autophagy Activation and PTEN-PI3K/AKT/mTOR Pathway. *Cellular physiology and biochemistry : international journal of experimental cellular physiology, biochemistry, and pharmacology* **2015,** *36* (5), 1991-2002.

54. Kusuyama, J.; Ohnishi, T.; Bandow, K.; Amir, M. S.; Shima, K.; Semba, I.; Matsuguchi, T., Constitutive activation of p46JNK2 is indispensable for C/EBPdelta induction in the initial stage of adipogenic differentiation. *The Biochemical journal* **2017,** *474* (20), 3421-3437.

55. Zhang, X.; Xu, A.; Chung, S. K.; Cresser, J. H.; Sweeney, G.; Wong, R. L.; Lin, A.; Lam, K. S., Selective inactivation of c-Jun NH2-terminal kinase in adipose tissue protects against diet-induced obesity and improves insulin sensitivity in both liver and skeletal muscle in mice. *Diabetes* **2011,** *60* (2), 486-95.

56. Welsh, G. I.; Griffiths, M. R.; Webster, K. J.; Page, M. J.; Tavare, J. M., Proteome analysis of adipogenesis. *Proteomics* **2004,** *4* (4), 1042-51.

57. Ghoshal, S.; Trivedi, D. B.; Graf, G. A.; Loftin, C. D., Cyclooxygenase-2 deficiency attenuates adipose tissue differentiation and inflammation in mice. *The Journal of biological chemistry* **2011,** *286* (1), 889-98.

58. Chan, P. C.; Hsiao, F. C.; Chang, H. M.; Wabitsch, M.; Hsieh, P. S., Importance of adipocyte cyclooxygenase-2 and prostaglandin E2-prostaglandin E receptor 3 signaling in the development of obesity-induced adipose tissue inflammation and insulin resistance. *FASEB journal : official publication of the Federation of American Societies for Experimental Biology* **2016,** *30* (6), 2282-97.
