# Supplementary material for: Hyperglycemia Affects miRNAs Expression Pattern during Adipogenesis of Human Visceral Adipocytes—Is Memorization Involved?
Source: Nutrients. 2018 Nov 15;10(11):1774. doi: 10.3390/nu10111774 (PMC6266776; doi:10.3390/nu10111774)
Supplement: Supplementary file 1 [file nutrients-10-01774-s001.zip › nutrients-381697-SI/1-Material-S1.docx]

**Material S1 contains: Table S1–S10 and Figures S1–S5 with description.**

**Table S1**. A full list of miRBase and assay IDs of the examined molecules (miRNAs and controls).

| miRBase ID | Assay ID (Applied Biosystems) |
| --- | --- |
| hsa-let-7a-5p | 000377 |
| hsa-let-7b-5p | 002619 |
| hsa-let-7c-3p | 000379 |
| hsa-let-7d-5p | 002283 |
| hsa-let-7e-5p | 002406 |
| hsa-let-7g-5p | 002282 |
| hsa-miR-100-5p | 000437 |
| hsa-miR-106a-5p | 002169 |
| hsa-miR-106b-5p | 000442 |
| hsa-miR-10a-5p | 000387 |
| hsa-miR-10b-3p | 002315 |
| hsa-miR-10b-5p | 002218 |
| hsa-miR-125a-5p | 002198 |
| hsa-miR-126-3p | 002228 |
| hsa-miR-127-3p | 000452 |
| hsa-mir-130a-3p | 000454 |
| hsa-miR-132-3p | 000457 |
| hsa-miR-138-5p | 002284 |
| hsa-miR-140-3p | 002234 |
| hsa-miR-143-3p | 002249 |
| hsa-miR-145-5p | 002278 |
| hsa-miR-146a-5p | 000468 |
| hsa-miR-146b-5p | 001097 |
| hsa-miR-151a-3p | 002254 |
| hsa-miR-151a-5p | 002642 |
| hsa-miR-152-3p | 000475 |
| hsa-miR-155-5p | 002623 |
| hsa-miR-15b-5p | 000390 |
| hsa-miR-16-5p | 000391 |
| hsa-miR-17-5p | 002308 |
| hsa-miR-181a-5p | 000480 |
| hsa-miR-186-5p | 002285 |
| hsa-miR-191-5p | 002299 |
| hsa-miR-193a-5p | 002281 |
| hsa-miR-193b-3p | 002367 |
| hsa-miR-196b-5p | 002215 |
| hsa-miR-19a-3p | 000395 |
| hsa-miR-204-5p | 000508 |
| hsa-miR-20a-5p | 000580 |
| hsa-miR-21-5p | 000397 |
| hsa-miR-214-3p | 002306 |
| hsa-miR-22-5p | 002301 |
| hsa-miR-221-3p | 000524 |
| hsa-miR-222-3p | 002276 |
| hsa-miR-224-5p | 002099 |
| hsa-miR-24-3p | 000402 |
| hsa-miR-26a-5p | 000405 |
| hsa-miR-26b-5p | 000407 |
| hsa-miR-27a-3p | 000408 |
| hsa-miR-27b-3p | 000409 |
| hsa-miR-29a-3p | 002112 |
| hsa-miR-31-3p | 002113 |
| hsa-miR-320a | 002277 |
| hsa-miR-339-3p | 002184 |
| hsa-miR-345-5p | 002186 |
| hsa-miR-34a-3p | 002316 |
| hsa-miR-34a-5p | 000426 |
| hsa-miR-365a-3p | 001020 |
| hsa-miR-370-3p | 002275 |
| hsa-miR-374a-5p | 000563 |
| hsa-miR-376c-3p | 002122 |
| hsa-miR-378a-3p | 002243 |
| hsa-miR-409-3p | 002332 |
| hsa-miR-425-5p | 001516 |
| hsa-miR-432-5p | 001026 |
| hsa-miR-454-3p | 002323 |
| hsa-miR-484 | 001821 |
| hsa-miR-494-3p | 002365 |
| hsa-miR-532-5p | 001518 |
| hsa-miR-574-3p | 002349 |
| hsa-miR-708-5p | 002341 |
| hsa-miR-92a-3p | 000431 |
| hsa-miR-93-3p | 002139 |
| hsa-miR-99b-5p | 000436 |
| hsa-miR-134-5p | 001186 |
| hsa-miR-137 | 001129 |
| hsa-miR-140-5p | 001187 |
| hsa-miR-374b-5p | 001319 |
| hsa-miR-93-5p | 001090 |
| U6 snRNA | 001973 |


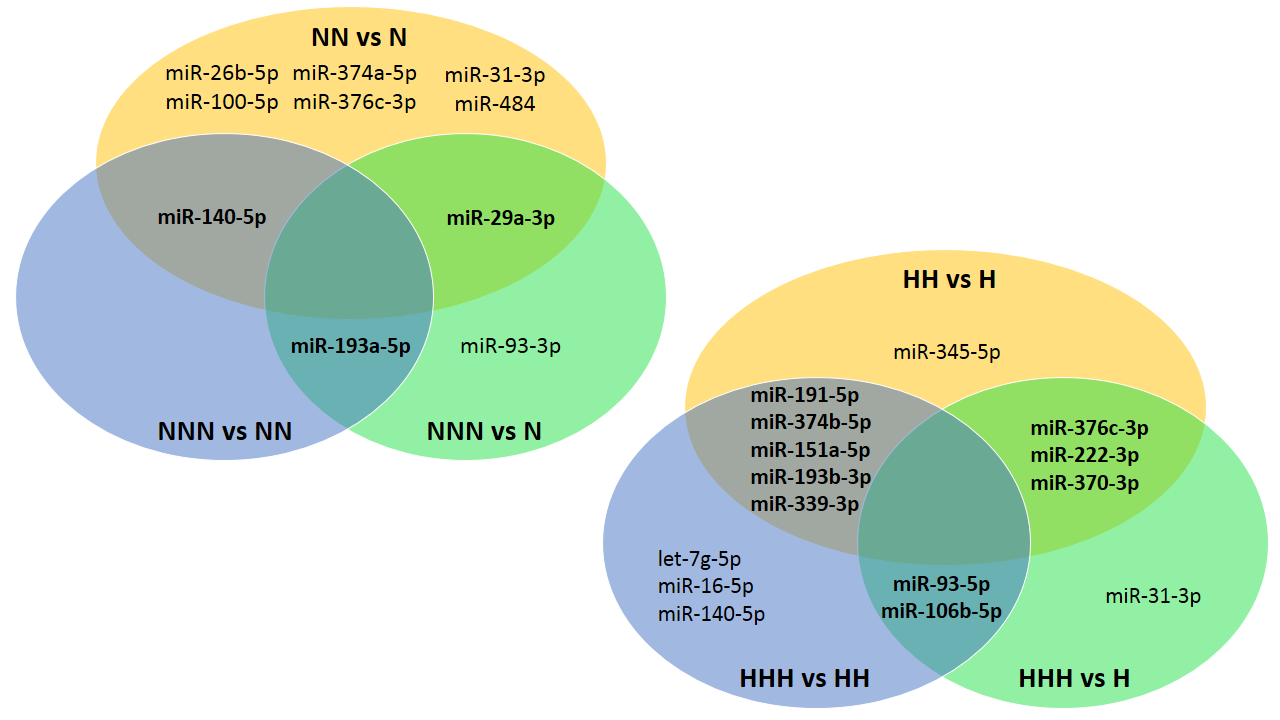


**Figure S1**. Differentially expressed miRNAs between particular stages of adipocyte development in NG (A) and HG (B). Shared miRNAs are bolded. Statistical significance was evaluated using ANOVA with Tukey post hoc test with p ≤ 0.05 considered as significant.


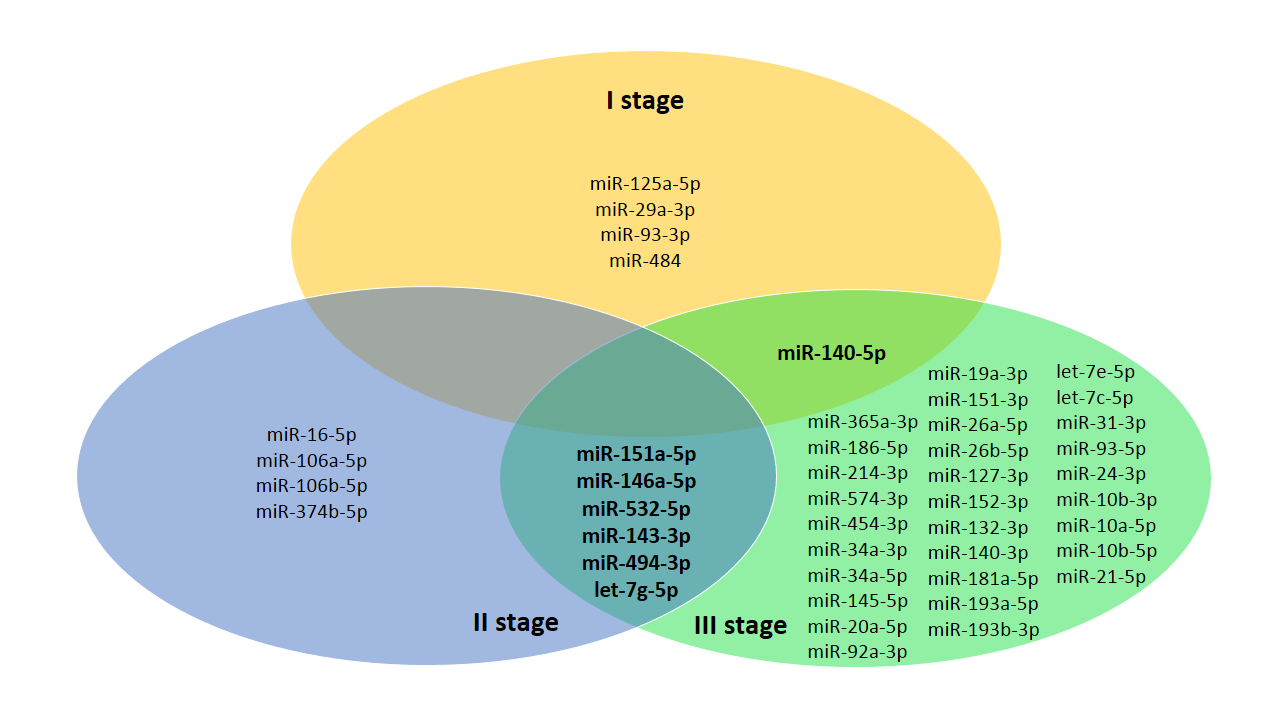


**Figure S2.** miRNAs significantly changed upon intermittent and chronic HG at the particular ADG stage. Shared miRNAs are bolded. Statistical significance was evaluated using two-tailed t-test with p ≤ 0.05 considered as significant.





**Figure S3.** The expression levels of miR-140-5p, miR-93-5p, miR-31-3p, miR-151a-5p, miR-106b-5p, miR-10a-5p, miR-193a-5p, miR-193-3p, miR-484, and miR-376c-3p obtained during the proliferation, differentiation, and maturation of Ads in normoglycemic and hyperglycemic conditions. Means are presented in a form of circles (NG) and triangles (HG). Data are expressed as mean ± SD. Statistical significance was evaluated using ANOVA with a Tukey post hoc test. (***), p ≤ 0.001 (**), p≤0.01 (*), p≤0.05 (#), 0.065≥p>0.05 (##), 0.085≥p>0.065.

Selected miRNAs expressed the trend for downregulation during ADG, except for miR-151a-5p, miR-193a-5p, and miR-193b-3p (Fig. S3). miR-140-5p, miR-31-3p, and miR-376c-3p were significantly regulated during ADG in both NG and HG. miR-140-5p showed pronounced differences in the direction of expression changes between NNN vesus NN and HHH versus HH, as suggested by global analyses. miR-106b-5p and miR-93-5p were gradually downregulated and almost reached the significance in all of the examined comparisons in HG-affected ADG.

Considering the effect of exposure to chronic/intermittent HG on miRNAs expression (Fig. S4), HG caused the decline of nearly all of the selected miRNAs in pAds (H versus N), yet only miR-140-5p, miR-484, and miR-193a-5p exhibited either a trend or statistically significant decrease. In turn, the majority of data collected for differentiated Ads exposed to HG at both the first and second stage (HH versus NN) implied no expression changes of the miRNAs, with upregulation suggested solely for miR-106b-5p and miR-140-5p. Mature Ads treated with HG during all of the stages (HHH versus NNN) exhibited at least the tendency of half of the selected miRNAs for expression reduction (Fig. S5). Consistently, miR-151a-5p, miR-140-5p, and miR-10a-5p were significantly decreased along with miR-31-3p, showing a statistically supported trend toward the decline.





**Figure S4.** The expression levels of miR-140-5p, miR-93-5p, miR-31-3p, miR-151a-5p, miR-106b-5p, miR-10a-5p, miR-193a-5p, miR-193-3p, miR-484, and miR-376c-3p obtained for pAds and differentiated Ads exposed to high glucose during the first and/or second stage of the cell culture. Means are presented in a form of circles (NG), squares (single HG hit), and triangles (chronic HG). Data are expressed as mean ± SD. Statistical significance was evaluated using the t-test. (***), p ≤ 0.001 (**), p≤0.01 (*), p≤0.05 (#), 0.065≥p>0.05 (##), 0.085≥p>0.065.

Next, we evaluated the impact of a single and double stimulus of HG on the expression pattern of miRNAs in differentiated Ads (Fig. S4). A single HG hit at any culture stage (first or second, HN versus NN, NH versus NN) did not exert statistically significant changes in the miRNAs’ expression. Changes of a particular miRNA in differentiated Ads exposed to HG in two stages in relation to a single HG stimulus (HHvsNH, HHvsHN) appeared to be similar, which was best depicted for miR-151a-5p and miR-106b-5p. miR-93-5p, mir-31-3p, and miR-376c-3p seemed to be entirely unresponsive to HG at the second stage of cell culture.

Single exposure to HG evoked a trend or significant decline of all of the miRNAs, except for miR-106b-5p, miR-484, and miR-376c-3p in mature Ads (Fig. S5). The implementation of HG at the second stage might have a significant impact on miRNAs’ expression, since miR-10a-5p, miR-93-5p, miR-31-3p, and miR-140-5p were significantly decreased, and miR-151a-5p, miR-193a-5p, and miR-193b-3p showed the tendency for expression reduction. miR-10a-5p was the most responsive one, since independently of the stage, single HG exposure evoked its significant downregulation, resembling changes occurring upon chronic HG.

Considering the effect of a double hit versus a single HG hit in mature Ads, we observed no changes in the expression of the miRNAs in HNH versus HNN (Fig. S5). The expression of the majority of miRNAs appeared to be either unchanged or moderately increased in HNH versus NNH and NHH versus NHN, as shown for miR-93-5p. We found a substantial decline for miR-193b-3p and no changes of miR-93-5p for NHH versus NNH. The only significant expression difference in HHN versus HNN was found for miR-193a-5p. Taking into account the number and magnitude of changes, it may be speculated that a double hit of HG does not exert a significant impact on the expression of selected miRNAs in comparison to a single stimulus.

Mature Ads exposed to chronic HG in relation to HG exposure at two stages appeared to show the tendency for the decrease of the majority of miRNAs, with the least changes observed for HHH versus NHH. Contrarily, miR-151a-5p was significantly but slightly upregulated in HHH versus NHH. Therefore, the restoration of NG at the solely single stage might be not sufficient to reverse the direction of expression changes evoked upon chronic HG (Fig. S5).





**Fig. S5** The relative expression levels of miR-140-5p, miR-93-5p, miR-31-3p, miR-151a-5p, miR-106b-5p, miR-10a-5p, miR-193a-5p, miR-193-3p, miR-484, and miR-376c-3p obtained for mature Ads treated with chronic and intermittent high glucose during three stages of cell culture. Means are presented in a form of circles (NG), squares (single HG hit), triangles (chronic HG), and grey lines (double HG hit). Data are expressed as mean ± SD. Statistical significance was evaluated using t-test. (***), p ≤ 0.001 (**), p≤0.01 (*), p≤0.05 (#), 0.065≥p>0.05 (##), 0.085≥ p >0.065.

**Table S2.** KEGG pathways found to be enriched for 15 core miRNAs, generated by DIANA-miRPath v3 and merged by genes union. ECM – extracellular matrix, TGF – tumor growth factor, TCA – tricarboxylic acid, FOXO – forkhead box protein O. TNF – tumor necrosis factor, mTOR – mammalian target of rapamycin, HIF-1 – hypoxia-inducible factor 1, MAPK – mitogen activated kinase, HTLV -human T-lymphotropic virus.

| **#** | **KEGG pathway** | **p value** | **#genes** | **#miRNAs** |
| --- | --- | --- | --- | --- |
| **1.** | Początek formularza  ECM–receptor interaction (hsa04512)  Dół formularza | 5.50659709632e-15 | Początek formularza  53  Dół formularza | 15  Początek formularza  Dół formularza  Początek formularza  Dół formularza |
| **2.** | Początek formularza  Cell cycle (hsa04110)  Dół formularza | 1.07523327402e-12 | Początek formularza  95  Dół formularza | 14  Początek formularza  Dół formularza  Początek formularza  Dół formularza |
| **3.** | Początek formularza  Protein processing in endoplasmic reticulum (hsa04141)  Dół formularza | 9.85755276872e-11 | Początek formularza  123  Dół formularza | 15  Początek formularza  Dół formularza  Początek formularza  Dół formularza |
| **4.** | Początek formularza  Adherens junction (hsa04520)  Dół formularza | 9.85755276872e-11 | Początek formularza  57  Dół formularza | 15  Początek formularza  Dół formularza  Początek formularza  Dół formularza |
| **5.** | Początek formularza  Viral carcinogenesis (hsa05203)  Dół formularza | 7.8444268487e-09 | Początek formularza  133  Dół formularza | 14  Początek formularza  Dół formularza  Początek formularza  Dół formularza |
| **6.** | Początek formularza  Proteoglycans in cancer (hsa05205)  Dół formularza | 9.60223229716e-08 | Początek formularza  129  Dół formularza | 15  Początek formularza  Dół formularza  Początek formularza  Dół formularza |
| **7.** | Początek formularza  Glycosaminoglycan biosynthesis: chondroitin sulfate / dermatan sulfate (hsa00532)  Dół formularza | 9.85099532287e-08 | Początek formularza  15  Dół formularza | 8  Początek formularza  Dół formularza  Początek formularza  Dół formularza |
| **8.** | Początek formularza  Ubiquitin-mediated proteolysis (hsa04120)  Dół formularza | 9.85099532287e-08 | Początek formularza  101  Dół formularza | 15  Początek formularza  Dół formularza  Początek formularza  Dół formularza |
| **9.** | Początek formularza  Fatty acid metabolism (hsa01212)  Dół formularza | 1.9313695501e-07 | Początek formularza  30  Dół formularza | 13  Początek formularza  Dół formularza  Początek formularza  Dół formularza |
| **10.** | Początek formularza  TGF-beta signaling pathway (hsa04350)  Dół formularza | 1.9313695501e-07 | Początek formularza  57  Dół formularza | 14  Początek formularza  Dół formularza  Początek formularza  Dół formularza |
| **11.** | Początek formularza  p53 signaling pathway (hsa04115)  Dół formularza | 4.44606810456e-06 | Początek formularza  54  Dół formularza | 14  Początek formularza  Dół formularza  Początek formularza  Dół formularza |
| **12.** | Początek formularza  Fatty acid elongation (hsa00062)  Dół formularza | 5.25204024506e-06 | Początek formularza  16  Dół formularza | 9  Początek formularza  Dół formularza  Początek formularza  Dół formularza |
| **13.** | Początek formularza  Hippo signaling pathway (hsa04390)  Dół formularza | 8.97890509783e-06 | Początek formularza  90  Dół formularza | 14  Początek formularza  Dół formularza  Początek formularza  Dół formularza |
| **14.** | Początek formularza  Endocytosis (hsa04144)  Dół formularza | 1.05812050084e-05 | Początek formularza  133  Dół formularza | 15  Początek formularza  Dół formularza  Początek formularza  Dół formularza |
| **15.** | Początek formularza  Hepatitis B (hsa05161)  Dół formularza | 1.24317213852e-05 | Początek formularza  92  Dół formularza | 15  Początek formularza  Dół formularza  Początek formularza  Dół formularza |
| **16.** | Początek formularza  Colorectal cancer (hsa05210)  Dół formularza | 1.78056223784e-05 | Początek formularza  47  Dół formularza | 14  Początek formularza  Dół formularza  Początek formularza  Dół formularza |
| **17.** | Początek formularza  RNA transport (hsa03013)  Dół formularza | 1.82760277006e-05 | Początek formularza  113  Dół formularza | 15  Początek formularza  Dół formularza  Początek formularza  Dół formularza |
| **18.** | Początek formularza  Oocyte meiosis (hsa04114)  Dół formularza | 2.53966876082e-05 | Początek formularza  72  Dół formularza | 15  Początek formularza  Dół formularza  Początek formularza  Dół formularza |
| **19.** | Początek formularza  Renal cell carcinoma (hsa05211)  Dół formularza | 3.46425253178e-05 | Początek formularza  48  Dół formularza | 13  Początek formularza  Dół formularza  Początek formularza  Dół formularza |
| **20.** | Początek formularza  Steroid biosynthesis (hsa00100)  Dół formularza | 8.88746020464e-05 | Początek formularza  16  Dół formularza | 12  Początek formularza  Dół formularza  Początek formularza  Dół formularza |
| **21.** | Początek formularza  Focal adhesion (hsa04510)  Dół formularza | 9.62611742949e-05 | Początek formularza  133  Dół formularza | 15  Początek formularza  Dół formularza  Początek formularza  Dół formularza |
| **22.** | Początek formularza  Citrate cycle (TCA cycle) (hsa00020)  Dół formularza | 0.000130256326189 | Początek formularza  23  Dół formularza | 11  Początek formularza  Dół formularza  Początek formularza  Dół formularza |
| **23.** | Początek formularza  Prion diseases (hsa05020)  Dół formularza | 0.000130256326189 | Początek formularza  17  Dół formularza | 13  Początek formularza  Dół formularza  Początek formularza  Dół formularza |
| **24.** | Początek formularza  Pancreatic cancer (hsa05212)  Dół formularza | 0.000141238344866 | Początek formularza  49  Dół formularza | 15  Początek formularza  Dół formularza  Początek formularza  Dół formularza |
| **25.** | Początek formularza  Lysine degradation (hsa00310)  Dół formularza | 0.000193050547461 | Początek formularza  32  Dół formularza | 12  Początek formularza  Dół formularza  Początek formularza  Dół formularza |
| **26.** | Początek formularza  Prostate cancer (hsa05215)  Dół formularza | 0.000198425177168 | Początek formularza  62  Dół formularza | 15  Początek formularza  Dół formularza  Początek formularza  Dół formularza |
| **27.** | Początek formularza  Spliceosome (hsa03040)  Dół formularza | 0.000262922729802 | Początek formularza  87  Dół formularza | 15  Początek formularza  Dół formularza  Początek formularza  Dół formularza |
| **28.** | Początek formularza  Glycosaminoglycan biosynthesis: keratan sulfate (hsa00533)  Dół formularza | 0.000299329652599 | Początek formularza  10  Dół formularza | 8  Początek formularza  Dół formularza  Początek formularza  Dół formularza |
| **29.** | Początek formularza  Thyroid hormone signaling pathway (hsa04919)  Dół formularza | 0.000486118447111 | Początek formularza  76  Dół formularza | 15  Początek formularza  Dół formularza  Początek formularza  Dół formularza |
| **30.** | Początek formularza  Small cell lung cancer (hsa05222)  Dół formularza | 0.00108450129143 | Początek formularza  59  Dół formularza | 15  Początek formularza  Dół formularza  Początek formularza  Dół formularza |
| **31.** | Początek formularza  Inositol phosphate metabolism (hsa00562)  Dół formularza | 0.00112562955159 | Początek formularza  43  Dół formularza | 15  Początek formularza  Dół formularza  Początek formularza  Dół formularza |
| **32.** | Początek formularza  mRNA surveillance pathway (hsa03015)  Dół formularza | 0.00119481539523 | Początek formularza  62  Dół formularza | 15  Początek formularza  Dół formularza  Początek formularza  Dół formularza |
| **33.** | Początek formularza  FOXO signaling pathway (hsa04068)  Dół formularza | 0.00160742619827 | Początek formularza  86  Dół formularza | 15  Początek formularza  Dół formularza  Początek formularza  Dół formularza |
| **34.** | Początek formularza  Pathways in cancer (hsa05200)  Dół formularza | 0.00183743468407 | Początek formularza  230  Dół formularza | 15  Początek formularza  Dół formularza  Początek formularza  Dół formularza |
| **35.** | Początek formularza  N-glycan biosynthesis (hsa00510)  Dół formularza | 0.00295950973562 | Początek formularza  31  Dół formularza | 15  Początek formularza  Dół formularza  Początek formularza  Dół formularza |
| **36.** | Początek formularza  Chronic myeloid leukemia (hsa05220)  Dół formularza | 0.00317603382806 | Początek formularza  49  Dół formularza | 15  Początek formularza  Dół formularza  Początek formularza  Dół formularza |
| **37.** | Początek formularza  Transcriptional misregulation in cancer (hsa05202)  Dół formularza | 0.00336342428957 | Początek formularza  104  Dół formularza | 15  Początek formularza  Dół formularza  Początek formularza  Dół formularza |
| **38.** | Początek formularza  Endometrial cancer (hsa05213)  Dół formularza | 0.00368434280041 | Początek formularza  36  Dół formularza | 15  Początek formularza  Dół formularza  Początek formularza  Dół formularza |
| **39.** | Początek formularza  DNA replication (hsa03030)  Dół formularza | 0.00402078208387 | Początek formularza  26  Dół formularza | 8  Początek formularza  Dół formularza  Początek formularza  Dół formularza |
| **40.** | Początek formularza  Sphingolipid signaling pathway (hsa04071)  Dół formularza | 0.00416875942682 | Początek formularza  73  Dół formularza | 15  Początek formularza  Dół formularza  Początek formularza  Dół formularza |
| **41.** | Początek formularza  Bladder cancer (hsa05219)  Dół formularza | 0.00455171648349 | Początek formularza  29  Dół formularza | 14  Początek formularza  Dół formularza  Początek formularza  Dół formularza |
| **42.** | Początek formularza  TNF signaling pathway (hsa04668)  Dół formularza | 0.00513525989477 | Początek formularza  69  Dół formularza | 15  Początek formularza  Dół formularza  Początek formularza  Dół formularza |
| **43.** | Początek formularza  mTOR signaling pathway (hsa04150)  Dół formularza | 0.00556952027852 | Początek formularza  42  Dół formularza | 15  Początek formularza  Dół formularza  Początek formularza  Dół formularza |
| **44.** | Początek formularza  Bacterial invasion of epithelial cells (hsa05100)  Dół formularza | 0.00590183500045 | Początek formularza  49  Dół formularza | 14 |
| **45.** | Początek formularza  Glioma (hsa05214)  Dół formularza | 0.00843150566238 | Początek formularza  41  Dół formularza | 14 |
| **46.** | Początek formularza  Non-small cell lung cancer (hsa05223)  Dół formularza | 0.00918926744091 | Początek formularza  37  Dół formularza | 15 |
| **47.** | Początek formularza  Fatty acid biosynthesis (hsa00061)  Dół formularza | 0.00950499739796 | Początek formularza  6  Dół formularza | 11 |
| **48.** | Początek formularza  Regulation of actin cytoskeleton (hsa04810)  Dół formularza | 0.00950499739796 | Początek formularza  123  Dół formularza | 15 |
| **49.** | Początek formularza  HIF-1 signaling pathway (hsa04066)  Dół formularza | 0.0105053120353 | Początek formularza  67  Dół formularza | 15 |
| **50.** | Początek formularza  Epithelial cell signaling in Helicobacter pylori infection (hsa05120)  Dół formularza | 0.0115704510188 | Początek formularza  45  Dół formularza | 14 |
| **51.** | Początek formularza  Central carbon metabolism in cancer (hsa05230)  Dół formularza | 0.0115704510188 | Początek formularza  41  Dół formularza | 14 |
| **52.** | Początek formularza  Amoebiasis (hsa05146)  Dół formularza | 0.0137235027183 | Początek formularza  63  Dół formularza | 15 |
| **53.** | Początek formularza  MAPK signaling pathway (hsa04010)  Dół formularza | 0.0143155500161 | Początek formularza  145  Dół formularza | 15 |
| **54.** | Początek formularza  Estrogen signaling pathway (hsa04915)  Dół formularza | 0.0143155500161 | Początek formularza  60  Dół formularza | 15 |
| **55.** | Początek formularza  Circadian rhythm (hsa04710)  Dół formularza | 0.0155242782123 | Początek formularza  22  Dół formularza | 13 |
| **56.** | Początek formularza  Huntington's disease (hsa05016)  Dół formularza | 0.0171382931179 | Początek formularza  106  Dół formularza | 15 |
| **57.** | Początek formularza  Other types of O-glycan biosynthesis (hsa00514)  Dół formularza | 0.0245187689224 | Początek formularza  19  Dół formularza | 11 |
| **58.** | Początek formularza  Terpenoid backbone biosynthesis (hsa00900)  Dół formularza | 0.0253063675488 | Początek formularza  15  Dół formularza | 11 |
| **59.** | Początek formularza  Shigellosis (hsa05131)  Dół formularza | 0.0254984769267 | Początek formularza  41  Dół formularza | 13 |
| **60.** | Początek formularza  Acute myeloid leukemia (hsa05221)  Dół formularza | 0.0316949991829 | Początek formularza  38  Dół formularza | 14 |
| **61.** | Początek formularza  Phosphatidylinositol signaling system (hsa04070)  Dół formularza | 0.0316949991829 | Początek formularza  52  Dół formularza | 15 |
| **62.** | Początek formularza  Progesterone-mediated oocyte maturation (hsa04914)  Dół formularza | 0.0333868106205 | Początek formularza  55  Dół formularza | 14 |
| **63.** | Początek formularza  Thyroid cancer (hsa05216)  Dół formularza | 0.0354872190772 | Początek formularza  20  Dół formularza | 13 |
| **64.** | Początek formularza  HTLV-I infection (hsa05166)  Dół formularza | 0.0430528873739 | Początek formularza  145  Dół formularza | 15 |
| **65.** | Początek formularza  Axon guidance (hsa04360)  Dół formularza | 0.0468352682091 | Początek formularza  68  Dół formularza | 14 |
| **66.** | Początek formularza  Epstein–Barr virus infection (hsa05169)  Dół formularza | 0.0488276315313 | Początek formularza  120  Dół formularza | 14 |

**Table S3.** KEGG pathways found to be enriched for 15 core miRNAs, generated by DIANA-miRPath v3 and merged by pathways union.

| **#** | **KEGG pathway** | **p value** | **#genes** | **#miRNAs** |
| --- | --- | --- | --- | --- |
| 1. | Początek formularza  Prion diseases (hsa05020)  Dół formularza | <1e-325 | Początek formularza  13  Dół formularza | 4  Początek formularza  Dół formularza  Początek formularza  Dół formularza |
| 2. | Początek formularza  Fatty acid biosynthesis (hsa00061)  Dół formularza | <1e-325 | Początek formularza  4  Dół formularza | 5  Początek formularza  Dół formularza  Początek formularza  Dół formularza |
| 3. | Początek formularza  Fatty acid metabolism (hsa01212)  Dół formularza | <1e-325 | Początek formularza  26  Dół formularza | 6  Początek formularza  Dół formularza  Początek formularza  Dół formularza |
| 4. | Początek formularza  ECM–receptor interaction (hsa04512)  Dół formularza | <1e-325 | Początek formularza  35  Dół formularza | 6  Początek formularza  Dół formularza  Początek formularza  Dół formularza |
| 5. | Początek formularza  Viral carcinogenesis (hsa05203)  Dół formularza | <1e-325 | Początek formularza  127  Dół formularza | 6  Początek formularza  Dół formularza  Początek formularza  Dół formularza |
| 6. | Początek formularza  Cell cycle (hsa04110)  Dół formularza | <1e-325 | Początek formularza  78  Dół formularza | 7  Początek formularza  Dół formularza  Początek formularza  Dół formularza |
| 7. | Początek formularza  Proteoglycans in cancer (hsa05205)  Dół formularza | <1e-325 | Początek formularza  121  Dół formularza | 10  Początek formularza  Dół formularza  Początek formularza  Dół formularza |
| 8. | Początek formularza  p53 signaling pathway (hsa04115)  Dół formularza | 7.926992e-14 | Początek formularza  53  Dół formularza | 9  Początek formularza  Dół formularza  Początek formularza  Dół formularza |
| 9. | Początek formularza  Hepatitis B (hsa05161)  Dół formularza | 1.859624e-13 | Początek formularza  90  Dół formularza | 7  Początek formularza  Dół formularza  Początek formularza  Dół formularza |
| 10. | Początek formularza  Hippo signaling pathway (hsa04390)  Dół formularza | 2.88769e-13 | Początek formularza  88  Dół formularza | 8  Początek formularza  Dół formularza  Początek formularza  Dół formularza |
| 11. | Początek formularza  Lysine degradation (hsa00310)  Dół formularza | 5.722089e-13 | Początek formularza  29  Dół formularza | 8  Początek formularza  Dół formularza  Początek formularza  Dół formularza |
| 12. | Początek formularza  Adherens junction (hsa04520)  Dół formularza | 1.769684e-11 | Początek formularza  55  Dół formularza | 7  Początek formularza  Dół formularza  Początek formularza  Dół formularza |
| 13. | Początek formularza  Glioma (hsa05214)  Dół formularza | 9.768841e-11 | Początek formularza  37  Dół formularza | 7  Początek formularza  Dół formularza  Początek formularza  Dół formularza |
| 14. | Początek formularza  Pathways in cancer (hsa05200)  Dół formularza | 3.589052e-10 | Początek formularza  198  Dół formularza | 7  Początek formularza  Dół formularza  Początek formularza  Dół formularza |
| 15. | Początek formularza  TGF-beta signaling pathway (hsa04350)  Dół formularza | 4.622362e-10 | Początek formularza  49  Dół formularza | 5  Początek formularza  Dół formularza  Początek formularza  Dół formularza |
| 16. | Początek formularza  Chronic myeloid leukemia (hsa05220)  Dół formularza | 2.565229e-09 | Początek formularza  46  Dół formularza | 8  Początek formularza  Dół formularza  Początek formularza  Dół formularza |
| 17. | Początek formularza  Protein processing in endoplasmic reticulum (hsa04141)  Dół formularza | 1.422962e-08 | Początek formularza  107  Dół formularza | 5  Początek formularza  Dół formularza  Początek formularza  Dół formularza |
| 18. | Początek formularza  Oocyte meiosis (hsa04114)  Dół formularza | 1.96818e-07 | Początek formularza  65  Dół formularza | 7  Początek formularza  Dół formularza  Początek formularza  Dół formularza |
| 19. | Początek formularza  FOXO signaling pathway (hsa04068)  Dół formularza | 3.181776e-07 | Początek formularza  63  Dół formularza | 6  Początek formularza  Dół formularza  Początek formularza  Dół formularza |
| 20. | Początek formularza  Colorectal cancer (hsa05210)  Dół formularza | 1.272083e-06 | Początek formularza  43  Dół formularza | 7  Początek formularza  Dół formularza  Początek formularza  Dół formularza |
| 21. | Początek formularza  Prostate cancer (hsa05215)  Dół formularza | 4.716824e-06 | Początek formularza  57  Dół formularza | 6  Początek formularza  Dół formularza  Początek formularza  Dół formularza |
| 22. | Początek formularza  Bladder cancer (hsa05219)  Dół formularza | 6.927875e-06 | Początek formularza  26  Dół formularza | 5  Początek formularza  Dół formularza  Początek formularza  Dół formularza |
| 23. | Początek formularza  Renal cell carcinoma (hsa05211)  Dół formularza | 4.525012e-05 | Początek formularza  31  Dół formularza | 5  Początek formularza  Dół formularza  Początek formularza  Dół formularza |
| 24. | Początek formularza  Glycosaminoglycan biosynthesis: chondroitin sulfate / dermatan sulfate (hsa00532)  Dół formularza | 5.341373e-05 | Początek formularza  13  Dół formularza | 3  Początek formularza  Dół formularza  Początek formularza  Dół formularza |
| 25. | Początek formularza  Steroid biosynthesis (hsa00100)  Dół formularza | 5.758431e-05 | Początek formularza  9  Dół formularza | 4  Początek formularza  Dół formularza  Początek formularza  Dół formularza |
| 26. | Początek formularza  RNA transport (hsa03013)  Dół formularza | 8.531346e-05 | Początek formularza  83  Dół formularza | 4  Początek formularza  Dół formularza  Początek formularza  Dół formularza |
| 27. | Początek formularza  Pancreatic cancer (hsa05212)  Dół formularza | 0.0001034406 | Początek formularza  46  Dół formularza | 6  Początek formularza  Dół formularza  Początek formularza  Dół formularza |
| 28. | Początek formularza  Melanoma (hsa05218)  Dół formularza | 0.0001449095 | Początek formularza  27  Dół formularza | 4  Początek formularza  Dół formularza  Początek formularza  Dół formularza |
| 29. | Początek formularza  Signaling pathways regulating pluripotency of stem cells (hsa04550)  Dół formularza | 0.0002915358 | Początek formularza  59  Dół formularza | 3  Początek formularza  Dół formularza  Początek formularza  Dół formularza |
| 30. | Początek formularza  Thyroid hormone signaling pathway (hsa04919)  Dół formularza | 0.0003177942 | Początek formularza  42  Dół formularza | 2  Początek formularza  Dół formularza  Początek formularza  Dół formularza |
| 31. | Początek formularza  Focal adhesion (hsa04510)  Dół formularza | 0.0007792229 | Początek formularza  84  Dół formularza | 4  Początek formularza  Dół formularza  Początek formularza  Dół formularza |
| 32. | Początek formularza  Arrhythmogenic right ventricular cardiomyopathy (ARVC) (hsa05412)  Dół formularza | 0.001435434 | Początek formularza  9  Dół formularza | 3  Początek formularza  Dół formularza  Początek formularza  Dół formularza |
| 33. | Początek formularza  Small cell lung cancer (hsa05222)  Dół formularza | 0.001733236 | Początek formularza  53  Dół formularza | 4  Początek formularza  Dół formularza  Początek formularza  Dół formularza |
| 34. | Początek formularza  Bacterial invasion of epithelial cells (hsa05100)  Dół formularza | 0.003886583 | Początek formularza  42  Dół formularza | 5  Początek formularza  Dół formularza  Początek formularza  Dół formularza |
| 35. | Początek formularza  Thyroid cancer (hsa05216)  Dół formularza | 0.004521831 | Początek formularza  17  Dół formularza | 4  Początek formularza  Dół formularza  Początek formularza  Dół formularza |
| 36. | Początek formularza  Endocytosis (hsa04144)  Dół formularza | 0.004600827 | Początek formularza  82  Dół formularza | 4  Początek formularza  Dół formularza  Początek formularza  Dół formularza |
| 37. | Początek formularza  Non-small cell lung cancer (hsa05223)  Dół formularza | 0.004793285 | Początek formularza  23  Dół formularza | 3  Początek formularza  Dół formularza  Początek formularza  Dół formularza |
| 38. | Początek formularza  Ubiquitin-mediated proteolysis (hsa04120)  Dół formularza | 0.004834829 | Początek formularza  73  Dół formularza | 4  Początek formularza  Dół formularza  Początek formularza  Dół formularza |
| 39. | Początek formularza  MAPK signaling pathway (hsa04010)  Dół formularza | 0.006668105 | Początek formularza  87  Dół formularza | 4  Początek formularza  Dół formularza  Początek formularza  Dół formularza |
| 40. | Początek formularza  PI3K–Akt signaling pathway (hsa04151)  Dół formularza | 0.008960904 | Początek formularza  111  Dół formularza | 3  Początek formularza  Dół formularza  Początek formularza  Dół formularza |
| 41. | Początek formularza  Endometrial cancer (hsa05213)  Dół formularza | 0.01310239 | Początek formularza  19  Dół formularza | 3  Początek formularza  Dół formularza  Początek formularza  Dół formularza |
| 42. | Początek formularza  mTOR signaling pathway (hsa04150)  Dół formularza | 0.01669236 | Początek formularza  27  Dół formularza | 3  Początek formularza  Dół formularza  Początek formularza  Dół formularza |
| 43. | Początek formularza  Biosynthesis of unsaturated fatty acids (hsa01040)  Dół formularza | 0.02643918 | Początek formularza  7  Dół formularza | 3 |

**Table S4.** Overrepresentation analysis performed by miEAA for 15 core miRNAs with statistically significant records sorted by p value. JAK/STAT - Janus Kinase / Signal Transducer and Activator of Transcription, VEGF – vascular endothelial growth factor, HSP – heat shock protein, bZIP (basic region, leucine zipper), EPO – erythropoietin, GnRH - gonadotropin-releasing hormone, FGF – fibroblast growth factor, SNARE - "SNAP" (Soluble NSF Attachment Protein) Receptor, NOD - nucleotide-binding oligomerization domain, EGF/EGFR - (epidermal growth factor receptor), GPCR - G Protein-Coupled Receptor, TCR - T-cell receptor, GABA - gamma-aminobutyric acid, Errb - Erb-b2 receptor tyrosine kinases, NR3C – nuclear receptor subfamily group C, ELK1 - ETS domain-containing protein, SRF - Serum Response Factor, PDGF - platelet-derived growth factor, RANKL - Receptor Activator for Nuclear Factor κ B Ligand, RANK - Receptor Activator for Nuclear Factor κ B, DDR – DNA damage response.

| Rank | Subcategory | p value | #miRNAs |
| --- | --- | --- | --- |
| 1 | hsa04350 TGF beta signaling pathway | 0.00050508 | 14 |
| 2 | P00052 TGF beta signaling pathway | 0.00129949 | 13 |
| 3 | WP400 p38 MAPK Signaling Pathway | 0.00129949 | 11 |
| 4 | WP241 One Carbon Metabolism | 0.00145494 | 9 |
| 5 | P00009 Axon guidance mediated by netrin | 0.00429261 | 8 |
| 6 | P02738 De novo purine biosynthesis | 0.00429261 | 8 |
| 7 | P02743 Formyltetrahydroformate biosynthesis | 0.00429261 | 5 |
| 8 | P02753 Methionine biosynthesis | 0.00429261 | 3 |
| 9 | P02773 S-adenosylmethionine biosynthesis | 0.00429261 | 4 |
| 10 | P04393 Ras Pathway | 0.00429261 | 11 |
| 11 | WP205 Interleukin (IL)-7 signaling pathway | 0.00429261 | 10 |
| 12 | WP384 Apoptosis Modulation by HSP70 | 0.00429261 | 7 |
| 13 | WP395 IL-4 signaling pathway | 0.00429261 | 11 |
| 14 | WP524 G13 signaling Pathway | 0.00429261 | 9 |
| 15 | hsa00270 Cysteine and methionine metabolism | 0.00429261 | 9 |
| 16 | hsa00630 Glyoxylate and dicarboxylate metabolism | 0.00429261 | 6 |
| 17 | hsa00910 Nitrogen metabolism | 0.00429261 | 7 |
| 18 | hsa00970 Aminoacyl tRNA biosynthesis | 0.00429261 | 8 |
| 19 | hsa03022 Basal transcription factors | 0.00429261 | 7 |
| 20 | hsa03030 DNA replication | 0.00429261 | 8 |
| 21 | hsa03410 Base excision repair | 0.00429261 | 8 |
| 22 | hsa04060 Cytokine–cytokine receptor interaction | 0.00429261 | 12 |
| 23 | hsa04630 JAK/STAT signaling pathway | 0.00429261 | 12 |
| 24 | hsa04722 Neurotrophin signaling pathway | 0.00429261 | 14 |
| 25 | hsa04960 Aldosterone-regulated sodium reabsorption | 0.00429261 | 9 |
| 26 | P00011 Blood coagulation | 0.00507701 | 7 |
| 27 | P00032 Insulin-like growth factor (IGF) pathway mitogen-activated protein kinase kinase MAP kinase cascade | 0.00507701 | 9 |
| 28 | WP143 Fatty acid beta oxidation | 0.00507701 | 7 |
| 29 | WP366 TGF beta signaling pathway1 | 0.00507701 | 13 |
| 30 | WP382 MAPK signaling pathway | 0.00507701 | 13 |
| 31 | hsa00350 Tyrosine metabolism | 0.00507701 | 6 |
| 32 | hsa03430 Mismatch repair | 0.00507701 | 7 |
| 33 | WP53 Id Signaling Pathway | 0.0051891 | 7 |
| 34 | hsa00071 Fatty acid metabolism | 0.0051891 | 7 |
| 35 | P02775 Salvage pyrimidine ribonucleotides | 0.00588723 | 4 |
| 36 | WP195 IL-1 signaling pathway | 0.00624053 | 10 |
| 37 | hsa04512 ECM receptor interaction | 0.00671137 | 9 |
| 38 | hsa04660 T-cell receptor signaling pathway | 0.00671137 | 11 |
| 39 | P02730 Asparagine and aspartate biosynthesis | 0.00689903 | 4 |
| 40 | hsa03040 Spliceosome | 0.00689903 | 11 |
| 41 | P02787 Vitamin B6 metabolism | 0.00722257 | 4 |
| 42 | WP466 DNA replication | 0.00722257 | 8 |
| 43 | WP500 Glycogen metabolism | 0.00722257 | 7 |
| 44 | WP615 Senescence and autophagy | 0.00722257 | 13 |
| 45 | hsa00051 Fructose and mannose metabolism | 0.00722257 | 7 |
| 46 | hsa00062 Fatty acid elongation in mitochondria | 0.00722257 | 5 |
| 47 | hsa00240 Pyrimidine metabolism | 0.00722257 | 9 |
| 48 | hsa00670 One carbon pool by folate | 0.00722257 | 6 |
| 49 | hsa00983 Drug metabolism other enzymes | 0.00722257 | 6 |
| 50 | hsa04260 Cardiac muscle contraction | 0.00722257 | 8 |
| 51 | hsa04310 Wnt signaling pathway | 0.00722257 | 12 |
| 52 | hsa04360 Axon guidance | 0.00722257 | 11 |
| 53 | hsa05120 Epithelial cell signaling in Helicobacter pylori infection | 0.00722257 | 10 |
| 54 | hsa05216 Thyroid cancer | 0.00722257 | 10 |
| 55 | WP698 Glucuronidation | 0.00765271 | 5 |
| 56 | hsa05416 Viral myocarditis | 0.00765271 | 10 |
| 57 | P00013 Cell cycle | 0.00786613 | 8 |
| 58 | P02723 Adenine and hypoxanthine salvage pathway | 0.00786613 | 4 |
| 59 | hsa04010 MAPK signaling pathway | 0.00786613 | 13 |
| 60 | P00007 Axon guidance mediated by semaphorins | 0.00829807 | 6 |
| 61 | P00050 Plasminogen-activating cascade | 0.00829807 | 5 |
| 62 | WP306 Focal adhesion | 0.00829807 | 13 |
| 63 | WP404 Nucleotide metabolism | 0.00829807 | 7 |
| 64 | hsa00130 Ubiquinone and other terpenoid quinone biosynthesis | 0.00829807 | 3 |
| 65 | hsa03420 Nucleotide excision repair | 0.00829807 | 7 |
| 66 | P00053 T-cell activation | 0.00835306 | 10 |
| 67 | WP107 Translation factors | 0.00835306 | 9 |
| 68 | WP477 Cytoplasmic ribosomal proteins | 0.00835306 | 11 |
| 69 | hsa00520 Amino sugar and nucleotide sugar metabolism | 0.00835306 | 7 |
| 70 | hsa04114 Oocyte meiosis | 0.00835306 | 11 |
| 71 | hsa05217 Basal cell carcinoma | 0.00909667 | 8 |
| 72 | P00023 General transcription regulation | 0.00949293 | 6 |
| 73 | P00060 Ubiquitin proteasome pathway | 0.00959124 | 8 |
| 74 | hsa00750 Vitamin B6 metabolism | 0.00985102 | 4 |
| 75 | P02770 Pyridoxal phosphate salvage pathway | 0.00999362 | 3 |
| 76 | hsa00500 Starch and sucrose metabolism | 0.00999362 | 6 |
| 77 | hsa04144 Endocytosis | 0.00999362 | 12 |
| 78 | WP364 IL 6 signaling pathway | 0.0101279 | 9 |
| 79 | hsa00310 Lysine degradation | 0.0101279 | 8 |
| 80 | hsa00830 Retinol metabolism | 0.0101279 | 5 |
| 81 | hsa04514 Cell adhesion molecules (CAMs) | 0.0101279 | 8 |
| 82 | hsa04720 Long-term potentiation | 0.0101279 | 9 |
| 83 | WP1544 MicroRNAs in cardiomyocyte hypertrophy | 0.0101811 | 10 |
| 84 | hsa00052 Galactose metabolism | 0.0101811 | 6 |
| 85 | hsa03440 Homologous recombination | 0.0101811 | 6 |
| 86 | WP368 Mitochondrial LC fatty acid beta oxidation | 0.0102228 | 5 |
| 87 | WP51 Regulation of actin cytoskeleton | 0.0102228 | 11 |
| 88 | hsa00040 Pentose and glucuronate interconversions | 0.0102228 | 4 |
| 89 | hsa00260 Glycine serine and threonine metabolism | 0.0102228 | 5 |
| 90 | hsa00534 Heparan sulfate biosynthesis | 0.0102228 | 5 |
| 91 | P00008 Axon guidance mediated by Slit-Robo | 0.0104838 | 6 |
| 92 | hsa04370 VEGF signaling pathway | 0.011034 | 10 |
| 93 | hsa04664 FC epsilon RI signaling pathway | 0.011034 | 9 |
| 94 | hsa05221 Acute myeloid leukemia | 0.011034 | 10 |
| 95 | P02721 ATP synthesis | 0.0111023 | 5 |
| 96 | P00015 Circadian clock system | 0.0111182 | 4 |
| 97 | hsa00400 Phenylalanine tyrosine and tryptophan biosynthesis | 0.0111182 | 3 |
| 98 | hsa05210 Colorectal cancer | 0.0111182 | 12 |
| 99 | hsa05218 Melanoma | 0.0111182 | 12 |
| 100 | P00057 Wnt signaling pathway | 0.0111685 | 12 |
| 101 | WP2037 Prolactin signaling pathway | 0.0111685 | 11 |
| 102 | WP22 IL-9 signaling pathway | 0.0111685 | 6 |
| 103 | WP410 Diurnally regulated genes with circadian orthologs | 0.0111685 | 8 |
| 104 | WP45 G1 to S-cell cycle control | 0.0111685 | 12 |
| 105 | hsa00100 Steroid biosynthesis | 0.0111685 | 5 |
| 106 | hsa00532 Chondroitin sulfate biosynthesis | 0.0111685 | 5 |
| 107 | hsa04110 Cell cycle | 0.0111685 | 13 |
| 108 | hsa04670 Leukocyte transendothelial migration | 0.0111685 | 10 |
| 109 | hsa05212 Pancreatic cancer | 0.0111685 | 12 |
| 110 | WP2034 Leptin signaling pathway | 0.0115687 | 11 |
| 111 | WP314 FAS pathway and stress induction of HSP regulation | 0.0115687 | 9 |
| 112 | hsa01040 Biosynthesis of unsaturated fatty acids | 0.0115687 | 6 |
| 113 | hsa04330 Notch signaling pathway | 0.0115687 | 8 |
| 114 | hsa04510 Focal adhesion | 0.0115687 | 13 |
| 115 | hsa04810 Regulation of actin cytoskeleton | 0.0115687 | 11 |
| 116 | hsa00290 Valine leucine and isoleucine biosynthesis | 0.0117869 | 5 |
| 117 | WP231 TNF alpha signaling pathway | 0.0118601 | 10 |
| 118 | hsa04062 Chemokine signaling pathway | 0.011884 | 11 |
| 119 | P02752 Mannose metabolism | 0.0120436 | 3 |
| 120 | WP545 Complement activation classical pathway | 0.0120436 | 3 |
| 121 | hsa00561 Glycerolipid metabolism | 0.0120436 | 6 |
| 122 | hsa04530 Tight junction | 0.012111 | 11 |
| 123 | P00001 Adrenaline and noradrenaline biosynthesis | 0.0122911 | 4 |
| 124 | WP244 Alpha 6 beta 4 signaling pathway | 0.0122911 | 8 |
| 125 | WP254 Apoptosis | 0.0122911 | 11 |
| 126 | WP28 Selenium metabolism and selenoproteins | 0.0122911 | 6 |
| 127 | hsa00450 Selenoamino acid metabolism | 0.0122911 | 5 |
| 128 | hsa01100 Metabolic pathways | 0.0122911 | 13 |
| 129 | hsa05214 Glioma | 0.0122911 | 12 |
| 130 | P00033 Insulin IGF pathway protein kinase B signaling cascade | 0.0127806 | 9 |
| 131 | WP1601 Fluoropyrimidine activity | 0.0127806 | 7 |
| 132 | WP49 IL 2 Signaling pathway | 0.0127806 | 9 |
| 133 | P00054 Toll receptor signaling pathway | 0.0132828 | 8 |
| 134 | P02745 Glutamine glutamate conversion | 0.0132828 | 4 |
| 135 | hsa00562 Inositol phosphate metabolism | 0.0132828 | 8 |
| 136 | hsa04120 Ubiquitin-mediated proteolysis | 0.0132828 | 10 |
| 137 | hsa04710 Circadian rhythm mammal | 0.0132828 | 4 |
| 138 | hsa04916 Melanogenesis | 0.0132828 | 9 |
| 139 | hsa03010 Ribosome | 0.013922 | 10 |
| 140 | P00038 JAK/STAT signaling pathway | 0.0139829 | 5 |
| 141 | P00003 Alzheimer disease amyloid secretase pathway | 0.0143043 | 8 |
| 142 | WP1591 Heart development | 0.0143043 | 9 |
| 143 | WP411 mRNA processing | 0.0143043 | 10 |
| 144 | hsa04612 Antigen processing and presentation | 0.0143043 | 8 |
| 145 | hsa05110 Vibrio cholerae infection | 0.0143043 | 8 |
| 146 | P00004 Alzheimer disease presenilin pathway | 0.0143586 | 10 |
| 147 | WP272 Blood clotting cascade | 0.0143586 | 4 |
| 148 | WP481 Insulin signaling | 0.0143586 | 11 |
| 149 | hsa04650 Natural killer cell mediated cytotoxicity | 0.0143586 | 9 |
| 150 | hsa05200 Pathways in cancer | 0.0143586 | 14 |
| 151 | hsa00600 Sphingolipid metabolism | 0.0145061 | 5 |
| 152 | P00046 Oxidative stress response | 0.0145322 | 8 |
| 153 | P00055 Transcription regulation by bZIP transcription factor | 0.0145322 | 7 |
| 154 | WP581 EPO receptor signaling | 0.0145322 | 7 |
| 155 | hsa04912 GnRH signaling pathway | 0.0145322 | 9 |
| 156 | hsa05130 Pathogenic Escherichia coli infection | 0.0145322 | 9 |
| 157 | P00006 Apoptosis signaling pathway | 0.0147027 | 11 |
| 158 | P00017 DNA replication | 0.0147027 | 6 |
| 159 | P00022 General transcription by RNA polymerase I | 0.0147027 | 4 |
| 160 | P00034 Integrin signaling pathway | 0.0147027 | 11 |
| 161 | P04376 5HT4 type receptor-mediated signaling pathway | 0.0147027 | 5 |
| 162 | P04379 Beta3 adrenergic receptor signaling pathway | 0.0147027 | 5 |
| 163 | WP106 Alanine and aspartate metabolism | 0.0147027 | 3 |
| 164 | WP710 DNA damage response only ATM-dependent | 0.0147027 | 12 |
| 165 | WP78 TCA Cycle | 0.0147027 | 5 |
| 166 | hsa00120 Primary bile acid biosynthesis | 0.0147027 | 3 |
| 167 | hsa00564 Glycerophospholipid metabolism | 0.0147027 | 6 |
| 168 | hsa04930 Type II diabetes mellitus | 0.0147027 | 8 |
| 169 | hsa05220 Chronic myeloid leukemia | 0.0147027 | 12 |
| 170 | WP286 IL-3 signaling pathway | 0.0149452 | 9 |
| 171 | P00021 FGF signaling pathway | 0.0150871 | 10 |
| 172 | hsa00190 Oxidative phosphorylation | 0.0150871 | 8 |
| 173 | hsa00250 Alanine aspartate and glutamate metabolism | 0.0150871 | 6 |
| 174 | hsa00860 Porphyrin and chlorophyll metabolism | 0.0150871 | 5 |
| 175 | hsa00903 Limonene and pinene degradation | 0.0150871 | 5 |
| 176 | hsa03050 Proteasome | 0.0150871 | 7 |
| 177 | hsa04150 mTOR signaling pathway | 0.0150871 | 9 |
| 178 | hsa04730 Long-term depression | 0.0150871 | 8 |
| 179 | hsa04740 Olfactory transduction | 0.0150871 | 5 |
| 180 | hsa04914 Progesterone-mediated oocyte maturation | 0.0150871 | 10 |
| 181 | WP1984 Integrated breast cancer pathway | 0.0154271 | 10 |
| 182 | hsa04540 Gap junction | 0.0155233 | 9 |
| 183 | hsa04620 Toll-like receptor signaling pathway | 0.0155233 | 9 |
| 184 | P04380 Cortocotropin-releasing factor receptor signaling pathway | 0.0162701 | 5 |
| 185 | WP75 Toll-like receptor signaling | 0.0162701 | 9 |
| 186 | P02756 N acetylglucosamine metabolism | 0.0163611 | 3 |
| 187 | P00016 Cytoskeletal regulation by Rho GTPase | 0.0169474 | 9 |
| 188 | P00027 Heterotrimeric G protein signaling pathway Gq alpha and Go alpha mediated pathway | 0.017088 | 7 |
| 189 | hsa04622 RIG I-like receptor signaling pathway | 0.017088 | 7 |
| 190 | WP47 Hedgehog signaling pathway | 0.0172104 | 4 |
| 191 | hsa04130 SNARE interactions in vesicular transport | 0.0174168 | 5 |
| 192 | WP179 Cell cycle | 0.0175914 | 12 |
| 193 | hsa04070 Phosphatidylinositol signaling system | 0.0175914 | 8 |
| 194 | hsa04621 NOD-like receptor signaling pathway | 0.0175914 | 8 |
| 195 | hsa04320 Dorso ventral axis formation | 0.0177216 | 7 |
| 196 | hsa05213 Endometrial cancer | 0.0182709 | 10 |
| 197 | hsa04142 Lysosome | 0.0183738 | 8 |
| 198 | P00056 VEGF signaling pathway | 0.0183988 | 9 |
| 199 | P02774 Salvage pyrimidine deoxyribonucleotides | 0.0183988 | 2 |
| 200 | WP437 EGF–EGFR signaling pathway | 0.0183988 | 11 |
| 201 | WP550 Biogenic amine synthesis | 0.0183988 | 2 |
| 202 | hsa00603 Glycosphingolipid biosynthesis globo series | 0.0183988 | 3 |
| 203 | hsa03060 Protein export | 0.0183988 | 5 |
| 204 | WP23 B-cell receptor signaling pathway | 0.0184271 | 10 |
| 205 | WP35 G protein signaling pathways | 0.018623 | 8 |
| 206 | hsa05014 Amyotrophic lateral sclerosis (ALS) | 0.018623 | 8 |
| 207 | hsa04115 p53 signaling pathway | 0.0187125 | 11 |
| 208 | hsa04520 Adherens junction | 0.0187125 | 11 |
| 209 | hsa03018 RNA degradation | 0.018873 | 7 |
| 210 | WP405 Eukaryotic transcription initiation | 0.0191055 | 6 |
| 211 | hsa04146 Peroxisome | 0.0191055 | 6 |
| 212 | P00037 Ionotropic glutamate receptor pathway | 0.019905 | 4 |
| 213 | P00036 Interleukin signaling pathway | 0.0212554 | 10 |
| 214 | WP127 IL-5 signaling pathway | 0.0212554 | 8 |
| 215 | WP560 TGF beta signaling pathway2 | 0.0212559 | 9 |
| 216 | P00059 p53 pathway | 0.0215208 | 11 |
| 217 | P00029 Huntington disease | 0.0218464 | 10 |
| 218 | P05730 Endogenous cannabinoid signaling | 0.0218464 | 4 |
| 219 | hsa05012 Parkinsons disease | 0.0218541 | 8 |
| 220 | hsa05215 Prostate cancer | 0.0218541 | 12 |
| 221 | P00020 FAS signaling pathway | 0.0229873 | 7 |
| 222 | P00049 Parkinson disease | 0.0235854 | 9 |
| 223 | P02742 Tetrahydrofolate biosynthesis | 0.0235854 | 3 |
| 224 | WP334 GPCRs class B secretin-like | 0.0235854 | 3 |
| 225 | WP357 Fatty acid biosynthesis | 0.0235854 | 6 |
| 226 | WP474 Endochondral ossification | 0.0235854 | 9 |
| 227 | hsa00340 Histidine metabolism | 0.0235854 | 4 |
| 228 | hsa00760 Nicotinate and nicotinamide metabolism | 0.0235854 | 4 |
| 229 | hsa03020 RNA polymerase | 0.0235854 | 5 |
| 230 | hsa04910 Insulin signaling pathway | 0.0236514 | 10 |
| 231 | hsa05223 Non-small cell lung cancer | 0.0236514 | 10 |
| 232 | P05918 p38 MAPK pathway | 0.0236516 | 2 |
| 233 | P00025 Hedgehog signaling pathway | 0.0243519 | 5 |
| 234 | WP453 Inflammatory response pathway | 0.0243519 | 5 |
| 235 | WP558 Complement and coagulation cascades | 0.0243519 | 5 |
| 236 | hsa04610 Complement and coagulation cascades | 0.0243519 | 5 |
| 237 | P00048 PI3 kinase pathway | 0.0246711 | 9 |
| 238 | hsa00230 Purine metabolism | 0.0250551 | 8 |
| 239 | WP185 Integrin-mediated cell adhesion | 0.0256642 | 9 |
| 240 | P04377 Beta1 adrenergic receptor signaling pathway | 0.0257021 | 5 |
| 241 | P04378 Beta2 adrenergic receptor signaling pathway | 0.0257021 | 5 |
| 242 | WP422 MAPK cascade | 0.0257021 | 6 |
| 243 | hsa00330 Arginine and proline metabolism | 0.0257021 | 6 |
| 244 | hsa00680 Methane metabolism | 0.0259845 | 3 |
| 245 | WP111 Electron transport chain | 0.0263266 | 7 |
| 246 | hsa05211 Renal cell carcinoma | 0.0265335 | 10 |
| 247 | WP623 Oxidative phosphorylation | 0.0266553 | 6 |
| 248 | hsa00512 O-glycan biosynthesis | 0.0266553 | 4 |
| 249 | hsa03450 Non-homologous end joining | 0.0266553 | 4 |
| 250 | WP2256 Integrated pancreatic cancer pathway | 0.0276038 | 12 |
| 251 | hsa04662 B-cell receptor signaling pathway | 0.0277421 | 8 |
| 252 | WP69 TCR signaling pathway | 0.0280222 | 9 |
| 253 | P05731 GABA B receptor II signaling | 0.0288666 | 5 |
| 254 | WP430 Statin pathway | 0.0288666 | 5 |
| 255 | hsa04210 Apoptosis | 0.028998 | 9 |
| 256 | WP496 Steroid biosynthesis | 0.0293735 | 2 |
| 257 | hsa00471 D glutamine and D glutamate metabolism | 0.0293735 | 2 |
| 258 | hsa04920 Adipocytokine signaling pathway | 0.0298415 | 8 |
| 259 | hsa00480 Glutathione metabolism | 0.0305859 | 5 |
| 260 | P00041 Metabotropic glutamate receptor group I pathway | 0.0309001 | 4 |
| 261 | WP236 Adipogenesis | 0.0309001 | 10 |
| 262 | WP673 ErbB signaling pathway | 0.0309001 | 9 |
| 263 | hsa05020 Prion diseases | 0.0309001 | 6 |
| 264 | P00010 B-cell activation | 0.0317596 | 7 |
| 265 | WP100 Glutathione metabolism | 0.0317596 | 3 |
| 266 | WP734 Serotonin receptor 4 6 7 and NR3C signaling | 0.0317596 | 5 |
| 267 | hsa00650 Butanoate metabolism | 0.0317596 | 5 |
| 268 | hsa00740 Riboflavin metabolism | 0.0317596 | 3 |
| 269 | hsa05010 Alzheimers disease | 0.0317596 | 9 |
| 270 | WP732 Serotonin receptor 2 and ELK SRF GATA4 signaling | 0.0331337 | 4 |
| 271 | P00031 Inflammation mediated by chemokine and cytokine signaling pathway | 0.0338017 | 10 |
| 272 | P00012 Cadherin signaling pathway | 0.0356967 | 8 |
| 273 | P02776 Serine glycine biosynthesis | 0.0356967 | 3 |
| 274 | hsa00410 beta alanine metabolism | 0.0356967 | 4 |
| 275 | hsa00980 Metabolism of xenobiotics by cytochrome P450 | 0.0356967 | 4 |
| 276 | WP311 Synthesis and degradation of ketone bodies | 0.0358374 | 2 |
| 277 | hsa05219 Bladder cancer | 0.0358725 | 10 |
| 278 | WP706 SIDS Susceptibility pathways | 0.0359922 | 7 |
| 279 | P04397 p53 pathway by glucose deprivation | 0.0368786 | 6 |
| 280 | P00047 PDGF signaling pathway | 0.0373517 | 9 |
| 281 | P04394 Thyrotropin-releasing hormone receptor signaling pathway | 0.0373517 | 5 |
| 282 | hsa00020 Citrate cycle TCA cycle | 0.0373517 | 5 |
| 283 | hsa00280 Valine leucine and isoleucine degradation | 0.0373517 | 5 |
| 284 | hsa04270 Vascular smooth muscle contraction | 0.0373517 | 7 |
| 285 | WP2018 RANKL RANK signaling pathway | 0.0377374 | 8 |
| 286 | WP1545 miRNAs involved in DDR | 0.0383983 | 9 |
| 287 | P04395 Vasopressin synthesis | 0.038477 | 3 |
| 288 | hsa00790 Folate biosynthesis | 0.038477 | 3 |
| 289 | hsa05410 Hypertrophic cardiomyopathy (HCM) | 0.038477 | 7 |
| 290 | P00039 Metabotropic glutamate receptor group III pathway | 0.0389214 | 5 |
| 291 | P04391 Oxytocin receptor mediated signaling pathway | 0.0389214 | 5 |
| 292 | hsa04080 Neuroactive ligand receptor interaction | 0.0389214 | 5 |
| 293 | WP2059 Alzheimers disease | 0.040197 | 8 |
| 294 | P04374 5HT2 type receptor mediated signaling pathway | 0.0413535 | 5 |
| 295 | WP176 Folate metabolism | 0.041381 | 7 |
| 296 | hsa05414 Dilated cardiomyopathy | 0.041381 | 7 |
| 297 | P00018 EGF receptor signaling pathway | 0.0418825 | 9 |
| 298 | hsa00533 Keratan sulfate biosynthesis | 0.0421327 | 3 |
| 299 | P00028 Heterotrimeric G protein signaling pathway rod outer segment phototransduction | 0.0424821 | 4 |
| 300 | WP1559 TFs regulate miRNAs related to cardiac hypertrophy | 0.0424821 | 4 |
| 301 | WP2118 Arrhythmogenic right ventricular cardiomyopathy | 0.0425917 | 7 |
| 302 | hsa05412 Arrhythmogenic right ventricular cardiomyopathy ARVC | 0.0425917 | 7 |
| 303 | P00040 Metabotropic glutamate receptor group II pathway | 0.042862 | 5 |
| 304 | hsa05222 Small cell lung cancer | 0.0430192 | 10 |
| 305 | WP313 Signaling of hepatocyte growth factor receptor | 0.0442582 | 7 |
| 306 | P00005 Angiogenesis | 0.0442601 | 10 |
| 307 | P02739 De novo pyrimidine deoxyribonucleotide biosynthesis | 0.0442601 | 4 |
| 308 | P04385 Histamine H1 receptor mediated signaling pathway | 0.0442601 | 5 |
| 309 | P04392 P53 pathway feedback loops 1 | 0.0442601 | 4 |
| 310 | WP138 Androgen receptor signaling pathway | 0.0442601 | 11 |
| 311 | WP455 GPCRs class A rhodopsin-like | 0.0442601 | 4 |
| 312 | WP530 Cytokines and inflammatory response | 0.0442601 | 4 |
| 313 | hsa00640 Propanoate metabolism | 0.0442601 | 5 |
| 314 | WP438 Non-homologous end joining | 0.0447252 | 3 |
| 315 | hsa00531 Glycosaminoglycan degradation | 0.0447252 | 3 |
| 316 | WP183 Proteasome degradation | 0.0448018 | 7 |
| 317 | WP268 Notch signaling pathway | 0.0454571 | 6 |
| 318 | hsa04666 FC gamma R mediated phagocytosis | 0.046084 | 8 |
| 319 | P00026 Heterotrimeric G protein signaling pathway Gi alpha and Gs alpha mediated pathway | 0.0463716 | 7 |
| 320 | WP712 Estrogen signaling pathway | 0.0463716 | 7 |
| 321 | P02759 Pyridoxal 5 phosphate biosynthesis | 0.0473821 | 2 |
| 322 | P02788 Xanthine and guanine salvage pathway | 0.0473821 | 2 |
| 323 | P05726 2 arachidonoylglycerol biosynthesis | 0.0473821 | 2 |
| 324 | WP690 Polyol pathway | 0.0473821 | 2 |
| 325 | P00042 Muscarinic acetylcholine receptor 1 and 3 signaling pathway | 0.0483055 | 5 |
| 326 | P04386 Histamine H2 receptor mediated signaling pathway | 0.0495852 | 4 |

**Table S5.** Statistically significant records, based on both raw and empirical p values, provided by enrichment analysis by miRSystem for 15 core miRNAs. Results were sorted by empirical p value. ‘PID’ indicates – Pathway Interaction Database.

| **Category** | **Term** | **Union miRNAs** | **Raw p value** | **Empirical p value** |
| --- | --- | --- | --- | --- |
| REACTOME | GOLGI_ASSOCIATED_VESICLE_BIOGENESIS | 5 | 1.4e-2 | 9.93e-3 |
| REACTOME | CLATHRIN_DERIVED_VESICLE_BUDDING | 5 | 1.76e-2 | 9.93e-3 |
| REACTOME | TRANS-GOLGI_NETWORK_VESICLE_BUDDING | 5 | 1.76e-2 | 9.93e-3 |
| REACTOME | MEMBRANE_TRAFFICKING | 5 | 9.83e-3 | 1.58e-2 |
| BIOCARTA | BIOCARTA_AGR_PATHWAY | 3 | 6.61e-3 | 1.76e-2 |
| PID | SIGNALING_EVENTS_MEDIATED_BY_ FOCAL_ADHESION_KINASE | 5 | 1.61e-2 | 2.41e-2 |
| KEGG | ANTIGEN_PROCESSING_AND_ PRESENTATION | 4 | 2.61e-2 | 3.31e-2 |

**Table S6.** Top 3 KEGG pathway records obtained for core miRNAs (DIANA-miRPath, genes union).

| **miRNA** | **KEGG pathway** | **rounded p value** | **#genes** |
| --- | --- | --- | --- |
| **mir-140-5p** | Hippo signaling pathway (hsa04390) | 0.00010 | 7 |
|  | ECM–receptor interaction (hsa04512) | 0.00013 | 19 |
|  | p53 signaling pathway (hsa04115) | 0.00127 | 11 |
| **mir-31-3p** | Glycosaminoglycan biosynthesis: keratan sulfate (hsa00533) | 0.00688 | 1 |
|  | Other types of O-glycan biosynthesis (hsa00514) | 0.02912 | 1 |
| **miR-151a-5p** | Arrhythmogenic right ventricular cardiomyopathy (ARVC) (hsa05412) | 1.4e-07 | 4 |
|  | Proteoglycans in cancer (hsa05205) | 0.00087 | 8 |
|  | MicroRNAs in cancer (hsa05206) | 0.00368 | 11 |
| **mir-93-5p** | Prion diseases (hsa05020) | 4.3e-19 | 5 |
|  | mRNA surveillance pathway (hsa03015) | 1.7e-05 | 27 |
|  | TGF-beta signaling pathway (hsa04350) | 1.9e-05 | 19 |
| **mir-106b-5p** | Prion diseases (hsa05020) | 1.7e-21 | 5 |
|  | Protein processing in endoplasmic reticulum (hsa04141) | 1.9e-06 | 39 |
|  | Cell cycle (hsa04110) | 1.7e-05 | 30 |
| **mir-193a-5p** | Steroid biosynthesis (hsa00100) | 5.5e-07 | 1 |
|  | ECM–receptor interaction (hsa04512) | 1.6e-05 | 2 |
| **mir-193b-3p** | Prion diseases (hsa05020) | 1.4e-22 | 3 |
|  | Fatty acid biosynthesis (hsa00061) | 5.7e-20 | 1 |
|  | Fatty acid metabolism (hsa01212) | 4.2e-14 | 5 |
| **mir-484** | Fatty acid biosynthesis (hsa00061) | 6.6e-24 | 1 |
|  | ECM–receptor interaction (hsa04512) | 4.2e-09 | 9 |
|  | Glycosaminoglycan biosynthesis: chondroitin sulfate / dermatan sulfate (hsa00532) | 1.1e-05 | 4 |
| **mir-376c-3p** | Biosynthesis of unsaturated fatty acids (hsa01040) | 2.1e-05 | 1 |
|  | ECM–receptor interaction (hsa04512) | 0.00279 | 4 |
|  | Sulfur metabolism (hsa00920) | 0.00941 | 1 |
| **miR-26b-5p** | Glycosaminoglycan biosynthesis: chondroitin sulfate / dermatan sulfate (hsa00532) | 4.8e-07 | 9 |
|  | Hippo signaling pathway (hsa04390) | 7.3e-05 | 31 |
|  | p53 signaling pathway (hsa04115) | 0.00015 | 27 |
| **mir-93-3p** | Fatty acid biosynthesis (hsa00061) | 8.2e-28 | 1 |
|  | Fatty acid metabolism (hsa01212) | 8.2e-09 | 3 |
|  | RNA transport (hsa03013) | 0.0030 | 18 |
| **mir-29a-3p** | ECM–receptor interaction (hsa04512) | 1.3e-54 | 21 |
|  | Fatty acid biosynthesis (hsa00061) | 8.3e-19 | 2 |
|  | Viral carcinogenesis (hsa05203) | 1.2e-10 | 42 |
| **mir-374b-5p** | p53 signaling pathway (hsa04115) | 1.3e-05 | 16 |
|  | Oocyte meiosis (hsa04114) | 0.00357 | 11 |
|  | Proteoglycans in cancer (hsa05205) | 0.00487 | 19 |
| **mir-16-5p** | Prion diseases (hsa05020) | 7.6e-14 | 10 |
|  | Fatty acid biosynthesis (hsa00061) | 7.7e-11 | 4 |
|  | Viral carcinogenesis (hsa05203) | 4.0e-09 | 69 |
| **let-7g-5p** | ECM–receptor interaction (hsa04512) | 1.6e-07 | 12 |
|  | Viral carcinogenesis (hsa05203) | 5.1e-06 | 39 |
|  | Cell cycle (hsa04110) | 6.4e-06 | 32 |

**Table S7.** KEGG pathways found to be enriched for 11 significantly changed miRNAs in HHH vs. NNN, generated by DIANA-miRPath v3 and merged by genes union.

| KEGG pathway | p-value | #genes | #miRNAs |
| --- | --- | --- | --- |
| Proteoglycans in cancer (hsa05205) | 2.27E-12 | 144 | 11 |
| Cell cycle (hsa04110) | 2.19E-09 | 95 | 11 |
| Viral carcinogenesis (hsa05203) | 3.15E-08 | 139 | 11 |
| Endocytosis (hsa04144) | 1.24E-07 | 147 | 11 |
| Hepatitis B (hsa05161) | 1.79E-07 | 101 | 11 |
| Colorectal cancer (hsa05210) | 2.86E-07 | 51 | 11 |
| Adherens junction (hsa04520) | 6.91E-07 | 55 | 11 |
| Hippo signaling pathway (hsa04390) | 7.69E-07 | 97 | 11 |
| Transcriptional misregulation in cancer (hsa05202) | 1.29E-06 | 122 | 11 |
| p53 signaling pathway (hsa04115) | 1.92E-06 | 56 | 11 |
| Protein processing in endoplasmic reticulum (hsa04141) | 1.92E-06 | 114 | 11 |
| Pathways in cancer (hsa05200) | 3.98E-06 | 249 | 11 |
| TGF-beta signaling pathway (hsa04350) | 5.03E-06 | 56 | 11 |
| Renal cell carcinoma (hsa05211) | 1.08E-05 | 52 | 11 |
| Chronic myeloid leukemia (hsa05220) | 1.17E-05 | 56 | 11 |
| Thyroid hormone signaling pathway (hsa04919) | 2.53E-05 | 81 | 11 |
| Glycosaminoglycan biosynthesis: keratan sulfate (hsa00533) | 6.86E-05 | 13 | 7 |
| Pancreatic cancer (hsa05212) | 0.000131 | 51 | 11 |
| Prostate cancer (hsa05215) | 0.000235 | 66 | 11 |
| Prion diseases (hsa05020) | 0.000243 | 18 | 9 |
| HTLV-I infection (hsa05166) | 0.000373 | 163 | 11 |
| Focal adhesion (hsa04510) | 0.000391 | 135 | 11 |
| Lysine degradation (hsa00310) | 0.000574 | 31 | 11 |
| MAPK signaling pathway (hsa04010) | 0.000578 | 160 | 11 |
| Thyroid cancer (hsa05216) | 0.000627 | 24 | 11 |
| Small cell lung cancer (hsa05222) | 0.001019 | 61 | 11 |
| Non-small cell lung cancer (hsa05223) | 0.001607 | 40 | 11 |
| Regulation of actin cytoskeleton (hsa04810) | 0.001704 | 134 | 11 |
| Endometrial cancer (hsa05213) | 0.001936 | 38 | 11 |
| FOXO signaling pathway (hsa04068) | 0.00353 | 90 | 11 |
| Bacterial invasion of epithelial cells (hsa05100) | 0.003904 | 54 | 11 |
| DNA replication (hsa03030) | 0.003983 | 26 | 7 |
| Oocyte meiosis (hsa04114) | 0.003983 | 70 | 11 |
| Melanoma (hsa05218) | 0.004104 | 48 | 11 |
| HIF-1 signaling pathway (hsa04066) | 0.004533 | 70 | 11 |
| Ubiquitin-mediated proteolysis (hsa04120) | 0.004533 | 92 | 11 |
| Neurotrophin signaling pathway (hsa04722) | 0.004533 | 77 | 11 |
| Glioma (hsa05214) | 0.004533 | 43 | 11 |
| mTOR signaling pathway (hsa04150) | 0.007766 | 43 | 11 |
| Fatty acid metabolism (hsa01212) | 0.008401 | 31 | 9 |
| Arrhythmogenic right ventricular cardiomyopathy (ARVC) (hsa05412) | 0.009588 | 40 | 11 |
| Fatty acid biosynthesis (hsa00061) | 0.011441 | 7 | 9 |
| Acute myeloid leukemia (hsa05221) | 0.01189 | 40 | 10 |
| Axon guidance (hsa04360) | 0.014089 | 75 | 11 |
| Epstein–Barr virus infection (hsa05169) | 0.022427 | 126 | 11 |
| Progesterone-mediated oocyte maturation (hsa04914) | 0.022598 | 58 | 11 |
| Ras signaling pathway (hsa04014) | 0.023908 | 131 | 11 |
| TNF signaling pathway (hsa04668) | 0.023908 | 69 | 11 |
| Insulin signaling pathway (hsa04910) | 0.024402 | 88 | 11 |
| Central carbon metabolism in cancer (hsa05230) | 0.024402 | 44 | 11 |
| Bladder cancer (hsa05219) | 0.025115 | 28 | 11 |
| Mismatch repair (hsa03430) | 0.02936 | 17 | 7 |
| Chagas disease (American trypanosomiasis) (hsa05142) | 0.034415 | 63 | 11 |
| Glycosaminoglycan biosynthesis: heparan sulfate / heparin (hsa00534) | 0.035129 | 15 | 8 |
| Homologous recombination (hsa03440) | 0.039609 | 20 | 8 |
| Glycosaminoglycan biosynthesis: chondroitin sulfate / dermatan sulfate (hsa00532) | 0.044732 | 13 | 5 |
| N-glycan biosynthesis (hsa00510) | 0.045985 | 34 | 9 |

**Table S8.** KEGG pathways found to be enriched for 11 significantly changed miRNAs in HHH vs. NNN, generated by DIANA-miRPath v3 and merged by pathways union.

| KEGG pathway | p-value | #genes | #miRNAs |
| --- | --- | --- | --- |
| Prion diseases (hsa05020) | <1e-325 | 3 | 2 |
| Fatty acid biosynthesis (hsa00061) | <1e-325 | 5 | 4 |
| Cell cycle (hsa04110) | 9,99E-16 | 87 | 7 |
| Proteoglycans in cancer (hsa05205) | 8,55E-15 | 136 | 8 |
| Hippo signaling pathway (hsa04390) | 1,55E-14 | 74 | 6 |
| Viral carcinogenesis (hsa05203) | 2,31E-14 | 127 | 6 |
| p53 signaling pathway (hsa04115) | 6,24E-13 | 55 | 6 |
| Adherens junction (hsa04520) | 1,58E-10 | 53 | 6 |
| Fatty acid metabolism (hsa01212) | 4,44E-10 | 28 | 5 |
| ECM–receptor interaction (hsa04512) | 5,78E-09 | 21 | 3 |
| Protein processing in endoplasmic reticulum (hsa04141) | 2,08E-08 | 85 | 4 |
| Pathways in cancer (hsa05200) | 2,26E-08 | 219 | 6 |
| Chronic myeloid leukemia (hsa05220) | 2,29E-08 | 50 | 6 |
| Colorectal cancer (hsa05210) | 1,70E-07 | 48 | 6 |
| Hepatitis B (hsa05161) | 4,57E-07 | 91 | 4 |
| Lysine degradation (hsa00310) | 1,09E-06 | 24 | 4 |
| Thyroid hormone signaling pathway (hsa04919) | 1,46E-06 | 71 | 4 |
| Glioma (hsa05214) | 1,49E-06 | 36 | 3 |
| Arrhythmogenic right ventricular cardiomyopathy (ARVC) (hsa05412) | 3,80E-06 | 23 | 4 |
| TGF-beta signaling pathway (hsa04350) | 6,48E-05 | 47 | 6 |
| Glycosaminoglycan biosynthesis: keratan sulfate (hsa00533) | 9,09E-05 | 10 | 3 |
| Bladder cancer (hsa05219) | 9,13E-05 | 27 | 3 |
| Pancreatic cancer (hsa05212) | 0,0002 | 45 | 3 |
| Oocyte meiosis (hsa04114) | 0,0005 | 47 | 3 |
| Melanoma (hsa05218) | 0,0010 | 35 | 2 |
| Prostate cancer (hsa05215) | 0,0013 | 56 | 4 |
| Renal cell carcinoma (hsa05211) | 0,0014 | 32 | 3 |
| Signaling pathways regulating pluripotency of stem cells (hsa04550) | 0,0019 | 35 | 2 |
| HTLV-I infection (hsa05166) | 0,0019 | 134 | 4 |
| FOXO signaling pathway (hsa04068) | 0,0024 | 38 | 2 |
| Bacterial invasion of epithelial cells (hsa05100) | 0,0032 | 45 | 4 |
| Regulation of actin cytoskeleton (hsa04810) | 0,0033 | 86 | 3 |
| Small cell lung cancer (hsa05222) | 0,0041 | 48 | 3 |
| Other types of O-glycan biosynthesis (hsa00514) | 0,0061 | 14 | 3 |
| Transcriptional misregulation in cancer (hsa05202) | 0,0066 | 85 | 3 |
| Focal adhesion (hsa04510) | 0,0076 | 65 | 3 |
| Endocytosis (hsa04144) | 0,0077 | 94 | 2 |
| Central carbon metabolism in cancer (hsa05230) | 0,0096 | 32 | 2 |
| Non-small cell lung cancer (hsa05223) | 0,0162 | 30 | 2 |
| Vitamin B6 metabolism (hsa00750) | 0,0208 | 4 | 2 |
| Endometrial cancer (hsa05213) | 0,0209 | 29 | 3 |
| PI3K-Akt signaling pathway (hsa04151) | 0,0229 | 73 | 2 |
| Glycosaminoglycan biosynthesis: chondroitin sulfate / dermatan sulfate (hsa00532) | 0,0345 | 9 | 2 |
| Epstein–Barr virus infection (hsa05169) | 0,0426 | 60 | 2 |

**Table S9.** Overrepresentation analysis performed by miEAA for 11 significantly changed miRNAs in HHH vs. NNN with statistically significant records sorted by p value. SIDS - Sudden Infant Death Syndrome, NLR – Nod-like receptor.

| Rank | Subcategory | p value | #miRNAs |
| --- | --- | --- | --- |
| 1. | hsa04612 Antigen processing and presentation | 0.000396551 | 10 |
| 2. | WP241 One carbon metabolism | 0.00077458 | 8 |
| 3. | hsa04623 Cytosolic DNA sensing pathway | 0.00077458 | 8 |
| 4. | hsa05416 Viral myocarditis | 0.00105189 | 10 |
| 5. | WP357 Fatty acid biosynthesis | 0.00121643 | 8 |
| 6. | WP1544 MicroRNAs in cardiomyocyte hypertrophy | 0.00158279 | 10 |
| 7. | P00049 Parkinson disease | 0.00178193 | 10 |
| 8. | hsa00100 Steroid biosynthesis | 0.00178193 | 6 |
| 9. | hsa04144 Endocytosis | 0.00178193 | 11 |
| 10. | P00038 JAK/STAT signaling pathway | 0.00184187 | 6 |
| 11. | WP1559 TFs regulate miRNAs related to cardiac hypertrophy | 0.00184187 | 6 |
| 12. | WP1984 Integrated breast cancer pathway | 0.00184187 | 10 |
| 13. | WP404 Nucleotide metabolism | 0.00184187 | 7 |
| 14. | hsa00903 Limonene and pinene degradation | 0.00184187 | 6 |
| 15. | hsa03040 Spliceosome | 0.00184187 | 10 |
| 16. | hsa04020 Calcium signaling pathway | 0.00184187 | 9 |
| 17. | hsa04060 Cytokine cytokine receptor interaction | 0.00184187 | 10 |
| 18. | hsa04350 TGF beta signaling pathway | 0.00184187 | 10 |
| 19. | hsa04514 Cell adhesion molecules CAMs | 0.00184187 | 8 |
| 20. | hsa04622 RIG I-like receptor signaling pathway | 0.00184187 | 8 |
| 21. | hsa04630 JAK/STAT signaling pathway | 0.00184187 | 10 |
| 22. | hsa04722 Neurotrophin signaling pathway | 0.00184187 | 11 |
| 23. | hsa04960 Aldosterone regulated sodium reabsorption | 0.00184187 | 8 |
| 24. | hsa05340 Primary immunodeficiency | 0.00184187 | 5 |
| 25. | P02738 De novo purine biosynthesis | 0.00224208 | 7 |
| 26. | WP395 IL-4 signaling pathway | 0.00224208 | 9 |
| 27. | WP400 p38 MAPK signaling pathway | 0.0031675 | 8 |
| 28. | hsa04540 Gap junction | 0.0031675 | 9 |
| 29. | WP623 Oxidative phosphorylation | 0.00355176 | 7 |
| 30. | hsa03020 RNA polymerase | 0.00355176 | 6 |
| 31. | hsa03440 Homologous recombination | 0.00355176 | 6 |
| 32. | P02723 Adenine and hypoxanthine salvage pathway | 0.00377707 | 4 |
| 33. | WP12 Osteoclast Signaling | 0.00379448 | 5 |
| 34. | WP197 Cholesterol Biosynthesis | 0.00379448 | 5 |
| 35. | WP262 EBV LMP1 signaling | 0.00379448 | 7 |
| 36. | WP560 TGF beta signaling pathway 2 | 0.00379448 | 9 |
| 37. | hsa00270 Cysteine and methionine metabolism | 0.00379448 | 7 |
| 38. | hsa03430 Mismatch repair | 0.00379448 | 6 |
| 39. | hsa04310 Wnt signaling pathway | 0.00379448 | 10 |
| 40. | hsa05020 Prion diseases | 0.00379448 | 7 |
| 41. | WP702 Metapathway biotransformation | 0.00381781 | 7 |
| 42. | hsa00534 Heparan sulfate biosynthesis | 0.0039779 | 5 |
| 43. | hsa00563 Glycosylphosphatidylinositol (GPI) anchor biosynthesis | 0.0039779 | 5 |
| 44. | P05911 Angiotensin II stimulated signaling through G proteins and beta arrestin | 0.00399368 | 2 |
| 45. | WP1600 Nicotine metabolism | 0.00399368 | 2 |
| 46. | WP183 Proteasome degradation | 0.00399368 | 8 |
| 47. | WP185 Integrin mediated cell adhesion | 0.00399368 | 9 |
| 48. | WP231 TNF alpha signaling pathway | 0.00399368 | 9 |
| 49. | WP474 Endochondral ossification | 0.00399368 | 9 |
| 50. | WP581 EPO receptor signaling | 0.00399368 | 7 |
| 51. | hsa00480 Glutathione metabolism | 0.00399368 | 6 |
| 52. | hsa01100 Metabolic pathways | 0.00399368 | 11 |
| 53. | hsa04621 NOD-like receptor signaling pathway | 0.00399368 | 8 |
| 54. | hsa05332 Graft versus host disease | 0.00399368 | 5 |
| 55. | P00026 Heterotrimeric G protein signaling pathway Gi alpha and Gs alpha mediated pathway | 0.0040725 | 8 |
| 56. | WP304 Kit receptor signaling pathway | 0.00423824 | 9 |
| 57. | hsa04720 Long-term potentiation | 0.00423824 | 8 |
| 58. | P00052 TGF beta signaling pathway | 0.00440553 | 9 |
| 59. | hsa00280 Valine leucine and isoleucine degradation | 0.00473855 | 6 |
| 60. | hsa04120 Ubiquitin-mediated proteolysis | 0.00475838 | 9 |
| 61. | hsa04520 Adherens junction | 0.00484441 | 10 |
| 62. | WP673 ErbB signaling pathway | 0.00486452 | 9 |
| 63. | hsa05010 Alzheimers disease | 0.00502782 | 9 |
| 64. | hsa05217 Basal cell carcinoma | 0.00502782 | 7 |
| 65. | P00004 Alzheimer disease presenilin pathway | 0.00504547 | 9 |
| 66. | WP15 selenium | 0.00504547 | 8 |
| 67. | WP500 Glycogen metabolism | 0.00504547 | 6 |
| 68. | P00060 Ubiquitin proteasome pathway | 0.00509898 | 7 |
| 69. | WP366 TGF beta signaling pathway1 | 0.00556393 | 10 |
| 70. | WP382 MAPK signaling pathway | 0.00556393 | 10 |
| 71. | WP690 Polyol pathway | 0.00556393 | 3 |
| 72. | hsa00310 Lysine degradation | 0.00556393 | 7 |
| 73. | hsa05210 Colorectal cancer | 0.00556393 | 10 |
| 74. | hsa05218 Melanoma | 0.00556393 | 10 |
| 75. | hsa05212 Pancreatic cancer | 0.0057502 | 10 |
| 76. | WP98 Prostaglandin synthesis and regulation | 0.00576084 | 6 |
| 77. | hsa03420 Nucleotide excision repair | 0.00576084 | 6 |
| 78. | P00044 Nicotinic acetylcholine receptor signaling pathway | 0.00585757 | 7 |
| 79. | P02739 De novo pyrimidine deoxyribonucleotide biosynthesis | 0.00585757 | 5 |
| 80. | P04376 5HT4 type receptor mediated signaling pathway | 0.00585757 | 5 |
| 81. | P04379 Beta3 adrenergic receptor signaling pathway | 0.00585757 | 5 |
| 82. | hsa04114 Oocyte meiosis | 0.00585757 | 9 |
| 83. | P00020 FAS signaling pathway | 0.00592564 | 7 |
| 84. | hsa04916 Melanogenesis | 0.00592564 | 8 |
| 85. | hsa04920 Adipocytokine signaling pathway | 0.00592564 | 8 |
| 86. | P00018 EGF receptor signaling pathway | 0.00593991 | 9 |
| 87. | WP1591 Heart development | 0.00597612 | 8 |
| 88. | WP405 Eukaryotic transcription initiation | 0.00597612 | 6 |
| 89. | WP410 Diurnally regulated genes with circadian orthologs | 0.00597612 | 7 |
| 90. | hsa00510 N glycan biosynthesis | 0.00597612 | 6 |
| 91. | hsa00670 One carbon pool by folate | 0.00597612 | 5 |
| 92. | hsa00970 Aminoacyl tRNA biosynthesis | 0.00597612 | 6 |
| 93. | hsa04146 Peroxisome | 0.00597612 | 6 |
| 94. | hsa04640 Hematopoietic cell lineage | 0.00597612 | 6 |
| 95. | hsa05214 Glioma | 0.00597612 | 10 |
| 96. | hsa05322 Systemic lupus erythematosus | 0.00597612 | 8 |
| 97. | P04380 Cortocotropin-releasing factor receptor signaling pathway | 0.00612473 | 5 |
| 98. | hsa04650 Natural killer cell-mediated cytotoxicity | 0.00612473 | 8 |
| 99. | hsa04330 Notch signaling pathway | 0.00615425 | 7 |
| 100. | P00012 Cadherin signaling pathway | 0.00643522 | 8 |
| 101. | WP111 Electron transport chain | 0.00643522 | 7 |
| 102. | WP134 Pentose phosphate pathway | 0.00643522 | 4 |
| 103. | WP2064 Neural crest differentiation | 0.00643522 | 8 |
| 104. | WP51 Regulation of actin cytoskeleton | 0.00643522 | 9 |
| 105. | hsa00770 Pantothenate and CoA (coenzyme A) biosynthesis | 0.00643522 | 4 |
| 106. | hsa04130 SNARE interactions in vesicular transport | 0.00643522 | 5 |
| 107. | hsa04340 Hedgehog signaling pathway | 0.00643522 | 6 |
| 108. | P00036 Interleukin signaling pathway | 0.00663403 | 9 |
| 109. | P02773 S-adenosylmethionine biosynthesis | 0.00663403 | 3 |
| 110. | hsa05016 Huntingtons disease | 0.00689596 | 9 |
| 111. | WP615 Senescence and autophagy | 0.00724899 | 10 |
| 112. | hsa00910 Nitrogen metabolism | 0.00732954 | 5 |
| 113. | P00054 Toll receptor signaling pathway | 0.00746098 | 7 |
| 114. | P00016 Cytoskeletal regulation by Rho GTPase | 0.00765971 | 8 |
| 115. | WP2037 Prolactin signaling pathway | 0.00765971 | 9 |
| 116. | hsa00240 Pyrimidine metabolism | 0.00773805 | 7 |
| 117. | P04393 Ras pathway | 0.00780657 | 8 |
| 118. | hsa00330 Arginine and proline metabolism | 0.00780657 | 6 |
| 119. | hsa05320 Autoimmune thyroid disease | 0.00780657 | 4 |
| 120. | WP2034 Leptin signaling pathway | 0.00787498 | 9 |
| 121. | hsa04010 MAPK signaling pathway | 0.00787498 | 10 |
| 122. | hsa04810 Regulation of actin cytoskeleton | 0.00787498 | 9 |
| 123. | hsa04062 Chemokine signaling pathway | 0.00818121 | 9 |
| 124. | P00046 Oxidative stress response | 0.00818745 | 7 |
| 125. | WP1539 Angiogenesis | 0.00842384 | 6 |
| 126. | WP306 Focal adhesion | 0.00842384 | 10 |
| 127. | WP408 Oxidative stress | 0.00842384 | 6 |
| 128. | WP706 SIDS susceptibility pathways | 0.00842384 | 7 |
| 129. | hsa04666 FC gamma R-mediated phagocytosis | 0.00842384 | 8 |
| 130. | WP254 Apoptosis | 0.00848415 | 9 |
| 131. | P00037 Ionotropic glutamate receptor pathway | 0.00867731 | 4 |
| 132. | P02728 Arginine biosynthesis | 0.00867731 | 3 |
| 133. | P02775 Salvage pyrimidine ribonucleotides | 0.00867731 | 3 |
| 134. | P06587 Nicotine pharmacodynamics pathway | 0.00867731 | 3 |
| 135. | WP179 Cell cycle | 0.00867731 | 10 |
| 136. | WP453 Inflammatory response pathway | 0.00867731 | 5 |
| 137. | hsa00053 Ascorbate and aldarate metabolism | 0.00867731 | 4 |
| 138. | hsa00190 Oxidative phosphorylation | 0.00867731 | 7 |
| 139. | hsa05330 Allograft rejection | 0.00867731 | 4 |
| 140. | hsa05410 Hypertrophic cardiomyopathy (HCM) | 0.00867731 | 7 |
| 141. | WP1601 Fluoropyrimidine activity | 0.00877411 | 6 |
| 142. | P00014 Cholesterol biosynthesis | 0.00903745 | 4 |
| 143. | P04377 Beta1 adrenergic receptor signaling pathway | 0.00903745 | 5 |
| 144. | P04378 Beta2 adrenergic receptor signaling pathway | 0.00903745 | 5 |
| 145. | WP107 Translation factors | 0.00903745 | 7 |
| 146. | WP143 Fatty acid beta oxidation | 0.00903745 | 5 |
| 147. | WP176 Folate metabolism | 0.00903745 | 7 |
| 148. | WP236 Adipogenesis | 0.00903745 | 9 |
| 149. | hsa04012 ErbB signaling pathway | 0.00903745 | 9 |
| 150. | hsa04370 VEGF signaling pathway | 0.00903745 | 8 |
| 151. | hsa05414 Dilated cardiomyopathy | 0.00903745 | 7 |
| 152. | WP2118 Arrhythmogenic right ventricular cardiomyopathy | 0.0094442 | 7 |
| 153. | hsa05412 Arrhythmogenic right ventricular cardiomyopathy ARVC | 0.0094442 | 7 |
| 154. | hsa04670 Leukocyte transendothelial migration | 0.00954132 | 8 |
| 155. | P00006 Apoptosis signaling pathway | 0.00971002 | 9 |
| 156. | P00034 Integrin signalling pathway | 0.00971002 | 9 |
| 157. | WP430 Statin pathway | 0.00971002 | 5 |
| 158. | WP53 Id Signaling pathway | 0.00971002 | 5 |
| 159. | hsa00061 Fatty acid biosynthesis | 0.00971002 | 4 |
| 160. | hsa00071 Fatty acid metabolism | 0.00971002 | 5 |
| 161. | hsa00340 Histidine metabolism | 0.00971002 | 4 |
| 162. | hsa01040 Biosynthesis of unsaturated fatty acids | 0.00971002 | 5 |
| 163. | WP466 DNA replication | 0.0099287 | 6 |
| 164. | hsa05215 Prostate cancer | 0.0099287 | 10 |
| 165. | WP35 G protein signaling pathways | 0.0103576 | 7 |
| 166. | hsa00830 Retinol metabolism | 0.0103959 | 4 |
| 167. | P02771 Pyrimidine metabolism | 0.010496 | 3 |
| 168. | P00013 Cell cycle | 0.0108584 | 6 |
| 169. | WP28 Selenium metabolism and selenoproteins | 0.0108584 | 5 |
| 170. | hsa00650 Butanoate metabolism | 0.0108584 | 5 |
| 171. | hsa03050 Proteasome | 0.0108584 | 6 |
| 172. | P00058 mRNA splicing | 0.0111808 | 2 |
| 173. | WP289 Myometrial relaxation and contraction pathways | 0.0111808 | 8 |
| 174. | WP69 TCR signaling pathway | 0.0111808 | 8 |
| 175. | hsa04110 Cell cycle | 0.0111808 | 10 |
| 176. | hsa04672 Intestinal immune network for IgA production | 0.0111808 | 4 |
| 177. | hsa05012 Parkinsons disease | 0.0119769 | 7 |
| 178. | hsa04360 Axon guidance | 0.0120646 | 8 |
| 179. | hsa04510 Focal adhesion | 0.0123289 | 10 |
| 180. | P00027 Heterotrimeric G protein signaling pathway Gq alpha and Go alpha mediated pathway | 0.0124732 | 6 |
| 181. | P00005 Angiogenesis | 0.0126857 | 9 |
| 182. | WP268 Notch signaling pathway | 0.0127564 | 6 |
| 183. | WP391 Mitochondrial gene expression | 0.0127564 | 5 |
| 184. | WP411 mRNA processing | 0.0127564 | 8 |
| 185. | WP437 EGF–EGFR signaling pathway | 0.0127564 | 9 |
| 186. | hsa00350 Tyrosine metabolism | 0.0127564 | 4 |
| 187. | hsa03030 DNA replication | 0.0127564 | 5 |
| 188. | hsa04320 Dorso ventral axis formation | 0.0127564 | 6 |
| 189. | P02736 Coenzyme A biosynthesis | 0.0131869 | 3 |
| 190. | hsa04080 Neuroactive ligand receptor interaction | 0.0133566 | 5 |
| 191. | WP314 FAS pathway and stress induction of HSP regulation | 0.0135744 | 7 |
| 192. | hsa00230 Purine metabolism | 0.0135744 | 7 |
| 193. | hsa03018 RNA degradation | 0.0139554 | 6 |
| 194. | P00017 DNA replication | 0.0140213 | 5 |
| 195. | hsa00564 Glycerophospholipid metabolism | 0.0140213 | 5 |
| 196. | P00021 FGF signaling pathway | 0.0143486 | 8 |
| 197. | P00047 PDGF signaling pathway | 0.0143486 | 8 |
| 198. | P00059 p53 pathway | 0.0143486 | 9 |
| 199. | P02768 Proline biosynthesis | 0.0143486 | 2 |
| 200. | P05918 p38 MAPK pathway | 0.0143486 | 2 |
| 201. | WP3 Keap1 Nrf2 | 0.0143486 | 4 |
| 202. | hsa00250 Alanine aspartate and glutamate metabolism | 0.0143486 | 5 |
| 203. | hsa00410 beta Alanine metabolism | 0.0143486 | 4 |
| 204. | hsa04914 Progesterone mediated oocyte maturation | 0.0143486 | 8 |
| 205. | hsa04940 Type I diabetes mellitus | 0.0143486 | 4 |
| 206. | WP1403 AMPK signaling | 0.0148787 | 8 |
| 207. | P00033 Insulin IGF pathway protein kinase B signaling cascade | 0.0152041 | 7 |
| 208. | WP49 IL 2 Signaling pathway | 0.0152041 | 7 |
| 209. | hsa00640 Propanoate metabolism | 0.0152041 | 5 |
| 210. | P00032 Insulin IGF pathway mitogen activated protein kinase kinase MAP kinase cascade | 0.0160261 | 6 |
| 211. | P05912 Dopamine receptor-mediated signaling pathway | 0.0160261 | 3 |
| 212. | hsa00040 Pentose and glucuronate interconversions | 0.0160261 | 3 |
| 213. | P00057 Wnt signaling pathway | 0.0165674 | 9 |
| 214. | WP45 G1 to S cell cycle control | 0.0165674 | 9 |
| 215. | hsa00590 Arachidonic acid metabolism | 0.0180789 | 5 |
| 216. | WP23 B Cell receptor signaling pathway | 0.0182671 | 8 |
| 217. | P02769 Purine metabolism | 0.0183873 | 2 |
| 218. | WP501 GPCRs class C metabotropic glutamate pheromone | 0.0183873 | 2 |
| 219. | hsa04912 GnRH signaling pathway | 0.0183873 | 7 |
| 220. | hsa03410 Base excision repair | 0.0188826 | 5 |
| 221. | WP530 Cytokines and inflammatory response | 0.01921 | 4 |
| 222. | WP286 IL-3 signaling pathway | 0.0198771 | 7 |
| 223. | P02743 Formyltetrahydroformate biosynthesis | 0.0199035 | 3 |
| 224. | hsa00790 Folate biosynthesis | 0.0199035 | 3 |
| 225. | hsa04512 ECM–receptor interaction | 0.0199035 | 6 |
| 226. | hsa04740 Olfactory transduction | 0.0204677 | 4 |
| 227. | WP244 Alpha 6 beta 4 signaling pathway | 0.0206888 | 6 |
| 228. | P00029 Huntington disease | 0.0209916 | 8 |
| 229. | P00053 T-cell activation | 0.0209916 | 7 |
| 230. | hsa04620 Toll-like receptor signaling pathway | 0.0209916 | 7 |
| 231. | P00043 Muscarinic acetylcholine receptor 2 and 4 signaling pathway | 0.0215618 | 5 |
| 232. | P04386 Histamine H2 receptor-mediated signaling pathway | 0.0215618 | 4 |
| 233. | WP2059 Alzheimers disease | 0.0215618 | 7 |
| 234. | WP75 Toll-like receptor signaling | 0.0215618 | 7 |
| 235. | WP205 IL-7 signaling pathway | 0.0221478 | 6 |
| 236. | hsa04910 Insulin signaling pathway | 0.0229117 | 8 |
| 237. | hsa05223 Non-small cell lung cancer | 0.0229117 | 8 |
| 238. | P00010 B cell activation | 0.0229996 | 6 |
| 239. | P00035 Interferon gamma signaling pathway | 0.0237303 | 5 |
| 240. | P02745 Glutamine glutamate conversion | 0.0237303 | 3 |
| 241. | WP322 Osteoblast | 0.0237303 | 3 |
| 242. | hsa04710 Circadian rhythm mammal | 0.0237303 | 3 |
| 243. | P00007 Axon guidance mediated by semaphorins | 0.0240705 | 4 |
| 244. | WP299 Nuclear receptors in lipid metabolism and toxicity | 0.0240705 | 4 |
| 245. | WP710 DNA damage response only ATM-dependent | 0.0240705 | 9 |
| 246. | hsa05220 Chronic myeloid leukemia | 0.0240705 | 9 |
| 247. | P00056 VEGF signaling pathway | 0.0242799 | 7 |
| 248. | WP422 MAPK cascade | 0.0243685 | 5 |
| 249. | hsa05211 Renal cell carcinoma | 0.0254042 | 8 |
| 250. | WP384 Apoptosis modulation by HSP70 | 0.0255004 | 4 |
| 251. | WP1602 Nicotine activity on dopaminergic neurons | 0.025681 | 3 |
| 252. | hsa04270 Vascular smooth muscle contraction | 0.0264143 | 6 |
| 253. | hsa00380 Tryptophan metabolism | 0.0271629 | 4 |
| 254. | hsa04730 Long-term depression | 0.0274791 | 6 |
| 255. | P00022 General transcription by RNA polymerase I | 0.0280057 | 3 |
| 256. | P05734 Synaptic vesicle trafficking | 0.0280057 | 3 |
| 257. | WP259 Nifedipine activity | 0.0280057 | 3 |
| 258. | hsa00500 Starch and sucrose metabolism | 0.0285496 | 4 |
| 259. | hsa05221 Acute myeloid leukemia | 0.0285496 | 7 |
| 260. | WP138 Androgen receptor signaling pathway | 0.0299755 | 9 |
| 261. | WP481 Insulin signaling | 0.0313275 | 8 |
| 262. | P02788 Xanthine and guanine salvage pathway | 0.0316996 | 2 |
| 263. | P00025 Hedgehog signaling pathway | 0.0318924 | 4 |
| 264. | P00031 Inflammation mediated by chemokine and cytokine signaling pathway | 0.0318924 | 8 |
| 265. | WP167 Eicosanoid synthesis | 0.0318924 | 4 |
| 266. | WP313 Signaling of hepatocyte growth factor receptor | 0.0318924 | 6 |
| 267. | hsa04610 Complement and coagulation cascades | 0.0318924 | 4 |
| 268. | P00048 PI3 kinase pathway | 0.0322689 | 7 |
| 269. | hsa04142 Lysosome | 0.0326171 | 6 |
| 270. | hsa00062 Fatty acid elongation in mitochondria | 0.0326488 | 3 |
| 271. | hsa00601 Glycosphingolipid biosynthesis lacto and neolacto series | 0.0326488 | 3 |
| 272. | hsa03320 PPAR (peroxisome proliferator-activated receptor) signaling pathway | 0.0327495 | 5 |
| 273. | hsa04260 Cardiac muscle contraction | 0.0327495 | 5 |
| 274. | hsa05219 Bladder cancer | 0.0331809 | 8 |
| 275. | WP712 Estrogen signaling pathway | 0.0333459 | 6 |
| 276. | hsa04660 T-cell receptor signaling pathway | 0.0338532 | 7 |
| 277. | P00055 Transcription regulation by bZIP transcription factor | 0.0340133 | 5 |
| 278. | P04373 5HT1 type receptor-mediated signaling pathway | 0.0344438 | 4 |
| 279. | WP364 IL-6 signaling pathway | 0.0344438 | 6 |
| 280. | WP698 Glucuronidation | 0.0344438 | 3 |
| 281. | hsa00900 Terpenoid backbone biosynthesis | 0.0344438 | 3 |
| 282. | hsa02010 ABC transporters | 0.0344438 | 4 |
| 283. | hsa04950 Maturity onset diabetes of the young | 0.0344438 | 3 |
| 284. | WP545 Complement activation classical pathway | 0.0353132 | 2 |
| 285. | P05731 GABA B receptor II signaling | 0.0364755 | 4 |
| 286. | WP127 IL-5 signaling pathway | 0.0364951 | 6 |
| 287. | P02740 De novo pyrimidine ribonucleotides biosythesis | 0.0371447 | 3 |
| 288. | P04372 5 Hydroxytryptamine degredation | 0.0371447 | 3 |
| 289. | hsa00561 Glycerolipid metabolism | 0.0383284 | 4 |
| 290. | hsa03010 Ribosome | 0.0388605 | 7 |
| 291. | hsa05222 Small cell lung cancer | 0.0396732 | 8 |
| 292. | P05730 Endogenous cannabinoid signaling | 0.0400627 | 3 |
| 293. | WP734 Serotonin receptor 4 6 7 and NR3C signaling | 0.0402265 | 4 |
| 294. | WP528 Acetylcholine synthesis | 0.0404426 | 2 |
| 295. | WP288 NLR proteins | 0.0429284 | 3 |
| 296. | WP727 Monoamine transport | 0.0429284 | 3 |
| 297. | hsa00760 Nicotinate and nicotinamide metabolism | 0.0429284 | 3 |
| 298. | WP195 IL-1 signaling pathway | 0.0430455 | 6 |
| 299. | hsa05200 Pathways in cancer | 0.0439735 | 10 |
| 300. | WP524 G13 Signaling pathway | 0.0443451 | 5 |
| 301. | WP2256 Integrated pancreatic cancer pathway | 0.0445963 | 9 |
| 302. | hsa00120 Primary bile acid biosynthesis | 0.0459705 | 2 |
| 303. | P04394 Thyrotropin-releasing hormone receptor signaling pathway | 0.0460334 | 4 |
| 304. | WP26 Signal transduction of S1P | 0.0460334 | 4 |
| 305. | hsa00020 Citrate cycle TCA cycle | 0.0460334 | 4 |
| 306. | hsa00030 Pentose phosphate pathway | 0.0460334 | 4 |
| 307. | hsa04662 B-cell receptor signaling pathway | 0.0470304 | 6 |
| 308. | WP170 Nuclear receptors | 0.0473595 | 5 |
| 309. | P00039 Metabotropic glutamate receptor group III pathway | 0.0479412 | 4 |
| 310. | P04391 Oxytocin receptor mediated signaling pathway | 0.0479412 | 4 |
| 311. | WP477 Cytoplasmic ribosomal proteins | 0.0479412 | 7 |
| 312. | hsa03450 Non-homologous end joining | 0.0479412 | 3 |
| 313. | hsa04614 Renin angiotensin system | 0.0479412 | 3 |

**Table S10.** Results of enrichment analysis by miRSystem for 11 significantly changed miRNAs in HHH vs. NNN and sorted by p value. ‘PID’ indicates Pathway Interaction Database.

| Category | Term | Union miRNAs | Raw p value | Empirical p value |
| --- | --- | --- | --- | --- |
| KEGG | T_CELL_RECEPTOR_SIGNALING_PATHWAY | 7 | 6.68E-04 | 3.51E-04 |
| KEGG | B_CELL_RECEPTOR_SIGNALING_PATHWAY | 6 | 1.39E-03 | 1.23E-03 |
| REACTOME | G1_S_TRANSITION | 6 | 7.75E-05 | 2.78E-03 |
| PID | IL6-MEDIATED_SIGNALING_EVENTS | 7 | 2.07E-04 | 3.09E-03 |
| REACTOME | DEVELOPMENTAL_BIOLOGY | 9 | 1.20E-03 | 3.14E-03 |
| REACTOME | NEURONAL_SYSTEM | 6 | 2.42E-03 | 3.41E-03 |
| PID | REELIN_SIGNALING_PATHWAY | 5 | 8.65E-04 | 3.75E-03 |
| BIOCARTA | BIOCARTA_NFAT_PATHWAY | 6 | 4.14E-04 | 3.84E-03 |
| KEGG | WNT_SIGNALING_PATHWAY | 8 | 2.71E-03 | 4.23E-03 |
| PID | SIGNALING_EVENTS_MEDIATED_BY_ FOCAL_ADHESION_KINASE | 7 | 6.05E-03 | 5.00E-03 |
| KEGG | PATHWAYS_IN_CANCER | 9 | 1.12E-03 | 5.26E-03 |
| REACTOME | MITOTIC_G1-G1_S_PHASES | 6 | 2.44E-04 | 5.29E-03 |
| REACTOME | KINESINS | 3 | 1.29E-02 | 5.86E-03 |
| REACTOME | AXON_GUIDANCE | 6 | 6.53E-03 | 6.19E-03 |
| KEGG | JAK-STAT_SIGNALING_PATHWAY | 8 | 3.09E-03 | 6.81E-03 |
| PID | IL2_SIGNALING_EVENTS_MEDIATED_BY_ STAT5 | 5 | 9.55E-04 | 7.25E-03 |
| PID | LKB1_SIGNALING_EVENTS | 6 | 3.22E-03 | 7.26E-03 |
| PID | REGULATION_OF_RAC1_ACTIVITY | 2 | 2.40E-02 | 7.40E-03 |
| KEGG | SMALL_CELL_LUNG_CANCER | 5 | 2.08E-03 | 8.28E-03 |
| BIOCARTA | BIOCARTA_TCR_PATHWAY | 4 | 3.41E-03 | 8.41E-03 |
| BIOCARTA | BIOCARTA_FCER1_PATHWAY | 4 | 2.03E-03 | 8.84E-03 |
| BIOCARTA | BIOCARTA_WNT_PATHWAY | 7 | 6.27E-04 | 8.85E-03 |
| REACTOME | DNA_REPLICATION | 5 | 1.77E-03 | 9.12E-03 |
| PID | FOXM1_TRANSCRIPTION_FACTOR_ NETWORK | 4 | 1.44E-04 | 9.20E-03 |
| BIOCARTA | BIOCARTA_GSK3_PATHWAY | 7 | 7.01E-04 | 9.78E-03 |
| REACTOME | S_PHASE | 6 | 7.83E-04 | 9.93E-03 |
| REACTOME | CELL_CYCLE_MITOTIC | 6 | 1.23E-03 | 1.16E-02 |
| PID | VALIDATED_TARGETS_OF_C-MYC_TRANSCRIPTIONAL_ACTIVATION | 6 | 1.83E-03 | 1.21E-02 |
| REACTOME | HEMOSTASIS | 9 | 2.78E-03 | 1.23E-02 |
| REACTOME | TRANSCRIPTIONAL_REGULATION_OF_ WHITE_ADIPOCYTE_DIFFERENTIATION | 7 | 9.57E-03 | 1.57E-02 |
| PID | SIGNALING_EVENTS_MEDIATED_BY_STEM_CELL_FACTOR_RECEPTOR_(C-KIT) | 5 | 4.51E-03 | 1.59E-02 |
| REACTOME | POST_NMDA_RECEPTOR_ACTIVATION_ EVENTS | 4 | 1.86E-02 | 1.59E-02 |
| REACTOME | FACTORS_INVOLVED_IN_ MEGAKARYOCYTE_DEVELOPMENT_AND_ PLATELET_PRODUCTION | 5 | 8.03E-03 | 1.59E-02 |
| PID | TRK_RECEPTOR_SIGNALING_MEDIATED_ BY_PI3K_AND_PLC-GAMMA | 5 | 1.37E-03 | 1.63E-02 |
| PID | PRESENILIN_ACTION_IN_NOTCH_AND_ WNT_SIGNALING | 7 | 3.22E-03 | 1.80E-02 |
| KEGG | CELL_CYCLE | 6 | 1.22E-03 | 1.81E-02 |
| KEGG | MAPK_SIGNALING_PATHWAY | 6 | 1.76E-03 | 1.87E-02 |
| REACTOME | TRANSCRIPTION-COUPLED_NER_(TC-NER) | 4 | 2.84E-03 | 1.88E-02 |
| REACTOME | CREB_PHOSPHORYLATION_THROUGH_ THE_ACTIVATION_OF_RAS | 4 | 1.29E-02 | 1.98E-02 |
| KEGG | NUCLEOTIDE_EXCISION_REPAIR | 4 | 2.84E-03 | 2.00E-02 |
| REACTOME | NUCLEOTIDE_EXCISION_REPAIR | 4 | 3.83E-03 | 2.02E-02 |
| BIOCARTA | BIOCARTA_ERK_PATHWAY | 3 | 1.38E-02 | 2.12E-02 |
| PID | REGULATION_OF_CDC42_ACTIVITY | 2 | 1.56E-02 | 2.54E-02 |
| REACTOME | CD28_CO-STIMULATION | 3 | 1.66E-02 | 2.59E-02 |
| KEGG | NEUROTROPHIN_SIGNALING_PATHWAY | 5 | 8.45E-03 | 2.64E-02 |
| REACTOME | PI3K_AKT_ACTIVATION | 5 | 1.75E-03 | 2.65E-02 |
| PID | IL23-MEDIATED_SIGNALING_EVENTS | 5 | 2.28E-02 | 2.75E-02 |
| PID | DIRECT_P53_EFFECTORS | 8 | 1.08E-02 | 2.77E-02 |
| BIOCARTA | BIOCARTA_MET_PATHWAY | 5 | 2.28E-02 | 2.82E-02 |
| PID | EPHB_FORWARD_SIGNALING | 4 | 2.18E-02 | 2.85E-02 |
| REACTOME | SIGNALING_BY_SCF-KIT | 5 | 1.31E-02 | 3.07E-02 |
| REACTOME | SYNTHESIS_OF_DNA | 5 | 3.32E-03 | 3.11E-02 |
| REACTOME | SIGNALING_BY_INTERLEUKINS | 6 | 2.77E-02 | 3.17E-02 |
| BIOCARTA | BIOCARTA_G1_PATHWAY | 3 | 1.38E-02 | 3.41E-02 |
| BIOCARTA | BIOCARTA_VIP_PATHWAY | 3 | 1.29E-02 | 3.42E-02 |
| KEGG | FOCAL_ADHESION | 5 | 8.25E-03 | 3.44E-02 |
| BIOCARTA | BIOCARTA_BCR_PATHWAY | 2 | 2.07E-02 | 3.70E-02 |
| REACTOME | TRANSMISSION_ACROSS_CHEMICAL_ SYNAPSES | 5 | 2.86E-02 | 3.79E-02 |
| REACTOME | G0_AND_EARLY_G1 | 4 | 1.11E-02 | 3.92E-02 |
| PID | AP-1_TRANSCRIPTION_FACTOR_NETWORK | 5 | 9.57E-03 | 4.06E-02 |
| KEGG | ALZHEIMER'S_DISEASE | 4 | 7.50E-04 | 4.17E-02 |
| KEGG | AMINOACYL-TRNA_BIOSYNTHESIS | 5 | 7.54E-03 | 4.30E-02 |
| KEGG | FRUCTOSE_AND_MANNOSE_METABOLISM | 3 | 1.96E-02 | 4.30E-02 |
| REACTOME | OPIOID_SIGNALLING | 3 | 1.40E-02 | 4.32E-02 |
| KEGG | BASE_EXCISION_REPAIR | 3 | 1.86E-02 | 4.33E-02 |
| KEGG | ENDOMETRIAL_CANCER | 6 | 4.51E-03 | 4.36E-02 |
| PID | PDGFR-BETA_SIGNALING_PATHWAY | 4 | 4.10E-02 | 4.38E-02 |
| PID | SHP2_SIGNALING | 5 | 4.99E-03 | 4.45E-02 |
| PID | P53_PATHWAY | 5 | 6.05E-03 | 4.50E-02 |
| KEGG | ERBB_SIGNALING_PATHWAY | 6 | 1.72E-02 | 4.58E-02 |
| PID | AURORA_A_SIGNALING | 3 | 1.05E-03 | 4.59E-02 |
| REACTOME | GPVI-MEDIATED_ACTIVATION_CASCADE | 3 | 1.86E-02 | 4.71E-02 |
| KEGG | COLORECTAL_CANCER | 6 | 7.23E-03 | 4.74E-02 |
| REACTOME | PLATELET_ACTIVATION_SIGNALING_AND_AGGREGATION | 8 | 9.23E-03 | 4.80E-02 |
| REACTOME | GAB1_SIGNALOSOME | 4 | 2.51E-02 | 4.82E-02 |
| REACTOME | DOWNSTREAM_TCR_SIGNALING | 6 | 4.12E-02 | 4.84E-02 |
